# Rhodium diamidobenzene complexes: a tale of different substituents on the diamidobenzene ligand†

**DOI:** 10.1039/d2sc03227a

**Published:** 2022-07-21

**Authors:** Simon Suhr, Robert Walter, Julia Beerhues, Uta Albold, Biprajit Sarkar

**Affiliations:** Lehrstuhl für Anorganische Koordinationschemie, Institut für Anorganische Chemie, Universität Stuttgart Pfaffenwaldring 55 70569 Stuttgart Germany; Institut für Chemie und Biochemie, Freie Universität Berlin Fabeckstr. 34-36 14195 Berlin Germany

## Abstract

Diamidobenzene ligands are a prominent class of redox-active ligands owing to their electron reservoir behaviour, as well as the possibility of tuning the steric and the electronic properties of such ligands through the substituents on the N-atoms of the ligands. In this contribution, we present Rh(iii) complexes with four differently substituted diamidobenzene ligands. By using a combination of crystallography, NMR spectroscopy, electrochemistry, UV-vis-NIR/EPR spectroelectrochemistry, and quantum chemical calculations we show that the substituents on the ligands have a profound influence on the bonding, donor, electrochemical and spectroscopic properties of the Rh complexes. We present, for the first time, design strategies for the isolation of mononuclear Rh(ii) metallates whose redox potentials span across more than 850 mV. These Rh(ii) metallates undergo typical metalloradical reactivity such as activation of O_2_ and C–Cl bond activations. Additionally, we also show that the substituents on the ligands dictate the one *versus* two electron nature of the oxidation steps of the Rh complexes. Furthermore, the oxidative reactivity of the metal complexes with a [CH_3_]^+^ source leads to the isolation of a unprecedented, homobimetallic, heterovalent complex featuring a novel π-bonded rhodio-*o*-diiminoquionone. Our results thus reveal several new potentials of the diamidobenzene ligand class in organometallic reactivity and small molecule activation with potential relevance for catalysis.

## Abstract 2

Diamidobenzene ligands are versatile platforms in organometallic Rh-chemistry. They allow the isolation of tunable mononuclear ate-complexes, and the formation of a unprecedented homobimetallic, heterovalent complex.

## Graphical abstract

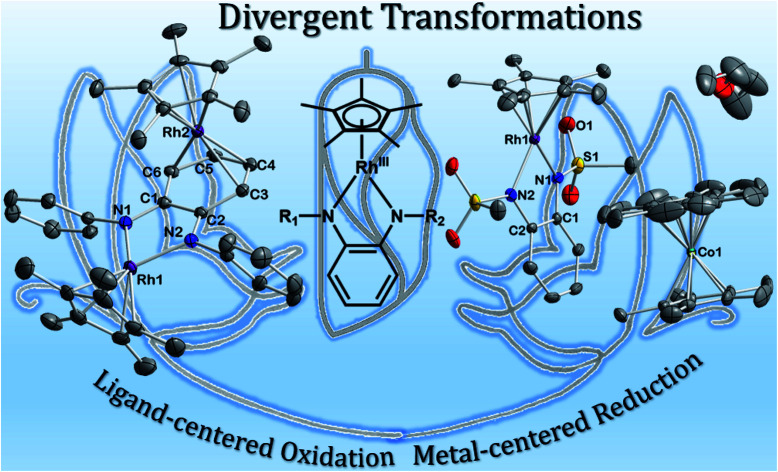

## Introduction

Rhodium half-sandwich complexes are a staple of organometallic chemistry.^1^ The 18 valence electron species [Cp*Rh^i^L_2_] act as prototypical metallobases capable of oxidative addition of electrophiles.^2^ This reactivity has been used maybe most famously in the electro- and photochemical reduction of protons by [Cp*Rh] complexes bearing chelating diimine ligands.^3^ Therefore, the electrochemistry of these systems has been studied extensively and minute details regarding the influence of structure and substitution pattern of the diimines on the redox properties have been reported.^4–7^ In most cases, the electrochemistry is dominated by two-electron events in which both the chelating ligand and the metal center participate due to the covalent nature of the metal–ligand bond.

**Scheme 1:**
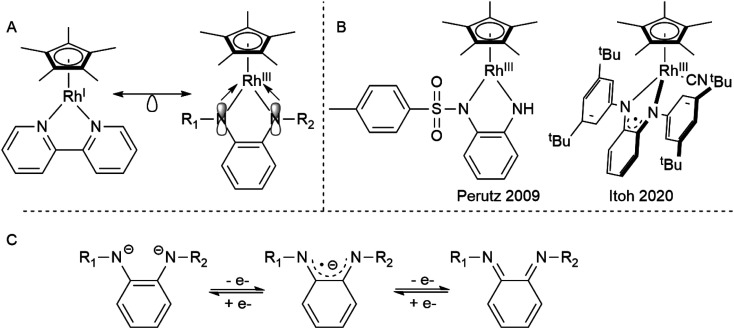
(A) The isoelectronic analogy between Rh^i^ diimine and Rh^iii^ diamido complexes. (B) Catalytically active Rh diamidobenzene complexes. (C) The different oxidation states attainable by diamidobenzenes.

Diamidobenzene complexes of [Cp*Rh^iii^] can be considered isoelectronic analogues to their diimine [Cp*Rh^i^] counterparts, with electron density shifted from the metal center to the chelating π-donor ligand. However, since their first description by Maitlis in 1978,^8^ only very few studies (Scheme 1) have been concerned with the reactivity and electrochemistry of the diamidobenzene systems – their use in aminations reported by Itoh^9^ and as model compounds for transfer hydrogenation as described by Perutz^10,11^ are notable exceptions. This scarcity is surprising, considering that diamidobenzenes as redox-active ligands^12–17^ may impart novel reactivity on rhodium half-sandwich complexes. Diamidobenzene ligands can support three different oxidation states^18–24^ and therefore offer access to an open-shell reactivity which may complement the more commonplace two-electron redox reactions of Rh half-sandwich complexes.^25,26^ Particularly in the diimine state, they form strong, highly covalent bonds to transition metals.^27–29^ Additionally, the [NR] handle allows steric and electronic tuning in the direct vicinity of the reaction center. This has also enabled the development of redox-active tri^30–34^- and tetradentate^35–38^ ligand systems based on diamidobenzene. In this context, it is worthwhile mentioning that a majority of work that deals with such ligands has focussed on R = aryl substituents on the [NR] group.^39–43^ This fact is surprising considering the extreme variations that fundamentally different kinds of R substituents would allow in terms of tuning the steric, and in particular the electronic properties of the compounds. Various studies have proven the utility of *o*-diiminoquinonoid ligands in diverse areas such as photochemistry,^44,45^ molecular magnetism,^46–49^ electrocatalysis^50,51^ and activation of small molecules.^52–55^ A pertinent example is the aforementioned work by the group of Itoh, who used a Rh^iii^ half-sandwich complex with a singly-oxidized, open-shell ligand in the amination of trisylazide.^9^ We have recently shown the ligand-centered redox activity and the ensuing reactivity of diamidobenzene complexes of [Cp*Ir^iii^].^56,57^ Finally, apart from the expected ligand-centered reactivity, the [Rh^iii^] moiety in principle offers access to reductive chemistry (and hence formally mononuclear Rh^ii^ complexes) and thus to a novel class of metal-ate complexes without strong acceptors.^58^ Both reversible and irreversible reductions to anionic species with putative metalloradical character have been described (spectro-)electrochemically for a variety of [Cp*Rh] diimines,^59,60^ but they have generally eluded isolation. Diamidobenzenes offer rational access to such reactive species, as their donor properties are easily tunable *via* the N-bound substituents. In this work, we provide a comprehensive investigation into the structure, bonding, electrochemistry and spectroelectrochemistry of diamidobenzene complexes of [Cp*Rh] and prove that they offer access to rare and uncommon molecular species in organometallic rhodium chemistry. By systematically investigating four differently substituted complexes, we show that

(a) The nature of substituents allows switching between separated one-electron and concerted two-electron oxidations centered on the diamidobenzene ligands.

(b) The complexes offer access to reactive, yet isolable mononuclear Rh^ii^ metalates with tunable potential; such large tuning in isolable mononuclear Rh^ii^ metalates is to the best of our knowledge unprecedented.

(c) An unprecedented reactivity towards electrophilic [CH_3_]^+^ groups allows the direct formation of homobimetallic, heterovalent complexes featuring a novel π-bonded rhodio-*o*-diiminoquionone. To the best of our knowledge, such a reactivity as well as the π-bonded rhodio-*o*-diiminoquionone have never been observed previously.

## Results and discussion

**Scheme 2:**
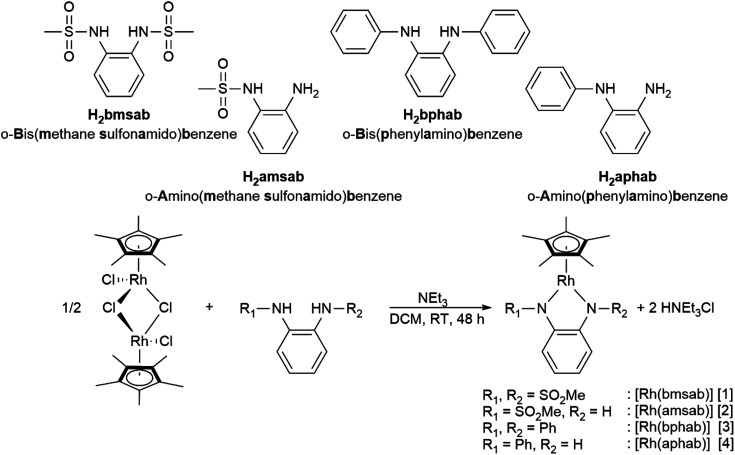
The differently substituted ligands used in this study and the synthetic route to their respective complexes.

We set out to investigate the influence of both symmetry and donor capacity of substituted diamidobenzene ligands on the electrochemical behavior of mononuclear Cp*Rh(iii) complexes. Thus, we chose a set of four ligands (Scheme 2) with substituents on either only one or both N donor atoms. As substituents we compared the strongly electron withdrawing sulfonamido moiety (*F*_SO2Me_ = 0.53) and the mildly electron-withdrawing phenyl group (*F*_Ph_ = 0.12), *F* being the modified Swain–Lupton constant according to Hansch, Leo and Taft.^61^ All four complexes were synthesized by treatment of [Cp*RhCl_2_]_2_ with two equivalents of ligand and an excess of NEt_3_ in DCM (Scheme 2). Following work-up and purification, all compounds were obtained as microcrystalline solids in moderate to good yields. ^1^H- and ^13^C-NMR, EA, HR-MS as well as molecular structures from single crystal X-ray diffraction confirm the expected constitution of all compounds. Furthermore, the ^1^H-NMR spectra in DCM-*d*_2_ indicate diamagnetic compounds, as no paramagnetic contribution to the chemical shift is discernible (Fig. S1–S8†). In case of [1] and [3], the number of signals confirms the expected *C*_2_ symmetry in solution (Fig. S1 and S5†).

**Fig. 1:**
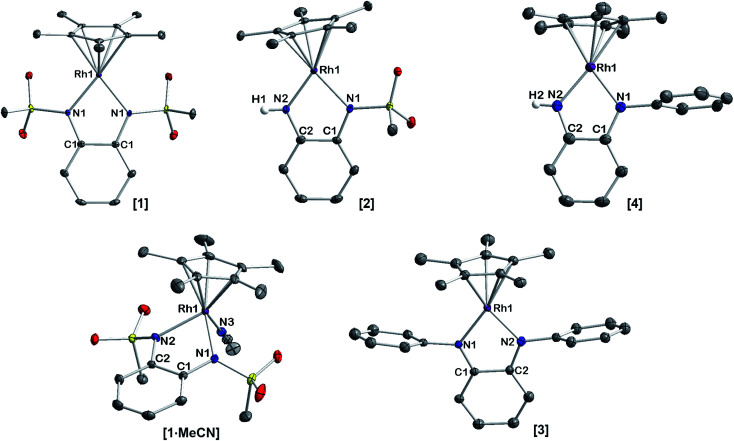
Molecular structures of the neutral complexes [1] to [4] in the crystal. Ellipsoids drawn at 50% probability. Solvent moelcules and H atoms omitted for clarity.

A striking feature of all complexes is their intense color in solution with extinction coefficients ranging from 7 × 10^3^ to 15 × 10^3^ L mol^−1^ cm^−1^ in DCM (Fig. S25†), indicating charge-transfer (CT) transitions and a sizable orbital mixing between ligand and metal. While complexes [2], [3] and [4] display qualitatively similar spectra in different solvents, [1] shows a distinctively different behavior in acetonitrile solution (Fig. S25†), indicating strong interaction with the coordinating solvent. This is revealing of the strongly electron-withdrawing character of the sulfonyl groups on the bmsab ligand, increasing the electrophilicity of the Rh^iii^ center and thus its affinity for MeCN. An inspection of the molecular structures obtained from DCM solutions sheds light on the bonding in complexes [1] to [4] (Fig. 1). All compounds crystallize as coordinatively unsaturated, formally five-coordinate complexes from the non-coordinating solvent. However, dissolution of [1] in acetonitrile and subsequent crystallization yields the coordinatively saturated complex [1·MeCN]. The bond lengths in the diamidobenzene ligand indicate the presence of a fully reduced, dianionic ligand bound to a Rh^iii^ center (Tables 1 and S10†): C–N bond lengths between 1.359(2) and 1.414(2) Å together with C–C bond lengths around 1.40 Å inside the aromatic ring fall into the previously described values for a dianionic diamidobenzene donor.^57,62^ In case of the coordinatively unsaturated compounds, the *N*,*N*-donor is arranged perpendicularly to the plane of the Cp* ligand, with complex [1] showing a slight deviation from perpendicularity. The perpendicular orientation allows for optimal π overlap between the Rh center and the lone pairs of the N donor, leading to electronic saturation and the ligand–metal orbital mixing prerequisite for CT transitions.

**Table 1:** Selected bond lengths in neutral complexes [1]–[4]. All values given in Å

	[1]	[1·MeCN]	[2]	[3]^a^	[4]
C1–N1	1.414(2)	1.414(2)	1.417(3)	1.382(6)	1.371(2)
C2–N2	1.414(2)	1.424(2)	1.366(3)	1.373(7)	1.359(2)
Rh–N1	2.051(2)	2.098(2)	1.937(2)	1.985(4)	1.961(2)
Rh–N2	2.051(2)	2.113(2)	2.075(2)	1.982(5)	1.983(2)

a Complex [3] crystallizes with two different molecules in the asymmetric unit which display slightly different bond lengths. We have given values for the more symmetric molecule, as NMR data indicate *C*_2_ symmetry in solution.

**Fig. 2:**
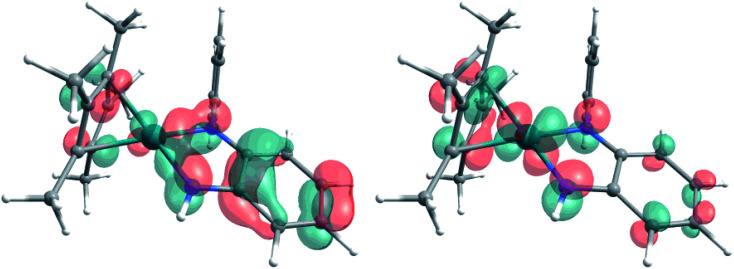
Representative HOMO (left) and LUMO (right) of complex [4], calculated at the TPSSh/def2-TZVP level of theory. Contour value 0.05 A.

**Fig. 3:**
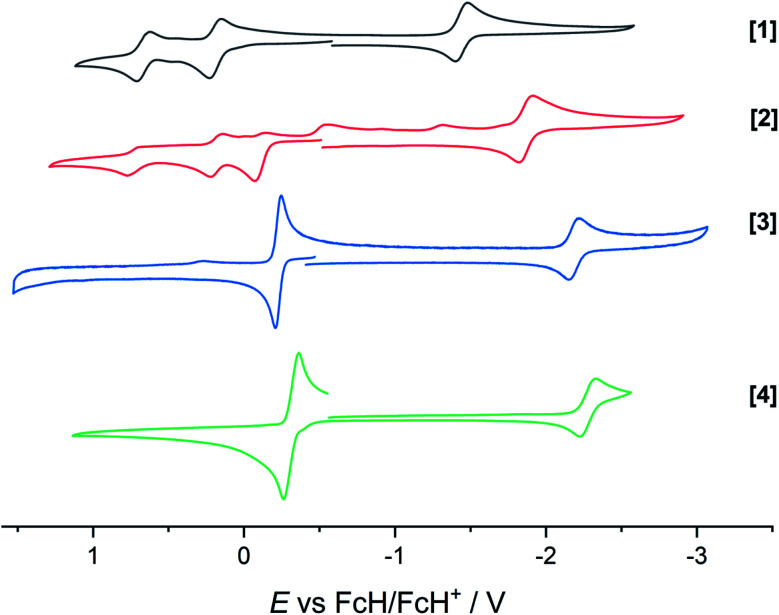
Cyclic voltammograms of complexes [1] to [4]. Measured in dry and degassed MeCN/TBAPF_6_. GC WE, Ag RE, Pt CE. Scan rate 100 mV s^−1^. Current is normalized.

The extent of π donation is strongly influenced by the nature of the N-bound substituents. Evidently, in case of the bis(methane sulfonamide)benzene in [1], the lower limit of π saturation is almost reached, as even a relatively weak donor such as acetonitrile is sufficient to convert [1] into the coordinatively saturated form [1·MeCN]. The N–Rh bond lengths may serve as a metric to gain further insights into the relative donor strength of the other ligands. As expected, the Rh–N bond lengths contract with decreasing electron-withdrawing effect of the N-substituents. In all cases, the NH donor displays the shortest bond length. The expected increase in (π-)donor strength in the series [1] to [4] is thus confirmed by the molecular structures. π-Bonding and antibonding interactions also contribute to the chemically relevant frontier orbitals of the complexes. DFT calculations show qualitatively similar HOMOs and LUMOs for complexes [1] to [4]: while the HOMO has a π-bonding character regarding the metal-diamide interaction with major contributions from the chelating NCCN-moiety, the LUMO is π-antibonding and has a more pronounced 4d character. Fig. 2 shows the frontier orbitals of [4] as a representative example; the corresponding orbitals of [1]–[3] are shown in Fig. S28.† As the HOMO is mostly ligand-centered, one can expect a strong influence of the substitution pattern on the oxidation processes. Indeed, as can be seen from the cyclic voltammograms recorded in acetonitrile (Fig. 3), the four complexes show both qualitatively and quantitatively distinctive electrochemical features. While all complexes display a reversible reduction (*vide infra*), the behavior during the anodic scan depends on the nature of the substituents. Half-wave potentials are listed in Table 2; further details on the electrochemical properties can be found in the ESI, Section 4.† The symmetric sulfonamide complex [1^.^MeCN] shows two well-separated, reversible one-electron oxidations. While asymmetric [2] also shows one-electron processes, these are not reversible, indicating at least one EC mechanism and a complex follow-up chemistry, likely involving the N–H group. Contrasting this behavior, both [3] and [4] only show one feature in the anodic scan: each complex displays a reversible two-electron oxidation. This is in line with results we recently published on the Ir(iii) analogue of [4].^56^ The increasing donor strength, *i.e.* higher electron density on the chelating NCCN moiety, is also reflected in the cathodic shift of the oxidation potentials on going from [1] to [4]. As the DFT calculations indicate, the diamidobenzene moieties are likely the loci of the oxidations.^25^ To verify this assumption, we performed spectro-electrochemical (SEC) measurements on complexes [1·MeCN], [3] and [4] in acetonitrile, as [2] only displayed irreversible oxidations.

**Table 2:** Half-wave potentials as observed in cyclic voltammetry in MeCN/TBAPF_6_. GC WE, Ag RE, Pt CE. All potentials given against the FcH/FcH^+^ redox couple. The third oxidation in [2] is omitted as a follow-up process

	[1]	[2]	[3]	[4]
*E*_1/2_ (1st ox.)/V	0.20	−0.11	−0.23	−0.31
*E*_1/2_ (2nd ox.)/V	0.67	0.18	—	—
*E*_1/2_ (red.)/V	−1.44	−1.86	−2.18	−2.27

**Fig. 4:**
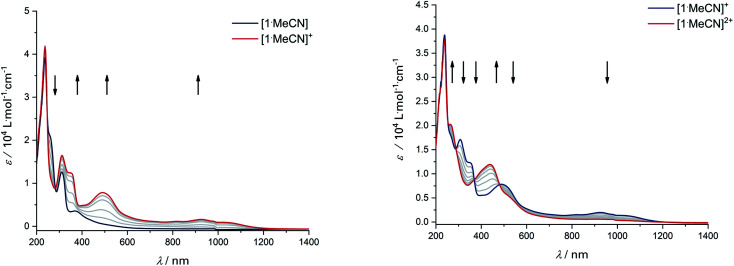
UV/Vis-SEC of complex [1·MeCN] in MeCN/*n*-Bu_4_PF_6_ at 293 K. Left: Spectral changes during first oxidation. Right: Spectral changes during second oxidation.

**Fig. 5:**
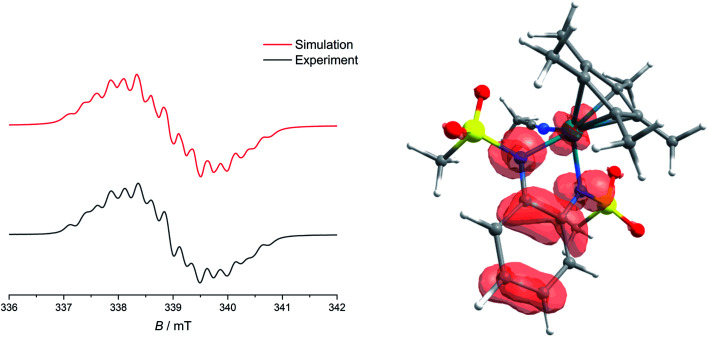
Left: Experimental and simulated EPR spectra of [1^.^MeCN]^+^, obtained from electrolysis in MeCN/*n*-Bu_4_PF_6_ at 293 K. Right: Spin-density of [1^.^MeCN]^+^, calculated at the TPSSh/def2-TZVP level of theory.

Upon the first oxidation, broad, long wavelength bands appear in the spectrum of [1·MeCN]^+^, concomitant with the appearance of a band at around 500 nm (Fig. 4, left). These features are typical for metal-bound radical ligands.^63,64^ The radical nature of [1·MeCN]^+^ was unequivocally shown by EPR-SEC (Fig. 5, left): oxidation leads to a line-rich spectrum centered around *g* = 1.99, which can be simulated by taking into account hyperfine coupling to two equivalent N nuclei (*A*_N1_ = 14.1 MHz), an additional N nucleus (*A*_N2_ = 9.4 MHz), the ^103^Rh nucleus (*A*_Rh_ = 20.1 MHz) and two sets of two equivalent protons (*A*_H1_ = 12.4 MHz, *A*_H2_ = 7 MHz).

This is in line with an unpaired electron delocalized over the chelating CNNC moiety, with additional contribution from the metal center and the aromatic backbone to the SOMO. DFT calculations reproduce these findings (Fig. 5, right). No signs of decomposition were observed for [1·MeCN]^+^ on the spectroelectrochemical timescale (*i.e.* several minutes). Removal of a second electron leads to a disappearance of the bands in the NIR region and new bands between 400 and 600 nm (Fig. 4, right). Such spectral signatures indicate MLCT and LL’CT transitions to an iminoquinonoid ligand;^57^ however, within seconds the doubly oxidized complex begins to decompose.

**Fig. 6:**
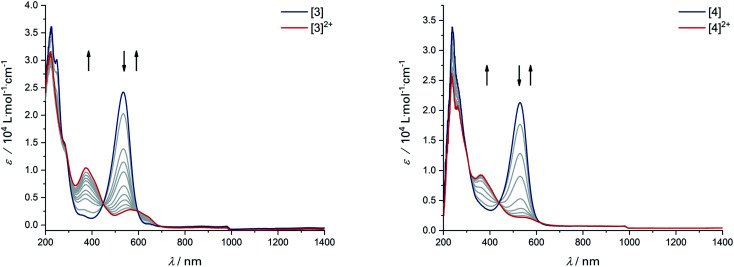
UV/Vis-SEC of complexes [3] and [4] in MeCN/*n*-Bu_4_PF_6_ at 293 K. Left: Spectral changes during two-electron oxidation of [3]. Right: Spectral changes during two-electron oxidation of [4].

**Fig. 7:**
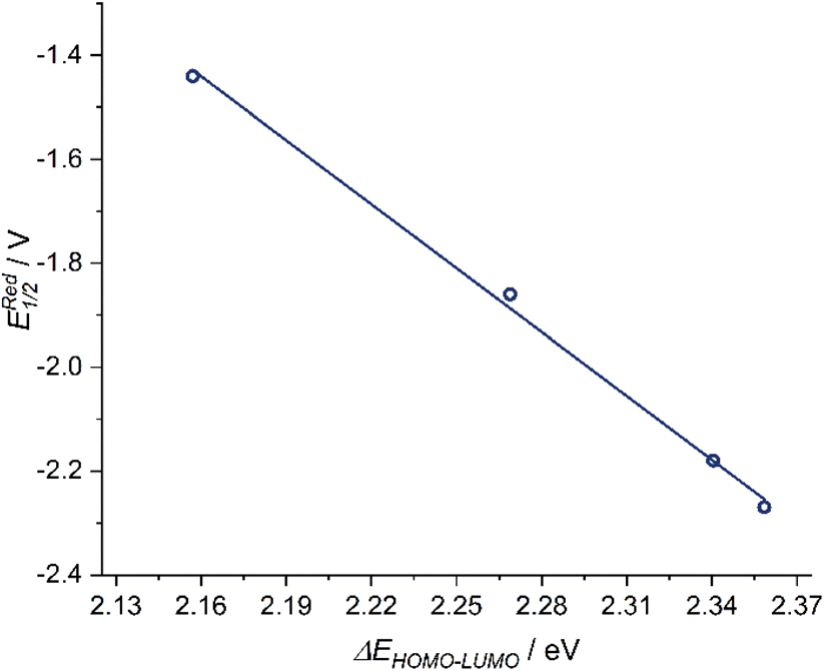
Left: Correlation of *E*^Reduction^_1/2_ and Δ*E*_HOMO–LUMO_ with a goodness-of-fit *R*^2^ = 0.997.

The donor strength of an imine with strongly electron-withdrawing substituents is probably so low that dissociation of the oxidized ligand occurs. The two-electron oxidations of [3] and [4] directly lead to spectral features that indicate an *o*-iminoquinonoid ligand structure (Fig. 6), but in these cases, the oxidized species are stable on the spectroelectrochemical timescale. The electrochemical and spectroscopic results indicate that modifications on the [N–R] handle have a strong influence on the potential and separation of oxidation events in diamidobenzene ligands. By comparing structurally related systems, these results point to a design strategy for ligands that may serve as either one- or two-electron reservoirs: the electron-poor sulfonamide moiety seems to preferentially stabilize ligand-centered radicals, while the [N–H] group appears to induce follow-up reactions of such radicals. This is likely due to radical H atom abstraction from the NH handle after single-electron oxidation. In contrast, both [N–H] and [N–Ph] substituents support the concerted two-electron oxidation to a stable iminoquinoid ligand. Ligand-centered one- and two-electron oxidations have been described previously for analogous systems;^9,56,57^ however, the reversible reductions at potentials between −1.44 V ([1]) and −2.27 V ([4]) against FcH/FcH^+^ are a novel feature. The cathodic shifts of the potentials follow the trend in donor strength described above and show a linear correlation with the calculated HOMO–LUMO gaps of complexes [1]–[4] (see Fig. 7), which indicates a similar electronic structure in the reduced species and suggests the possibility of predictively designing complexes with a desired reduction potential. At the TPSSh/def2-TZVP level of theory, the correlation is described by the following formula:*E*^Red^_1/2_ [V] = 7.43 V − 4.10 × Δ*E*_HOMO_–_LUMO_ [eV]

**Fig. 8:**
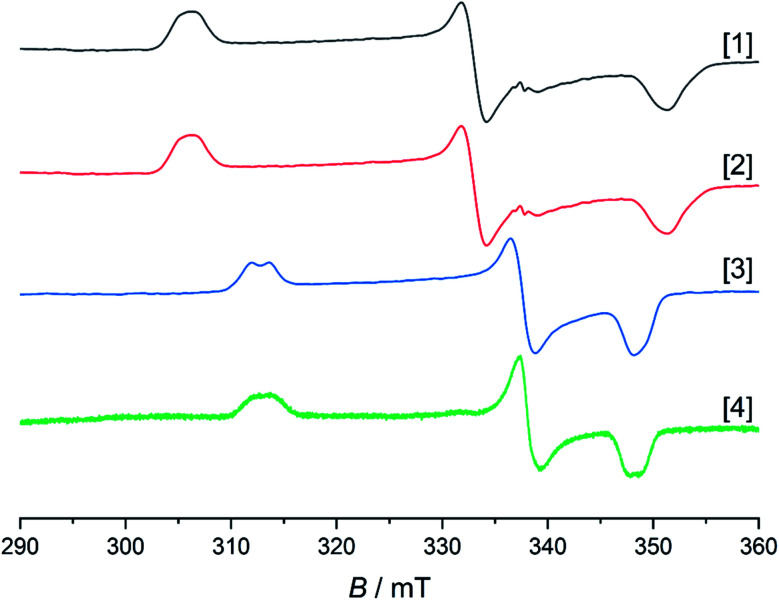
EPR spectra of electrochemically generated [1]^−^, [2]^−^, [4]^−^ in frozen MeCN and chemically reduced [3]^−^ in frozen THF. Spectra recorded at 103 K. Intensities are normalized. Due to lower concentration in the SEC experiment, the signal to noise ratio is also lower for [4]^−^.

As mentioned above, all compounds show qualitatively similar LUMO shapes with a sizable contribution of a metal-centered orbital, which points to a reduction to formally Rh^ii^. EPR-SEC studies verified the assumption of metal-centered processes: the (electro-)chemically reduced species show rhombic, anisotropic spectra indicative of metalloradicals (Fig. 8 and S17†). The *g*-values are in line with previously described Rh^ii^ complexes^65,66^ and particularly similar to a [Cp*Rh^ii^] moiety bound to a doubly reduced azobispyridine system.^59^ In case of electrogenerated [3]^−^ and [4]^−^, hyperfine coupling to the ^103^Rh nucleus is partially resolved in *g*_1_ and *g*_3_. UV/Vis-SEC measurements confirm the reversibility of the processes and the stability of the reduced species in solution (Fig. S21–S24†). Unrestricted DFT calculations on the reduced species show a major contribution (55–64%) of Rh to the spin population (Fig. S29†). The calculated *g*-values are in qualitative agreement with the experimental values (see Table 3).

**Table 3:** Experimental *g*-values and calculated values at the UKS-TPSSh/def2-TZVP level of theory

	[1]^−^	[2]^−^	[3]^−^	[4]^−^
*g*-Values exp., (calc.)	1.92, (1.94)	1.93, (1.96)	1.94, (1.96)	1.94, (1.96)
	2.03, (2.03)	2.03, (2.02)	2.00, (2.00)	2.00, (2.00)
	2.21, (2.16)	2.20, (2.13)	2.16, (2.12)	2.16, (2.12)
Spin population Rh (Loewdin)	64%	59%	55%	55%

The EPR- and UV/Vis-SEC results clearly indicate that the reduction of the neutral Rh(iii) complexes leads to the formation of stable, mononuclear, anionic Rh(ii) compounds. As is apparent from Fig. 4 and Table 3, the *g*-anisotropy decreases with increasing donor strength. This trend can be qualitatively rationalized by inspecting the relevant orbitals as calculated on the TPSSh/def2-TZVP level of theory: the SOMO corresponds to the LUMO of the neutral complexes (Fig. 2) and shows π* symmetry regarding the metal diamide interaction with a large contribution from the metal d_*yz*_ orbital. Stronger π donors will engender a larger energy splitting between the bonding and antibonding π orbital, effectively pushing up the SOMO in energy. In a first approximation, the deviation from *g*_el_ = 2.0023 depends inversely on the energy difference between the ground state and excited states, in which electrons are promoted from the doubly occupied 4d orbitals into the SOMO. Energetically pushing up the SOMO thus increases this energy difference and consequently reduces the *g* anisotropy. An additional factor is the increasing covalency of the metal–ligand bonds, which is also reflected in the decrease of Rh-centered spin population (Table 3).

**Fig. 9:**
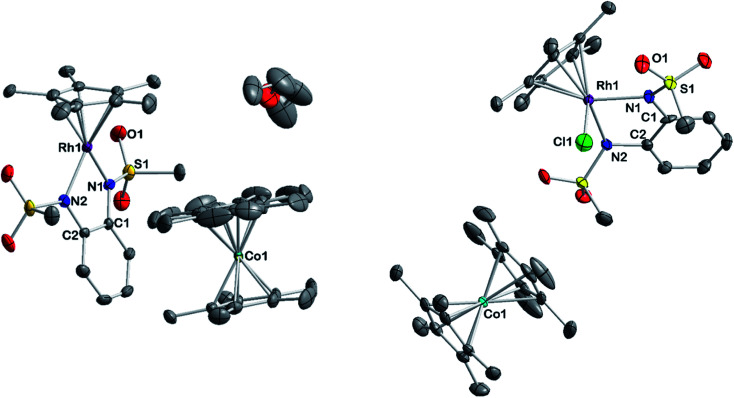
Molecular structure of reduced complex [CoCp*_2_][1] (left) and one product of its reaction with DCM [CoCp*_2_][1-Cl] (right). Ellipsoids drawn at 50% probability. H atoms omitted for clarity.

**Scheme 3:**
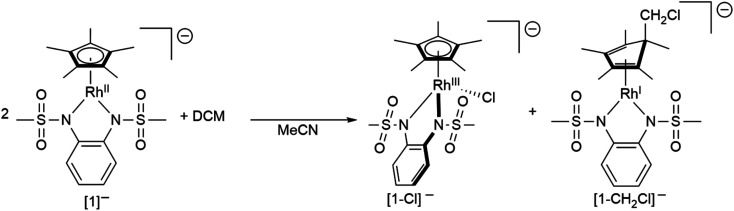
The reaction of [1]^−^ with DCM.

To gain insight into the structure and reactivity of the reduced species, we conducted a preliminary case study on [1]^−^ as a representative example. A symmetrically substituted species was chosen as we expected this to be advantageous regarding both crystallization and data analysis in reactivity studies. Treatment of [1^.^MeCN] with [CoCp*_2_] in acetonitrile gave a very air-sensitive green solution, from which [CoCp*_2_][1] was isolated as crystalline material upon layering with diethyl ether and storage at −30 °C. [CoCp*_2_][1] crystallizes in the space group *P*2_1_ with one molecule of diethyl ether. An inspection of the molecular structure given in Fig. 9 on the left, shows that upon reduction, the bound acetonitrile dissociates and [1]^−^ adopts a coordinatively unsaturated geometry due to the increased electron density at the central metal. This leaves a free coordination site open for further reactivity. In comparison with the neutral species, the Rh–N bond lengths are elongated by ∼0.05 Å. Characteristic bond lengths and angles are given in Table S11.† Initial reactivity studies attest to the metalloradical behavior of [1]^−^: EPR spectra show the formation of a thermally unstable rhodium superoxide upon contact with O_2_ (Fig. S26†). The formation of superoxido species has been previously described for other Rh^II^ complexes.^67–69^ We also investigated the reactivity of [1]^−^ towards the geminal dihalocarbon dichloromethane, as *d*^7^ metalloradicals are known to activate RX bonds.^70^ Indeed, upon treating an acetonitrile solution of [1]^−^ with dichloromethane, more than 70% conversion is observed within 4 h (yields spectroscopically determined). Two products are formed, which can be identified by their ^1^H- and ^13^C-NMR spectra along with mass spectrometry. The homolytic splitting of the C–Cl bond leads to the formation of an anionic Rh^III^ chloride complex and subsequently to the addition of the ·CH_2_Cl fragment to the Cp* unit in a second molecule of [1]^−^ (see Scheme 3). A detailed interpretation of the spectra is given in the ESI.† The net reaction in Scheme 3 presents a disproportionation of two Rh^II^ molecules into a Rh^III^ and a Rh^I^ complex – such reactions have been previously described for different Rh^II^ species.^67,71,72^ The reactivity towards DCM is very similar to the ‘RX’ reaction originally described by Wilkinson^73^ and later Kölle^74^ for cobaltocene and decamethylcobaltocene, respectively. While we haven't been able to quantitatively separate the two reaction products so far, we succeeded in crystallizing the chlorido adduct. The molecular structure is shown in Fig. 9 on the right. These results show that the metalloradical reactivity of decamethylcobaltocene is transferred onto the Rh^II^ metalloradical [1]^−^. Considering the substantially more negative potentials in [2]^−^, [3]^−^ and [4]^−^, the activation of very stable carbon-halogen bonds seems likely. Additional reactivity studies towards that end are currently being undertaken in our laboratories.

**Scheme 4:**
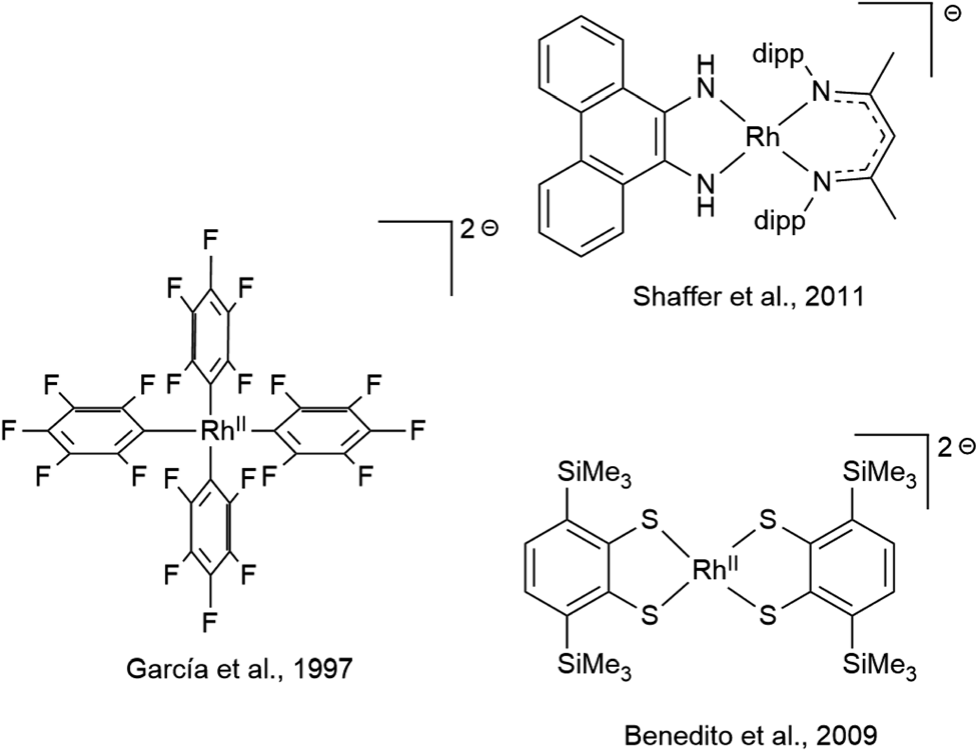
Previously reported and structurally characterized anionic Rh^ii^ complexes.

While mononuclear Rh^ii^ complexes are still rather uncommon and often rely on sophisticated ligand design,^75–78^ anionic Rh^ii^ complexes are particularly rare.^79^ Only very few have been structurally characterized, three of which are shown in Scheme 4.^80–82^ Rh^II^ complexes have been shown, *inter alia*, to activate C–C^83^ and C–H^84^ bonds as well as dihydrogen^85,86^ and to engage in H atom transfer.^87^ The reactivity of Rh^ii^-ate complexes however has been scarcely explored, even though they were recently proposed as intermediates in the photo-induced ortho-C–H borylation of arenes.^88^ The diamidobenzene scaffold allows access to a new class of electron-rich Rh^ii^-ate complexes with tunable potential. Note that the complexes [1] to [4] span a potential range of 830 mV regarding the Rh^iii^/Rh^ii^ couple. Considering the plethora of additional substitution patterns of diamidobenzene accessible *via* facile functionalization of either *o*-dibromobenzene or *o*-phenylenediamine, this potential range could be extended to over 1 V. The linear correlation of the reduction potential with the HOMO–LUMO gap possibly allows a predictive design strategy. Therefore, diamidobenzene complexes of [Cp*Rh] constitute a promising compound class to study the possibilities of Rh^ii^-ate complexes in chemical transformations.

**Scheme 5:**
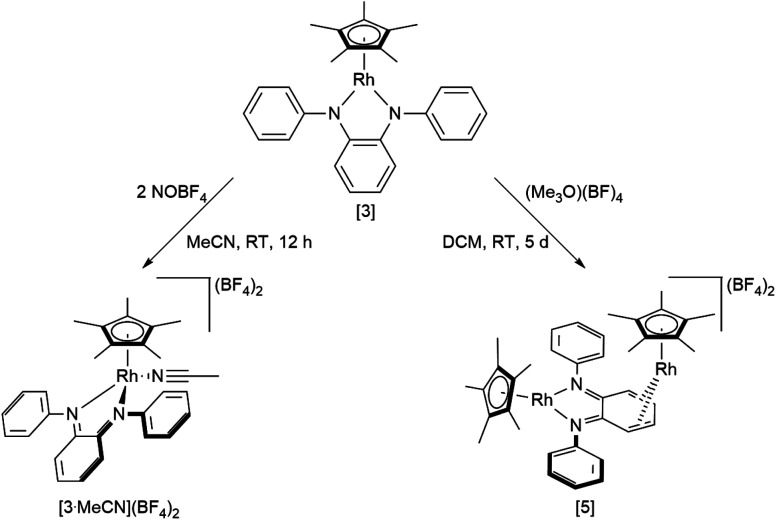
Reactivity of [3] towards oxidizing substrates NOBF_4_ and Meerwein's salt.

**Fig. 10:**
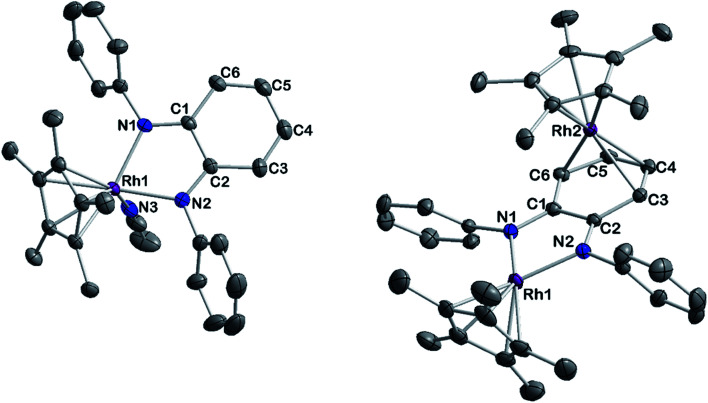
ORTEP representation of the molecular structures of [3^.^MeCN]^2+^ (left) and [5]^2+^ (right). Ellipsoids drawn at 50% probability. H atoms, anions and solvent molecules have been omitted for clarity.

An investigation into the oxidative reactivity (Scheme 5) offered novel insights as well. As a case study, we focused on the symmetrically substituted species [3]. Isolation and crystallographic characterization (Fig. 10, left) of the chemically oxidized complex [3^.^MeCN]^2+^ confirmed the ligand-centered oxidation: C=N bond lengths of 1.301(3) Å, a contraction of the C3–C4 and C5–C6 bond distances to around 1.34 Å together with an elongation of the residual C–C bonds in the backbone are all in line with an *o*-iminoquinonoid structure. The reduced donor strength of the oxidized ligand leads to additional coordination of a solvent molecule, similar to structures observed in other Rh^iii^ diimine complexes.^89^ The dianionic ligand in [3] can therefore act as an electron reservoir, analogous to the diamidobenzene ligand which enables the oxidative addition of [CH_3_]^+^ groups to an Ir^iii^ center.^56^ Thus, we treated symmetrical, phenyl-substituted complex [3] with one equivalent of Meerwein's salt (Me_3_O)(BF_4_) to see whether a similar reactivity would be observed (Scheme 5).

However, upon work-up and crystallization a completely different product was obtained: we isolated the dicationic, dinuclear complex [5](BF_4_)_2_ in 46% yield, in which a second [Cp*Rh] moiety is coordinated to the backbone of the *o*-diiminoquinone (Fig. 10, right). ^1^H- and ^13^C-NMR-spectra as well as ESI mass spectra confirm that the same constitution is present in solution: characteristic doublet splittings of the ^13^C resonances in the ligand backbone are proof of bonding to the ^103^Rh nucleus. Similar structures were described by Amouri and co-workers for *o*-benzoquinones in 2009:^90^ they developed a synthetic access to π-bonded rhodio-*o*-benzoquinones, in which a [Cp*Rh^i^] fragment is coordinated in *η*^4^-fashion to the ligand backbone. These compounds were also shown to act as organometallic linkers *via* the chelating benzoquinone moiety (Scheme 5).^65^

The unexpected formation of a heterovalent, dinuclear rhodium complex with a certain amount of redox ambiguity is reminiscent of results described by Tejel, de Bruin and co-workers in 2008.^91^ They used a combination of structural and NMR data to describe the valency of two distinct Rh sites in a dinuclear species. Following that approach, the ^1^*J*_Rh,C_ couplings in [5](BF_4_)_2_ offer a first hint at an appropriate description of the bonding and valency in this compound: only C3/C6 and C4/C5 show coupling to a Rh nucleus (Fig. S12†). This is in line with shorter bond lengths between these carbon atoms and Rh2. A side-view of the molecular structure (Fig. S36†) shows a loss of planarity (and hence, aromaticity) in the ligand backbone. Both the spectral and structural features are comparable to the results obtained by Amouri and co-workers.^65,90^ Taken together, these findings imply the presence of a neutral iminoquinone ligand, which binds to a Rh^i^ moiety through its diolefinic backbone. Both the Rh–C bond distances of around 2.2 Å and the C=C bond lengths of around 1.4 Å correspond to reported values for diolefins coordinated to Rh^i^.^92,93^ The bond lengths between C3–C4 and C5–C6 are elongated by approximately 0.06 Å compared to the iminoquinonoid structure in [3^.^MeCN]^2+^ (Table 4). While the group of Amouri has shown that π-bonded metallo-*o*-benzoquinones and the heavier S- and Se-congeners may serve as organometallic ligands, our results extend this concept to *o*-iminoquinones, and this to the best of our knowledge is the first example of such a structural motif with *o*-diiminoquinones. Considering the possible photochemical applications of such ligand systems, an extension to N-donor analogues offers interesting new possibilities. Despite its structural similarity to previously reported complexes mentioned above, the mechanism of the formation of [5](BF_4_)_2_ is fundamentally different. We followed the reaction by ^1^H-NMR spectroscopy (Fig. S33†) and were able to identify the initial formation of the methylated Rh complex [3-CH_3_]^+^ by the appearance of a doublet in the high-field region (*δ* = 0.75 ppm, ^2^*J*_Rh,H_ = 2.4 Hz), which shows a chemical shift and coupling constant in the range of previously reported Rh–CH_3_ groups.^94–96^ A weak resonance at *δ* = 3.02 ppm may be tentatively assigned to chloromethane.^97^ This might indicate that the metal-bound methyl group abstracts chloride from the solvent. The related Ir–CH_3_ complex we reported on was stable in DCM but showed reactivity towards haloforms.^56^ In light of the frequently observed higher reactivity of Rh species, it seems plausible that the Rh–CH_3_ complex is reactive towards DCM. Additionally, signals at *δ* = 3.20 ppm and *δ* = 6.26 ppm in a 3 : 1 ratio are similar to resonances described for *N*,*N*′-diphenyl-*N*-methylphenylenediamine.^98^ Hence, a methylation of the diamidobenzene ligand cannot be ruled out as a possible mechanistic step. These findings show an important difference between the isoelectronic complexes [3] and [Cp*Rh(bpy)]: the group of Blakemore isolated and characterized the stable methylated half-sandwich compound [Cp*Rh(bpy)Me] in 2017;^96^ in contrast, the diolefinic backbone in our system appears to favor the rearrangement to a dinuclear complex. While the isolation of [3-CH_3_]^+^ from solvents other than DCM has so far been unsuccessful, its preparation and the transformation into the homobimetallic, heterovalent, complex [5](BF_4_)_2_ are matters of on-going research in our group. Whether this reactivity can also be extended to differently substituted diamidobenzenes is also currently under investigation.

**Table 4:** Bond lengths in complex [3]^2+^ and [5]^2+^. All values given in Å

	[3]^2+^	[5]^2+^
Rh–N1/N2	2.088(2)/2.099(2)	2.018(3)/2.012(3)
N1–C1/N2–C2	1.301(2)/1.301(2)	1.348(4)/1.339(4)
C1–C2	1.476(3)	1.451(4)
C2–C3/C1–C6	1.411(2)/1.411(2)	1.426(4)/1.418(4)
C3–C4/C5–C6	1.346(3)/1.343(2)	1.400(5)/1.410(4)
C4–C5	1.437(2)	1.412(5)
Rh2–C4/C5	—	2.183(3)/2.200(3)
Rh2–C3/C6	—	2.232(3)/2.232(3)

## Conclusion

We have shown the synthesis and comprehensive (spectro-)elecotrchemical characterization of four different diamidobenzene complexes of Rh^iii^. The substituents on the N-atoms of the diamidobenzene ligands have a very strong influence on the bonding, donor, electrochemical and spectroscopic properties of the Rh complexes. Depending on the substitution pattern, the redox-active ligands display very different donor properties and prefer either one- or two-electron oxidations. All complexes can be reversibly reduced to electron-rich, mononuclear Rh^ii^ metalates at potentials that correlate with the electron-releasing character of the ligand. To the best of our knowledge, this is the first time that isolable mononuclear Rh^ii^ metallates with such extreme tuning of redox potentials have been reported. An isolated example was shown to undergo typical metalloradical reactivity such as O_2_ activation and C–Cl bond homolysis. Furthermore, oxidation-mediated dinucleation *via* a transient Rh–CH_3_ species allowed the first characterization of a π-bonded rhodio-*o*-diiminoquinone. Such structures are completely unprecedented in the chemistry of metal complexes of *o*-diiminoquinones. In summary, our research confirms the potential of diamidobenzene complexes of Rh(iii) in organometallic chemistry, as they combine salient features of metal- and ligand-centered reactivity in one molecular entity. As we have shown, such features can lead to the generation of completely unexpected properties, as well as the generation of totally unprecedented structures.

## Data availability

The data that support the findings of this study are available in the ESI† of this article.

## Author contributions

SS and BS designed the project. SS carried out all the synthesis, characterization, reactivity studies, spectroscopic, electrochemical and spectroelectrochemical measurements, and DFT calculations. RW, JB and UA were responsible for solving the crystal structures. SS and BS jointly wrote the manuscript with help from the other authors.

## Conflicts of interest

There are no conflicts to declare.

## Supplementary Material

SC-013-D2SC03227A-s001

SC-013-D2SC03227A-s002
