# Supplementary material for: Rhodium diamidobenzene complexes: a tale of different substituents on the diamidobenzene ligand
Source: Chem Sci. 2022 Jul 21;13(35):10532–45. doi: 10.1039/d2sc03227a (PMC9473529; doi:10.1039/d2sc03227a)
Supplement: SC-013-D2SC03227A-s001 [file SC-013-D2SC03227A-s001.pdf]

Electronic Supplementary Material (ESI) for Chemical Science.
This journal is © The Royal Society of Chemistry 2022

Rhodium Diamidobenzene Complexes: A Tale of Different Substituents on the Diamidobenzene Ligand

Simon Suhr,[a] Robert Walter,[a] Julia Beerhues,[a] Uta Albold,[b] Biprajit Sarkar[a*]

a) Lehrstühl für Anorganische Koordinationschemie, Institut für Anorganische Chemie, Universität Stuttgart, Pfaffenwaldring 55, 70569 Stuttgart

b) Institut für Chemie und Biochemie, Freie Universität Berlin. Fabeckstr. 34-36, 14195 Berlin

Contents

Instrumentation 1
Synthetic Details 3
NMR Spectra 6
Electrochemistry 12
EPR Spectroscopy/Spectroelectrochemistry 16
UV/Vis-NIR-Spectroscopy/Spectroelectrochemistry 17
Reactivity studies 21
DFT calculations 28
Crystallographic Details 47
References 53

Instrumentation

General information: Unless otherwise stated, all manipulations were performed under Argon (Air Liquide, ALPHAGAZ™, purity ≥99.999 % using standard Schlenk techniques or inside an Ar filled glovebox (MEGA by GS Glovebox Systemtechnik). NMR spectra were recorded on Jeol ECS/ECZ 400/400R or Bruker AvanceIII HD 700 spectrometers. Signals were referenced to residual solvent shift[1] and are given in parts per million (ppm) relative to the TMS signal. Multiplets are reported as follows: singlet (s), duplet (d), triplet (t), quartet (q), quintet (quint), septet (sept), and combinations thereof. Mass spectra were recorded on microTOFQ Bruker Daltonics. Elemental analyses were performed using a Elementar VarioMICRO cube.

Single-Crystal X-Ray diffraction: Single crystal X-ray diffraction was performed on a Bruker Kappa ApexII Duo diffractometer at 140(2) K or a Bruker D8 Venture system at 100(2) K using graphite- or mirror-monochromated Mo Kα radiation (λ_α = 0.71073 Å). The strategy for the data collection was evaluated by using the APEX2 software. The data were collected by ω- and φ-scan techniques and scaled and reduced using the APEX2 and SADABS software. The structures were solved by intrinsic phasing using SHELXT-2015[2] and refined using SHELXL

by full-matrix least squares, refining on F^2, using ShelXLe.[3,4] Non-hydrogen atoms were refined anisotropically, and H atoms were included using a riding model.

Electrochemistry: Cyclic voltammetry was performed with a PalmSens4 potentiostat using a three-electrode set-up. A glassy carbon disk electrode (3 mm) was used as working electrode, coiled Pt wire as counter electrode and Ag wire as pseudo-reference. Solvents were freshly distilled and degassed before each measurement. n-Bu_4PF_6 (Sigma-Aldrich) was used as supporting electrolyte. After completing the measurement, either Ferrocene or Decamethylferrocene ($E_{1/2} = -510$ mV vs. FcH/FcH^+ in MeCN)[5] were added as internal references.

UV/Vis-NIR Spectroscopy: UV/Vis-NIR measurements were performed on a J&M Tidas UV-Vis-NIR spectrophotometer. Spectroelectrochemical measurements were performed in an optically transparent thin-layer electrochemical (OTTLE) cell[6] (CaF_2 windows) with either a platinum-mesh or a gold-mesh working electrode, a platinum-mesh counter electrode, and a silver-foil pseudoreference electrode. An Autolab PGSTAT101 potentiostat (Metrohm) was used for all spectro-electrochemical measurements.

EPR Spectroscopy: EPR spectra at X-band frequency (ca. 9.5 GHz) were obtained with a Magnettech MS-5000 benchtop EPR spectrometer equipped with a rectangular TE 102 cavity and TC HO4 temperature controller. The measurements were performed in synthetic quartz glass tubes. For EPR spectro-electrochemistry a three-electrode setup was employed using two Teflon-coated platinum wires as working and counter electrodes and a Teflon-coated silver wire as pseudo-reference electrode. Spectral simulations were performed with EasySpin 5.1.4[7] and MatLab R2012a.

Computational Details: All calculations were performed using the ORCA software package, program version 4.2.1.[8,9] Geometry optimizations were carried out using the BP86 functional and a def2-TZVP basis set.[10] The optimized geometries were verified to be minima by the absence of imaginary frequencies in numerical frequency calculations. Single-point calculations were performed using the TPSSh[11] functional and a def2-TZVP basis set using the unrestricted Kohn-Sham formalism. Relativistic effects were included by the zeroth order approximation (ZORA). Dispersion was modeled by using Grimme's D3 correction.[12] The resolution of identity (RI) approximation[13–16] was employed using suitable auxiliary basis sets.[17–19] Visualization of orbitals and densities was done using CHEMCRAFT visualization software.

Synthetic Details

General information: Unless otherwise stated, all syntheses were performed under Argon (Air Liquide, ALPHAGAZ™, purity ≥99.999 %, additionally dried over a P_2O_5 and orange gel column) using standard Schlenk techniques. [Cp*$RhCl_2$] and ligands H_2bmsab[20], H_2amsab[21] and H_2bphab[22] were synthesized by literature procedures. H_2aphab was obtained from abcr and used without further purification. [CoCp*$_2$] was obtained from Sigma-Aldrich and re-sublimated before use. NEt_3 was distilled, stored over molecular sieves and degassed by bubbling with Ar for at least 30 min. Dry solvents were collected from solvent purification systems (GS GLOVEBOX or Innovative Technology PURESOLV), stored over activated molecular sieves and degassed by bubbling with Ar for at least 30 min.

General procedure for the synthesis of complexes [Cp*RhL]: [Cp*$RhCl_2$]$_2$ (0.5 mmol, 309 mg, 1 eq.) and ligand H_2L (1 mmol, 2 eq.) are suspended in 20 ml of DCM and NEt_3 (10 mmol, 1.012 g, 1.39 ml) is added dropwise, resulting in a color change from red to dark purple After stirring for 48 h, work-up is performed under ambient conditions. The reaction mixture is washed with demineralized water three times; subsequently, the organic phase is dried over Na_2SO_4, filtered and the solvent removed. The complexes are either directly obtained in pure form after work-up, purified by extraction with diethyl ether or re-crystallized from appropriate solvents.

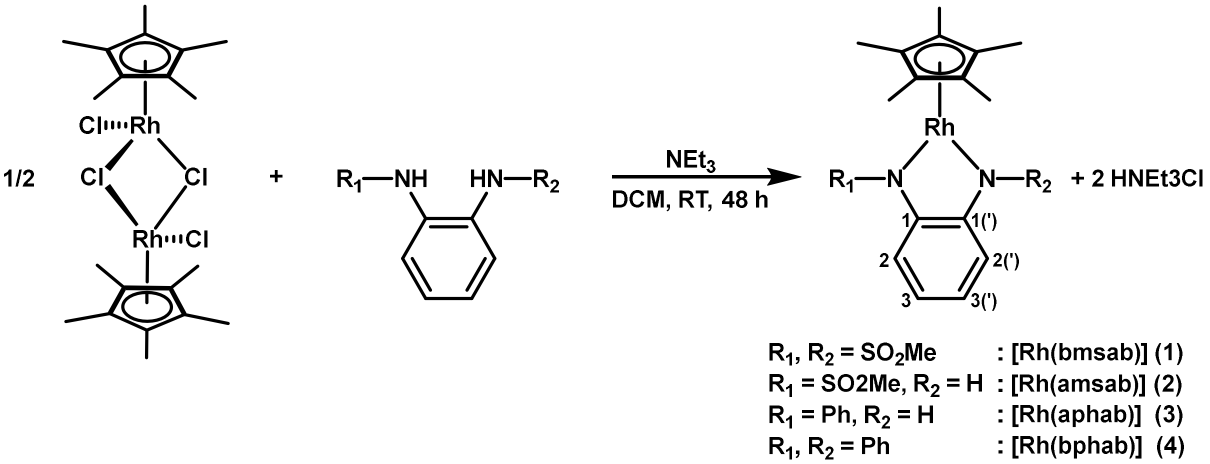

Scheme S1: General synthetic procedure for the synthesis of Rh complexes.

[Cp*Rh(bmsab)] ([1]): Obtained as an analytically pure, dark purple, microcrystalline powder after work-up. Yield: 80 %. Single crystals were grown by layering a concentrated DCM solution with ethanol.

^{1}H-NMR (400 MHz, 25 °C, CD_2Cl_2): δ = 7.57 (m, 2 H, H^2), 6.81 (m, 2H, H^3), 3.02 (s, 6 H, $SO_2C\mathit{H}_3$), 1.81 (s, 15 H, Cp*-$C\mathit{H}_3$) ppm.

^{13}C{1H} NMR (101 MHz, 25 °C, CD_2Cl_2): δ = 142.4, 1221.0, 117.2, 98.9 (d, $^1J_{Rh,C}$ = 8.5 Hz, Cp*-*C*), 40.4 (s, $SO_2\mathit{C}H_3$), 11.4 (s, Cp*$\mathit{C}H_3$) ppm.

HR-MS (ESI): *m/z* = 523.0195 [M + Na^+], calcd. for [M + Na^+] $C_{18}H_{25}N_2O_4RhS_2Na^+$: *m/z* = 523.0209.

Anal. calcd. for $C_{18}H_{25}N_2O_4RhS_2Na$: C 43.20, H 5.04, N 5.60, S 12.81. Found: C 42.93, H 5.11, N 5.30, S 12.88.

[Cp*Rh(amsab)] ([2]): Obtained in pure form after crystallization from DCM/hexane as a dark purple, microcrystalline powder. Yield: 36 %. Single crystals were grown by layering a concentrated DCM solution with n-hexane.
^{1}H-NMR (400 MHz, 25 °C, CD_2Cl_2): δ = 8.18 (br s, 1 H, N*H*), 7.70 (m, 1 H, Ph), 6.84 (m, 2 H, Ph), 6.76 (m, 1 H, Ph), 3.04 (s, 3 H, SO_2CH_3), 1.81 (s, 15 H, Cp*C*H*3) ppm.
^{13}C{1H} NMR (101 MHz, 25 °C, CD_2Cl_2): δ = 152.4 (Ph), 139.2 (Ph), 120.7 (Ph), 118.3 (Ph), 117.0 (Ph), 114.2 (Ph), 94.7 (d, Cp*, $^1J_{Rh,C}$ = 7.4 Hz), 39.8 (SO_2CH_3), 10.6 (Cp**C*H3) ppm.
HR-MS (ESI): *m/z* = 445.0408 [M + Na^+], calcd. for [M + Na^+] $C_{17}H_{23}RhN_2O_2SNa^+$: *m/z* = 445.0433.

[Cp*Rh(bphab)] ([3]): Obtained as an analytically pure, dark red, microcrystalline powder after extraction with diethyl ether. Yield: 75 %. Single crystals were grown by slow evaporation from a concentrated DCM solution.
^{1}H-NMR (400 MHz, CD_2Cl_2, 25 °C): δ = 7.51 (m, 4 H, *m*-Ph H), 7.30 (m, 2 H, *p*-Ph H), 7.24 (m, 4 H, *o*-Ph), 6.62 (m, 2 H, H1, H1'), 6.53 (m, 2 H, H2, H2'), 1.39 (s, 15 H, Cp* H) ppm.
^{13}C{^{1}H}-NMR (101 MHz, CD_2Cl_2, 25 °C): δ = 155.13 (s, i-Ph *C*), 148.65 (s,), 128.78 (s, m-Ph *C*), 126.08 (s, o-Ph *C*), 124.52 (s, p-Ph *C*), 117.07 (s, *C1*), 111.08 (s, *C2*), 91.73 (d, *J* = 7.65 Hz, Cp*-*C*), 8.66 (s, Cp*-CH_3) ppm.
HR-MS (ESI): *m/z* = 497.1457 [M + H^+], calcd. for [M + H^+] $C_{28}H_{30}N_2Rh^+$: *m/z* = 497.1464.
Anal. Calcd. for $C_{28}H_{29}N_2Rh$: C 67.74, H 5.89, N 5.64. Found: C 67.85, H 6.07, N 5.77.

[Cp*Rh(aphab)] ([4]): Obtained as an analytically pure, dark red, microcrystalline powder after extraction with diethyl ether. Yield: 80 %. Single crystals were grown by slowly cooling a saturated pentane solution to −30 °C.
^{1}H-NMR (400 MHz, 25 °C, CD_2Cl_2): δ = 8.31 (br s, 1 H, N*H*), 7.46 (m, 2 H, Ar *H*), 7.25 (m, 1 H, Ar *H*), 7.16 (m, 2 H, Ar *H*), 6.92 (m, 2 H,), 6.71 (m, 1H,), 6.51 (m, 2 H,), 1.75 (s, 15 H, Cp*CH_3) ppm.
^{13}C{1H}-NMR (101 MHz, 25 °C, CD_2Cl_2): δ = 155.9, 149.9, 148.7, 129.3, 126.3, 124.9, 118.3, 117.0, 114.3, 111.9, 91.9 (d, $^1J_{Rh,C}$ = 7.6 Hz, Cp*), 10.1 (s, Cp*CH_3) ppm.
HR-MS (ESI): *m/z* = 421.1138 [M + H^+], calcd. for [M + H^+] $C_{22}H_{27}N_2Rh^+$: *m/z* = 421.1151.
Anal. Calcd. for $C_{22}H_{25}N_2Rh$: C 62.86, H 5.99, N 6.66. Found: C 61.86, H 6.305, N 6.22.

[CoCp*$_2$][Cp*Rh(bmsab)]: A Schlenk tube is charged with **[1]** (50 mg, 0.1 mmol, 1 eq) and [CoCp*$_2$] (33 mg, 0.1 mmol, 1 eq). Acetonitrile (3 ml) is added under strict exclusion of air and moisture. The reaction mixture is stirred for 3 h to give an extremely air-sensitive green solution. Layering with diethyl ether (20 ml) and storage at −30 °C for at least one week yields 40 mg of block-shaped, dark green crystals suitable for X-ray diffraction in 50 % yield. While isolation on a Schlenk line is possible, we recommend using a glovebox for work-up due to the pronounced air-sensitivity of the solution.
Anal. Calcd. for $C_{38}H_{55}CoN_2O_4RhS_2$: C 55.0, H 6.68, N 3.38. Found: C 54.83, H 6.79, N 3.46.

[Cp*Rh(bphib)MeCN](BF_4)$_2$: A Schlenk flask is charged with **[3]** () and $NOBF_4$. Acetonitrile is added and the reaction is stirred for 10 min; subsequently, the flask is quickly opened to vacuum to remove NO gas and then flushed with Argon. This procedure is repeated three times, after which the reaction mixture is stirred for 12 h. The solvent is removed and the dark solid dried under vacuum for 2 h. Subsequently, the crude solid is dissolved in acetonitrile and crystallized by vapor diffusion of diethyl ether to give dark green single crystals of the pure product. The product is hygroscopic.
^{1}H-NMR (700 MHz, CD_2Cl_2, 25 °C): δ = 7.73 (br m, 4 H, *m*-Ph H), 7.63 (tt, $^3J_{H,H}$ = 7.4 Hz, $^5J_{H,H}$ = 1.4 Hz, 2 H, p-Ph H), 7.35 (d, $^3J_{H,H}$ = 7.4 Hz, 4 H, o-Ph H), 7.01 (m, 2 H,), 6.78 (m, 2H,), 1.18 (s, 15 H, Cp*CH_3) ppm.
^{13}C{^{1}H}-NMR (176 MHz, CD_2Cl_2, 25 °C): δ = 169.1, 146.1, 138.5, 130.4, 122.7, 103.7 (d, $^1J_{Rh,H}$ = 7.75 Hz), 8.5 (s, Cp*CH$_3$) ppm.
Anal. Calcd. for $C_{30}H_{32}B_2F_8N_3Rh$ 0.5 CH_3CN · 0.45 H_2O: C 50.33, H 4.69, N 6.63. Found: C 50.28, H 4.61, N 6.63.

[Cp*Rh(μ-bphib)RhCp*](BF_4)$_2$ ([5]): [4] (49.6 mg, 0.1 mmol, 1 eq.) is dissolved in dichloromethane (7 ml) and cooled to 0 °C. [Me_3O]BF_4 (14.7 mg, 0.1 mmol, 1 eq) is added as a solid. The purple reaction mixture is stirred at 0 °C for 30 min, after which the cooling bath is removed and the reaction is stirred at room temperature for 72 h, giving a dark red solution. The crude product is precipitated by the addition of hexane (20 ml). After removal of the supernatant, the residue is washed with hexane (10 ml) and dried under vacuum. The pure product is obtained as red crystals by layering a concentrated dichloromethane solution with pentane (21 mg, 0.023 mmol, 46 %). Crystals suitable for X-ray diffraction are obtained by vapor diffusion of pentane into a dichloroethane solution.
^{1}H NMR (700 MHz, CD_2Cl_2): δ = 7.75 (td, *J* = 7.6, 1.6 Hz, 2 H), 7.69 – 7.58 (m, 2 H), 7.55 – 7.42 (m, 2 H), 7.28 (d, *J* = 7.9 Hz, 2 H), 7.18 (d, *J* = 7.9 Hz, 2 H), 6.12 (dt, *J* = 5.5, 2.8 Hz, 2 H), 5.91 (dt, *J* = 5.0, 2.8 Hz, 2 H), 1.84 (s, 15 H), 1.37 (s, 15 H) ppm.
^{13}C{^{1}H}-NMR (176 MHz, CD_2Cl_2, 25 °C): δ = 148.4, 139.4, 131.3, 131.2, 128.6, 126.4, 125.5, 105.1 (d, $^1J_{Rh,H}$ = 7.7 Hz), 98.7 (d, $^1J_{Rh,H}$ = 7.7 Hz), 91.1 (d, $^1J_{Rh,H}$ = 6.7 Hz), 83.9 (d, $^1J_{Rh,H}$ = 5.0 Hz), 10.2, 9.4 ppm.
HR-MS (ESI): *m/z* = 367.0790 [M^{2+}], calcd. for [M^{2+}] $C_{38}H_{44}N_2Rh_2^{2+}$: *m/z* = 367.0779.

NMR Spectra

[Cp*Rh(bmsab)] ([1])

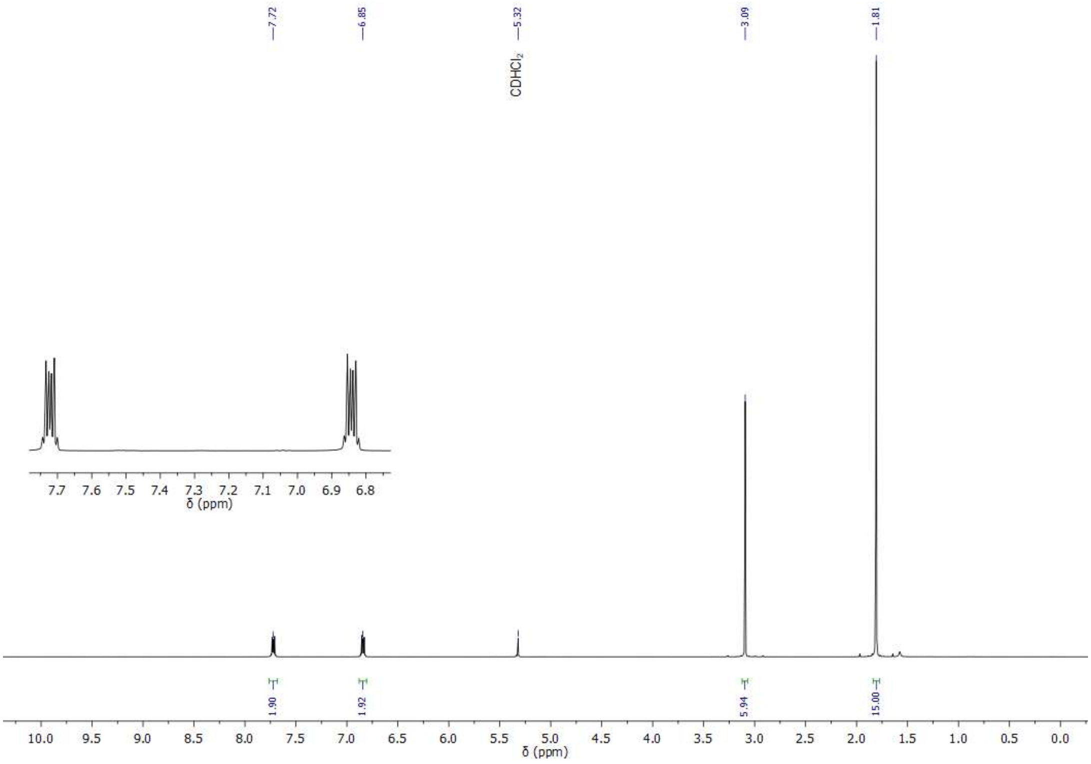

Figure S1: ^{1}H-NMR-Spectrum (400 MHz, CD_2Cl_2, 25 °C) of compound **[1]**.

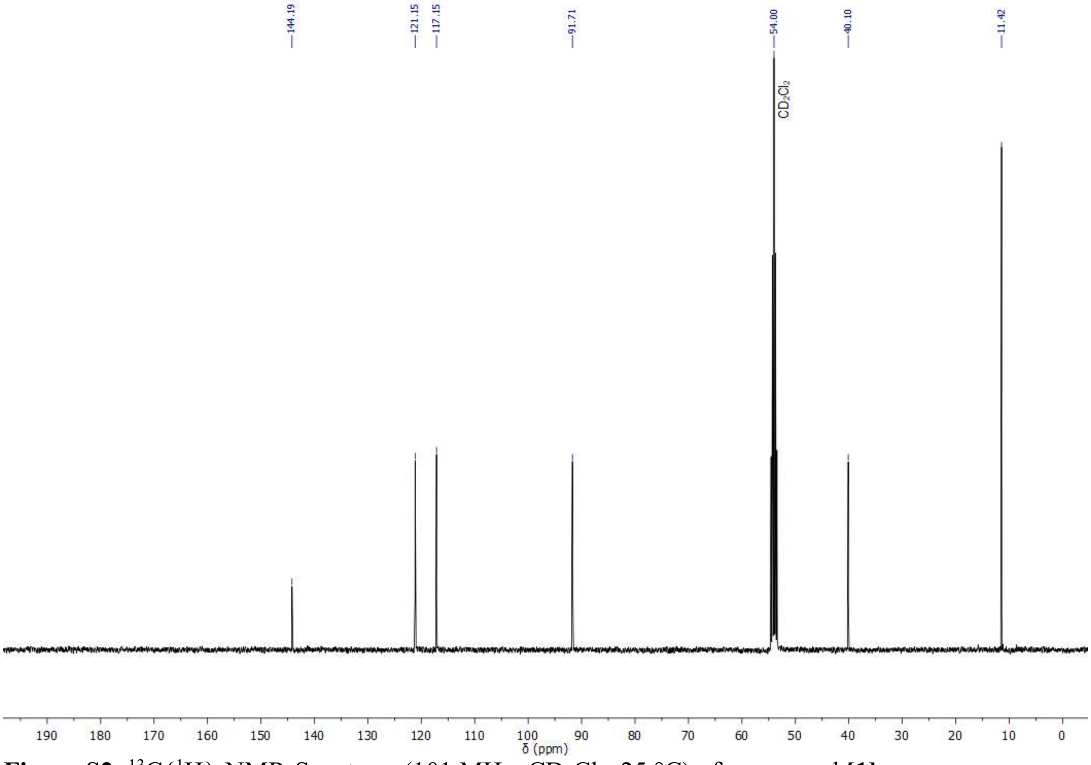

Figure S2: $^{13}C\{^1H\}$-NMR-Spectrum (101 MHz, CD_2Cl_2, 25 °C) of compound **[1]**.

[Cp*Rh(amsab)] ([2])

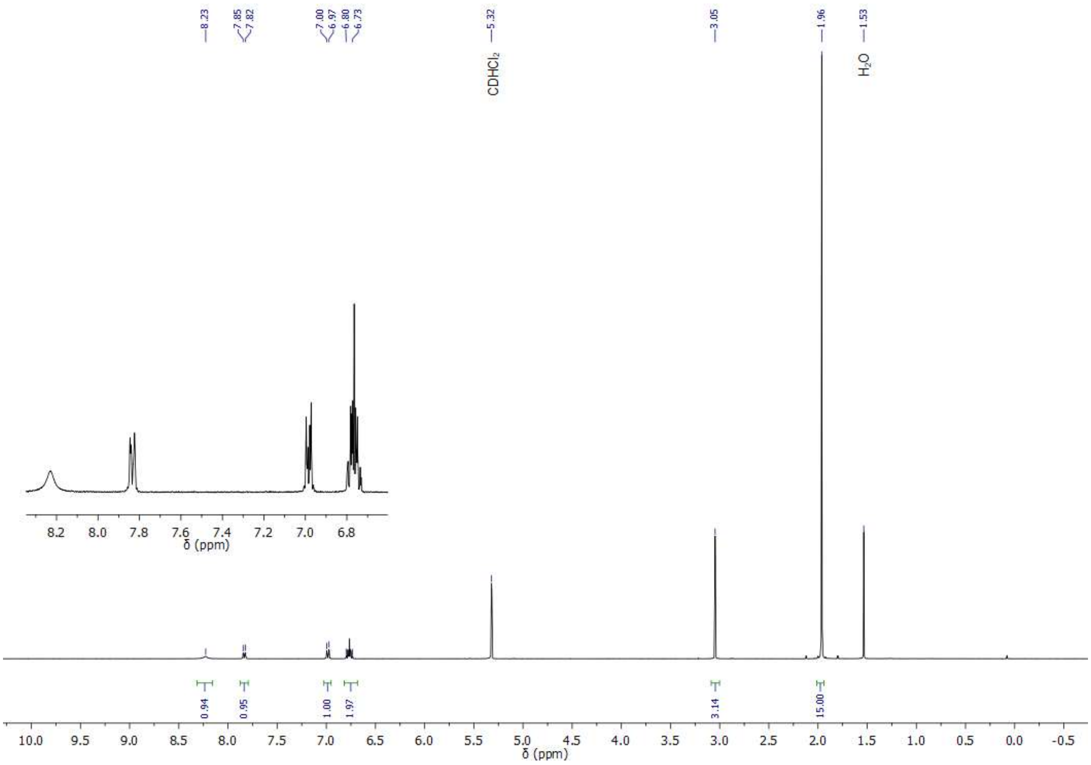

Figure S3: ^{1}H-NMR-Spectrum (400 MHz, CD_2Cl_2, 25 °C) of compound **[2]**.

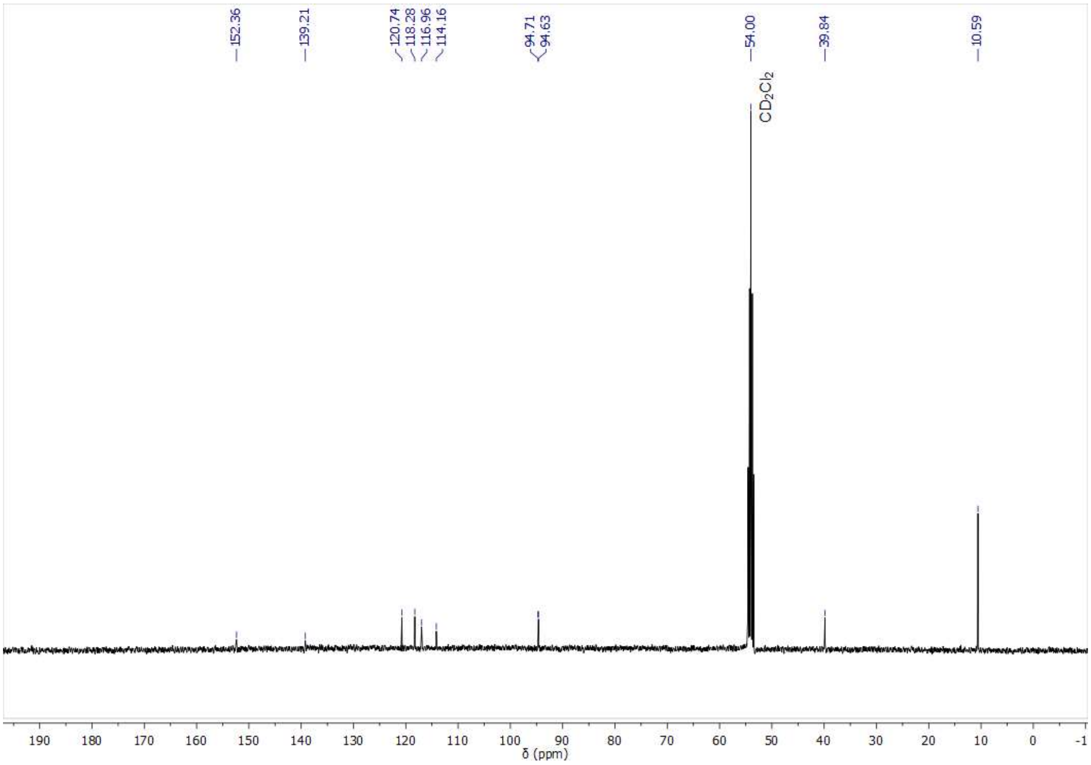

Figure S4: $^{13}C\{^{1}H\}$-NMR-Spectrum (101 MHz, CD_2Cl_2, 25 °C) of compound **[2]**.

[Cp*Rh(bphab)] ([3])

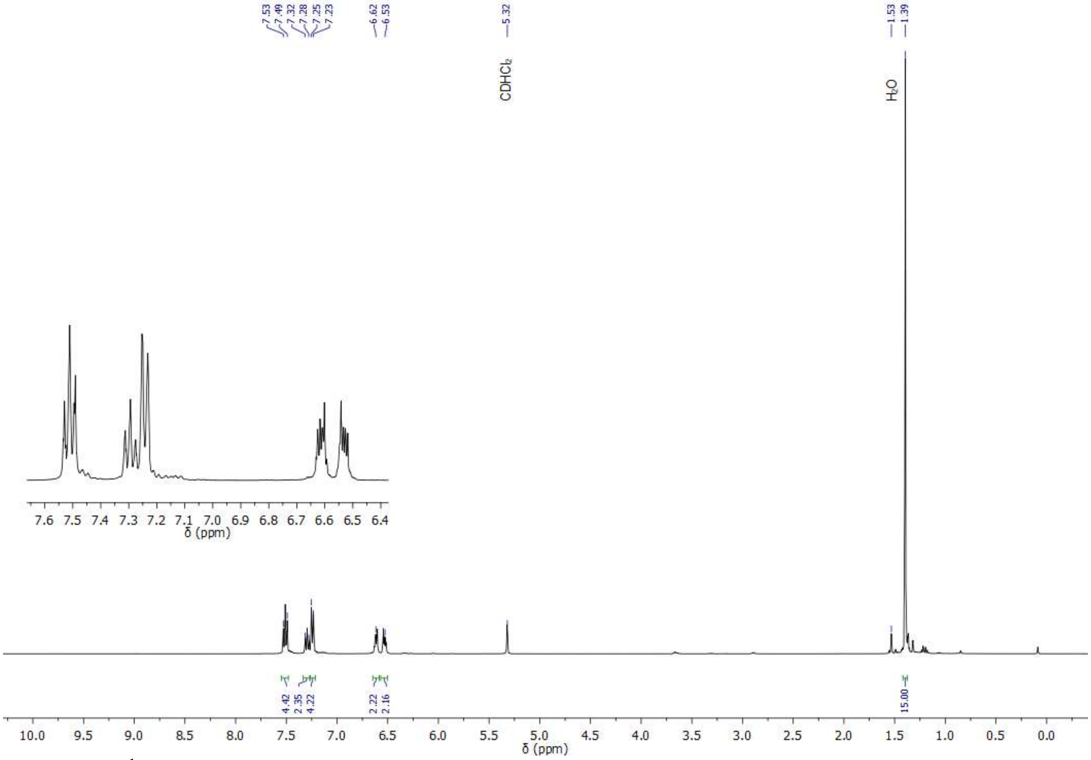

Figure S5: ^{1}H-NMR-Spectrum (400 MHz, CD_2Cl_2, 25 °C) of compound **[3]**.

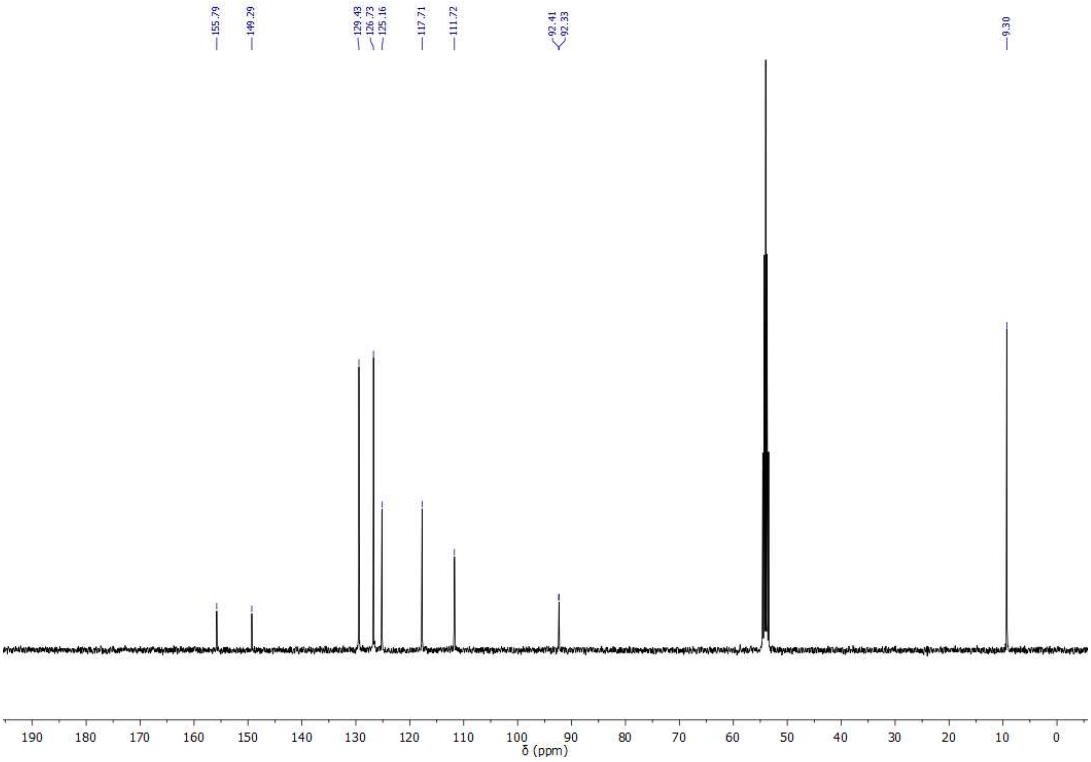

Figure S6: $^{13}C\{^{1}H\}$-NMR-Spectrum (101 MHz, CD_2Cl_2, 25 °C) of compound **[3]**.

[Cp*Rh(aphab)] ([4])

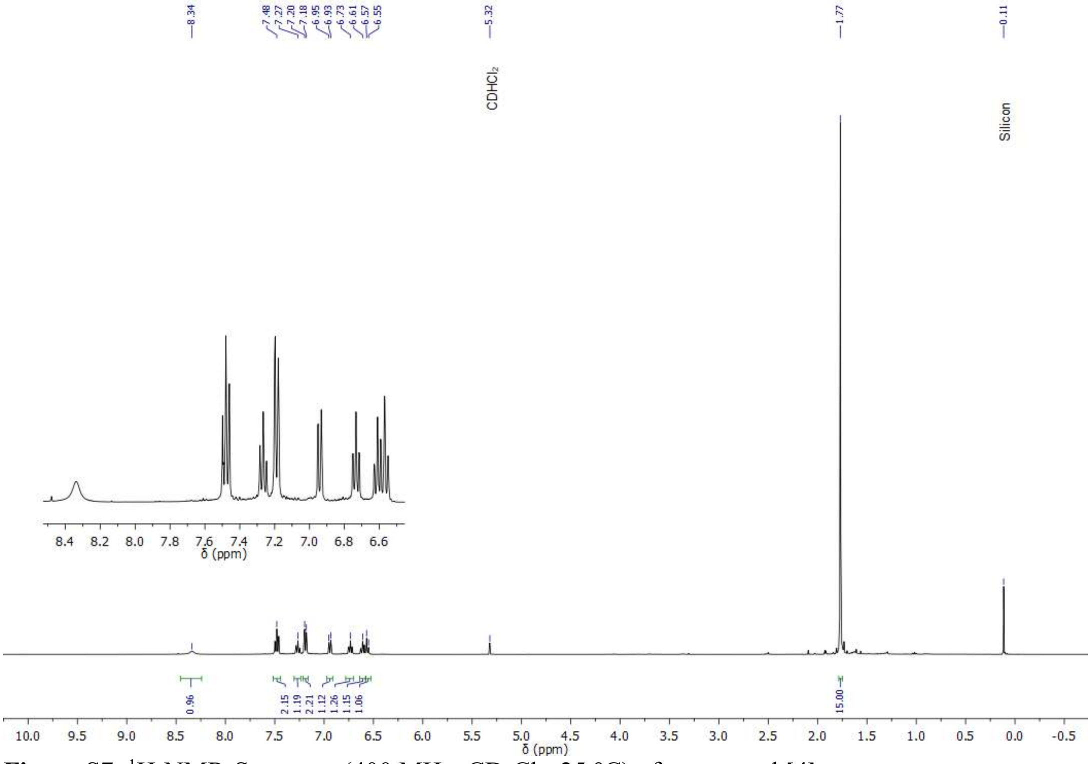

Figure S7: ^{1}H-NMR-Spectrum (400 MHz, CD_2Cl_2, 25 °C) of compound **[4]**.

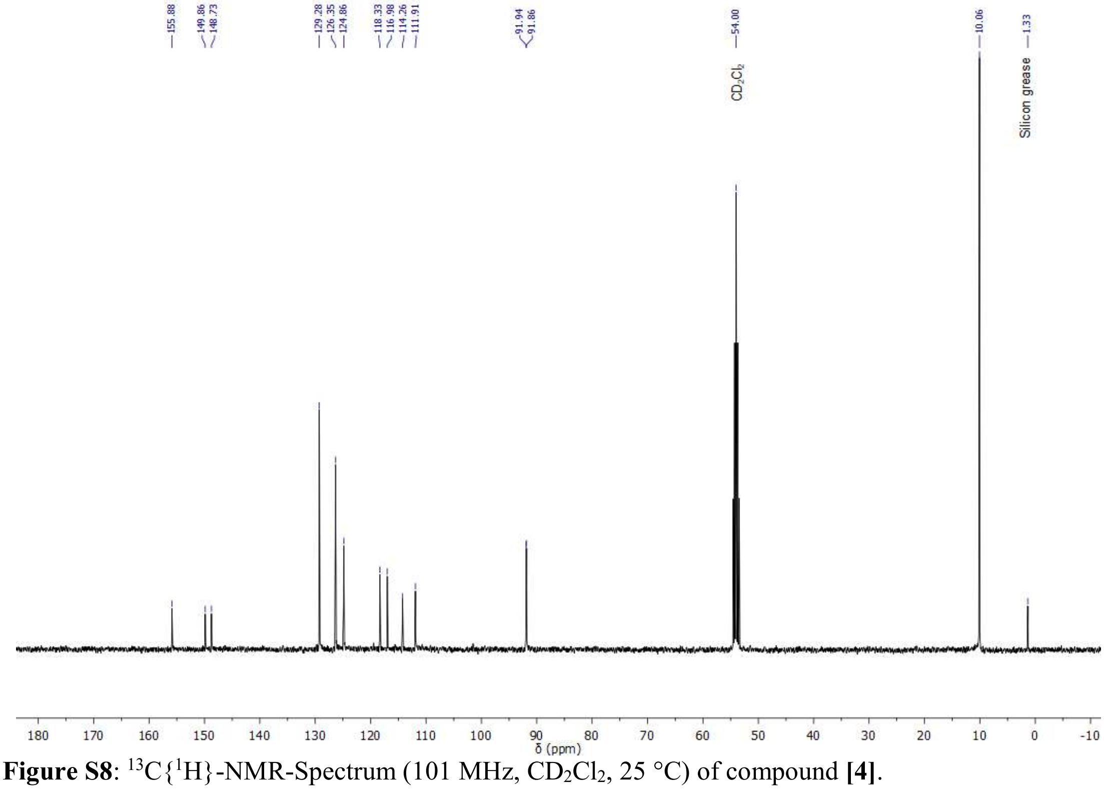

Figure S8: ^{13}C{^{1}H}-NMR-Spectrum (101 MHz, CD_2Cl_2, 25 °C) of compound **[4]**.

[Cp*Rh(bphiq)MeCN] ([5])

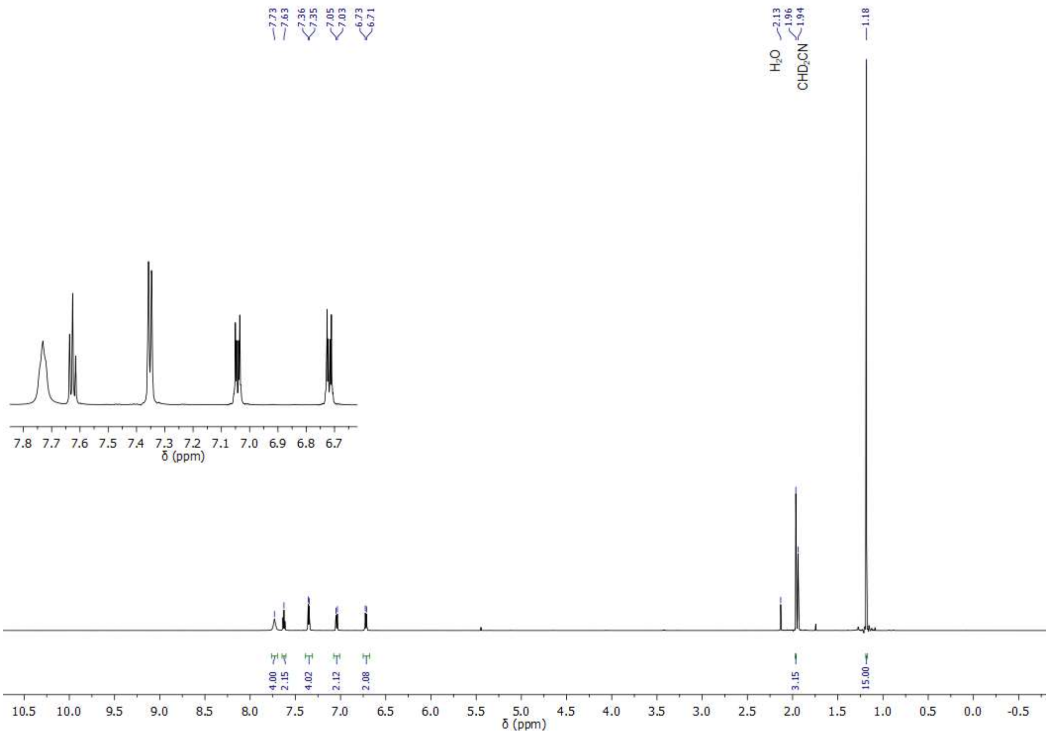

Figure S9: ^{1}H-NMR-Spectrum (700 MHz, CD_2Cl_2, 25 °C) of compound **[5]**.

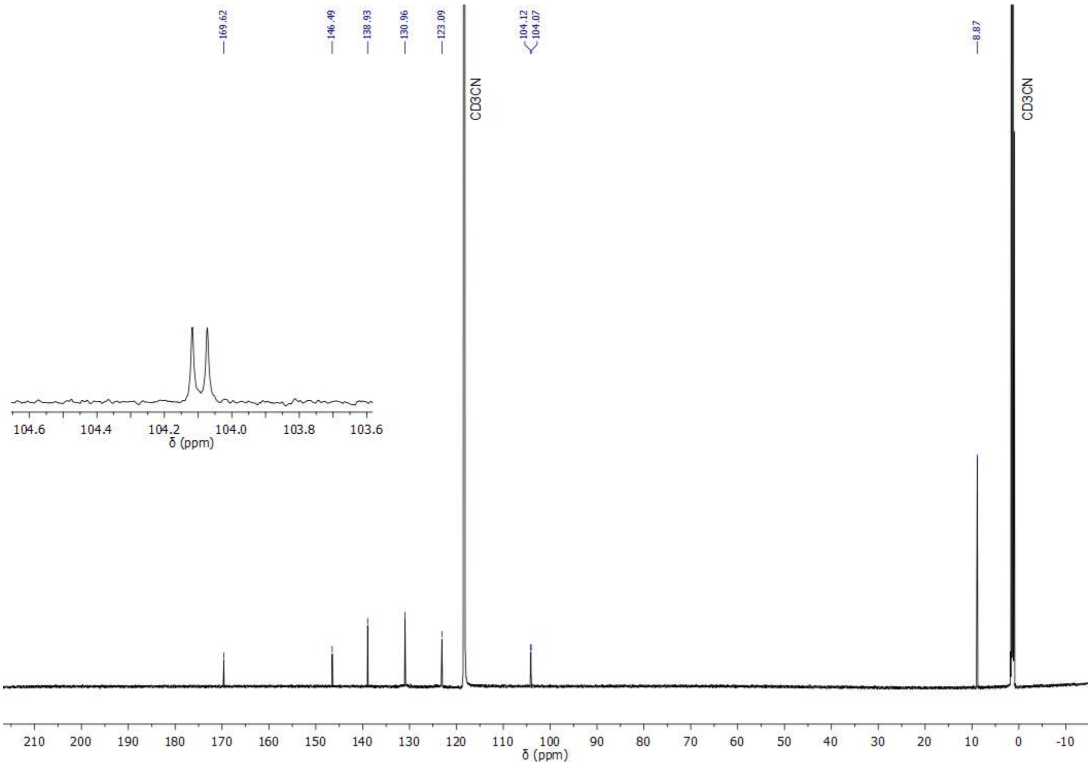

Figure S10: ^{13}C-NMR-Spectrum (176 MHz, CD_2Cl_2, 25 °C) of compound **[5]**.

[Cp*Rh(μ-bphiq)RhCp*] ([6])

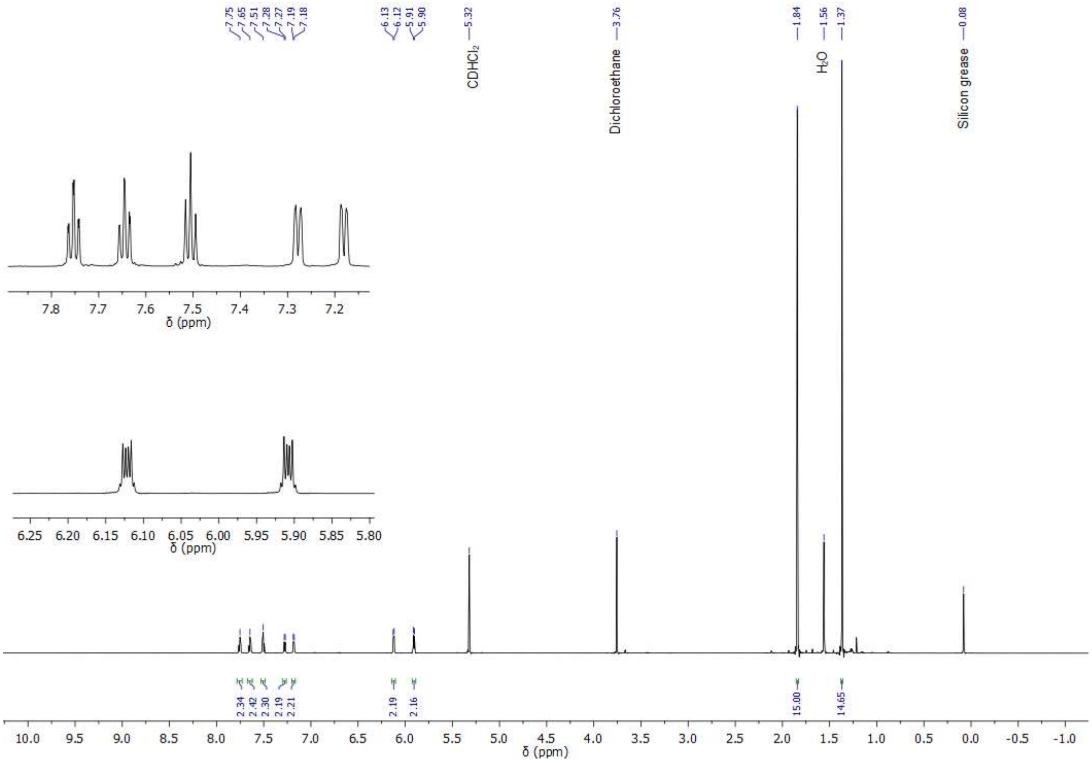

Figure S11: ^{1}H-NMR-Spectrum (700 MHz, CD_2Cl_2, 25 °C) of compound **[6]**.

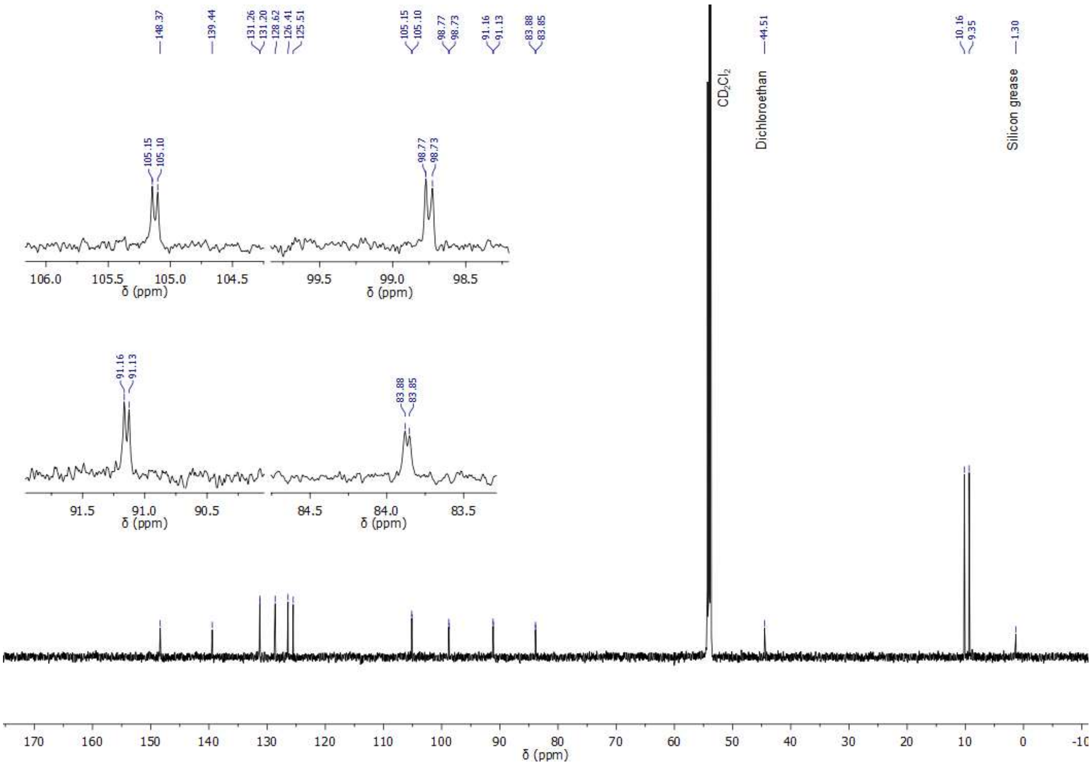

Figure S12: ^{13}C-NMR-Spectrum (176 MHz, CD_2Cl_2, 25 °C) of compound **[6]**.

Electrochemistry

Cyclic voltammetry of [Cp*Rh(bmsab)·MeCN] ([1·MeCN])

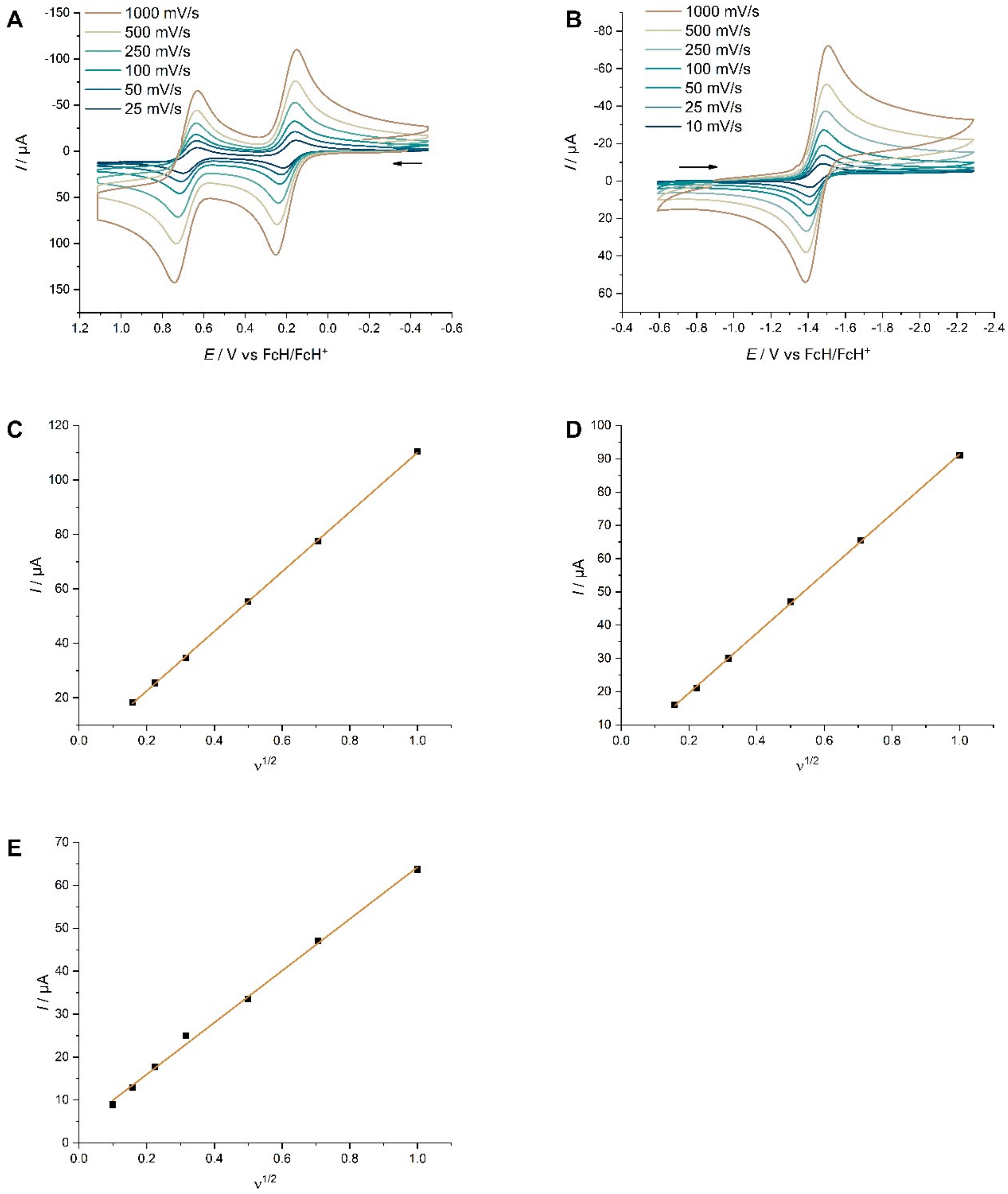

Figure S13: Cyclic voltammetry of complex **[3]**. A: Anodic process at variable scan rates. B: Cathodic process at variable scan rates. C: Randles-Sevcik plot of oxidation. D: Randles-Sevcik plot of reduction.

Table S1: electrochemical data of **[1·MeCN]** in acetonitrile/NBu_4PF_6 0.1 M. Potentials given against FcH/FcH^+.

	$E_{1/2}$ / V	□E / V(100 mV/s)	I_a / µA (100 mV/s)	I_a/I_c
**1st Oxidation**	0.20	0.072	28	1.13
**2nd Oxidation**	0.67	0.072	19	1.12
**Reduction**	−1.44	0.074	23	0.9

Cyclic voltammetry of [Cp*Rh(amsab)] ([2])

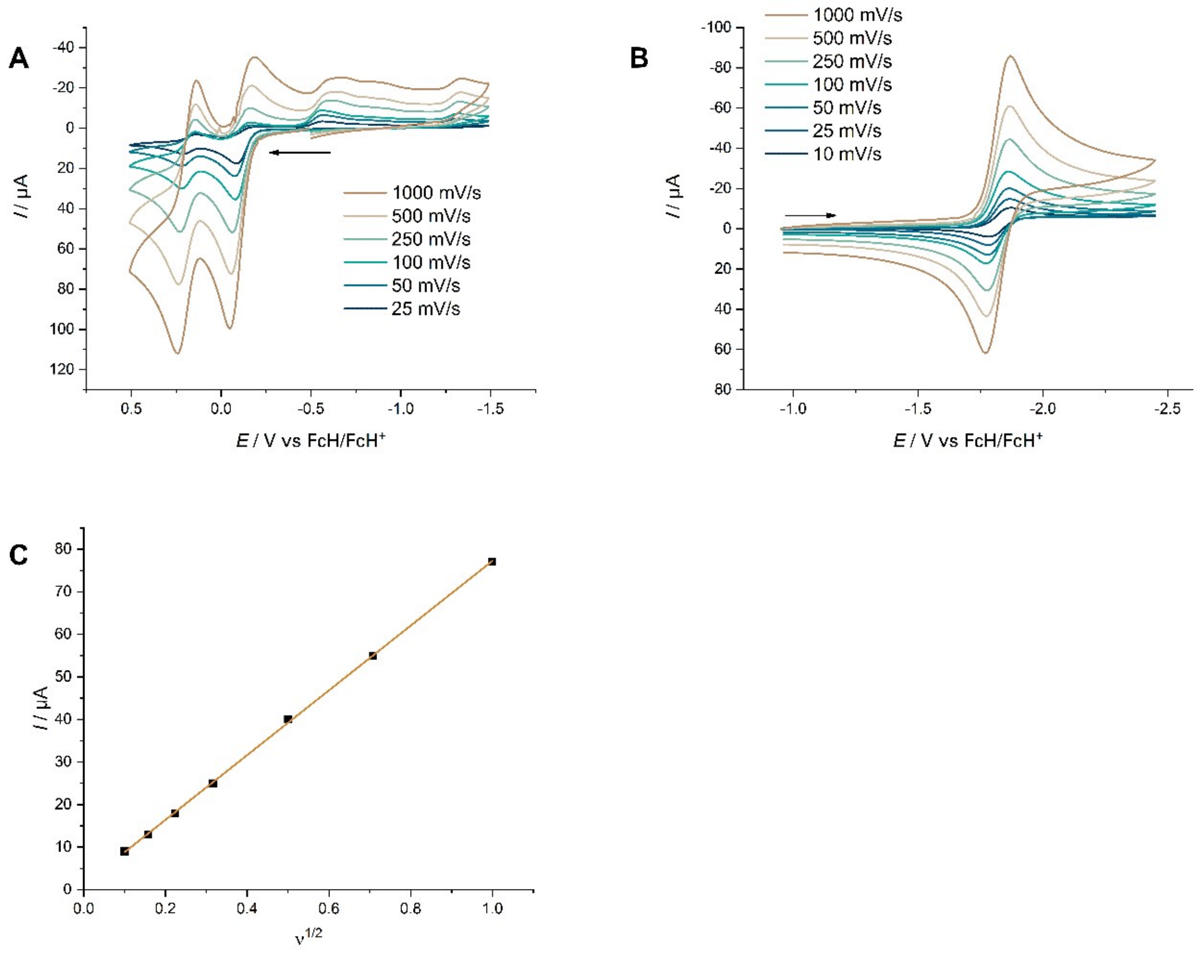

Figure S14: Cyclic voltammetry of complex **[2]**. A: Anodic processes at variable scan rates. B: Cathodic process at variable scan rates. C: Randles-Sevcik plot of reduction.

Table S2: electrochemical data of **[2]** in acetonitrile/NBu_4PF_6 0.1 M. Potentials given against FcH/FcH^+.

	$E_{1/2}$ / V	□E / V(100 mV/s)	I_a / µA (100 mV/s)	I_a/I_c (100 mV/s)
**1st Oxidation**	−0.11	0.080	30	3.15
**2nd Oxidation**	0.18	0.070	9.5	1.11
**Reduction**	−1.86	0.090	25	0.96

Cyclic voltammetry of [Cp*Rh(bphab)] ([3])

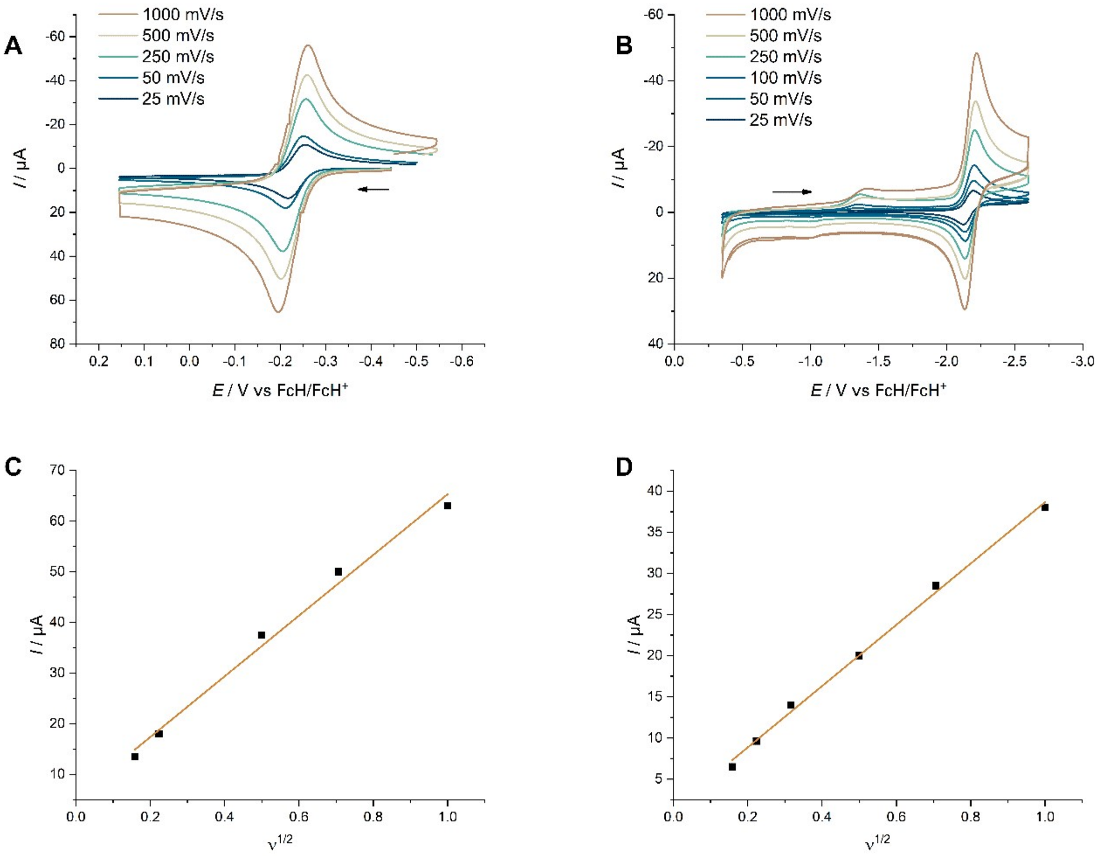

Figure S15: Cyclic voltammetry of complex **[3]**. A: Anodic process at variable scan rates. B: Cathodic process at variable scan rates. C: Randles-Sevcik plot of oxidation. D: Randles-Sevcik plot of reduction.

Table S3: Electrochemical data of **[3]** in acetonitrile/$NBu_4PF_6$0.1 M. Potentials given against FcH/FcH^+.

	**$E_{1/2}$ / V**	**□E / V(50 mV/s)**	**I_a / µA (50 mV/s)**	**I_a/I_c (50 mV/s)**
**Oxidation**	−0.23	0.039	18	1.05
**Reduction**	−2.18	0.065	7.9	0.92

Cyclic voltammetry of [Cp*Rh(aphab)] ([4])

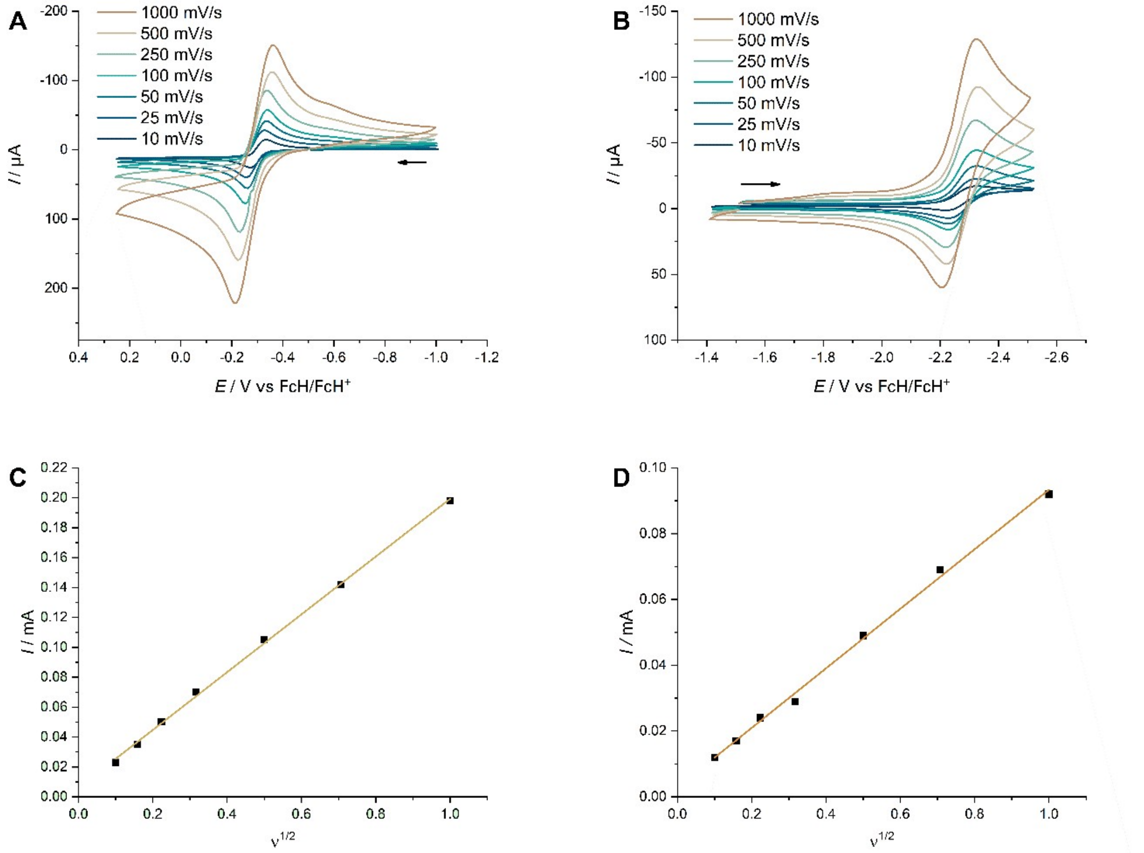

Figure S16: Cyclic voltammetry of complex **[4]**. A: Anodic process at variable scan rates. B: Cathodic process at variable scan rates. C: Randles-Sevcik plot of oxidation. D: Randles-Sevcik plot of reduction.

Table S4: Electrochemical data of **[4]** in acetonitrile/NBu4PF_6 0.1 M. Potentials given against FcH/FcH^+.

	**$E_{1/2}$ / V**	**□E / V(100 mV/s)**	**I_a / µA (100 mV/s)**	**I_a/I_c (100 mV/s)**
**Oxidation**	−0.31	0.10	181	1.1
**Reduction**	−2.27	0.11	82	0.8

EPR Spectroscopy/Spectroelectrochemistry

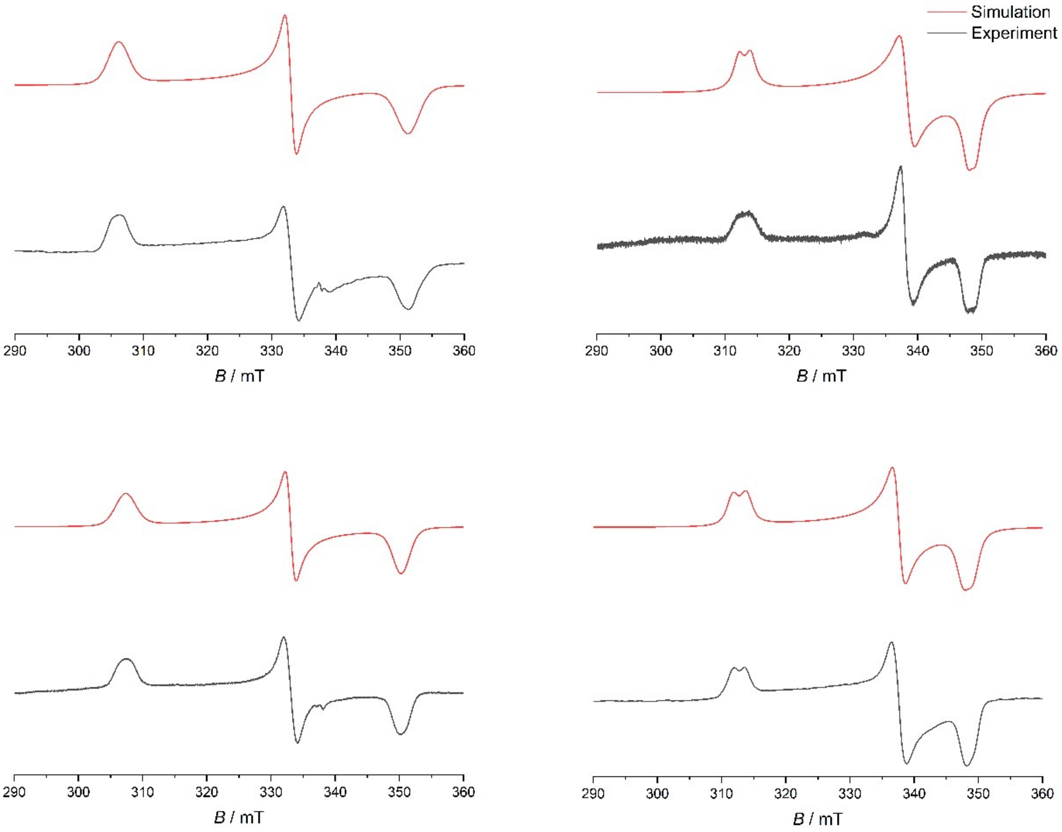

Figure S17: Experimental and simulated spectra of reduced species **[1]** – **[4]**. **[1]**, **[2]**, **[4]** were generated by electrolysis in MeCN/TBAPF$_6$; **[3]** by chemical reduction with KC$_8$ in THF. Experimental spectra recorded at 103 K.

Table S5: Simulated and calculated EPR parameters of **[1]** – **[4]**. As ^{103}Rh hyperfine coupling was not resolved in **[1]** and **[2]**, spectra were simulated by anisotropic line broadening via H-Strain.

	**[1]**	**[2]**	**[3]**	**[4]**
**g-values**	1.92 (1.94)	1.93 (1.96)	1.94 (1.96)	1.94 (1.96)
	2.03 (2.03)	2.03 (2.02)	2.00 (2.00)	2.00 (2.00)
**(calc.)**	2.21 (2.16)	2.20 (2.13)	2.16 (2.12)	2.16 (2.12)
**A / MHz**	---	---	35	43
			0.1	0.1
			55	64
**H-Strain**	105	69	---	---
	30	27		
	110	99		
**Line-width**	0.6 mT	0.6 mT	1.2 mT	0.9 mT

UV/Vis-NIR-Spectroscopy/Spectroelectrochemistry

Oxidations

[1]

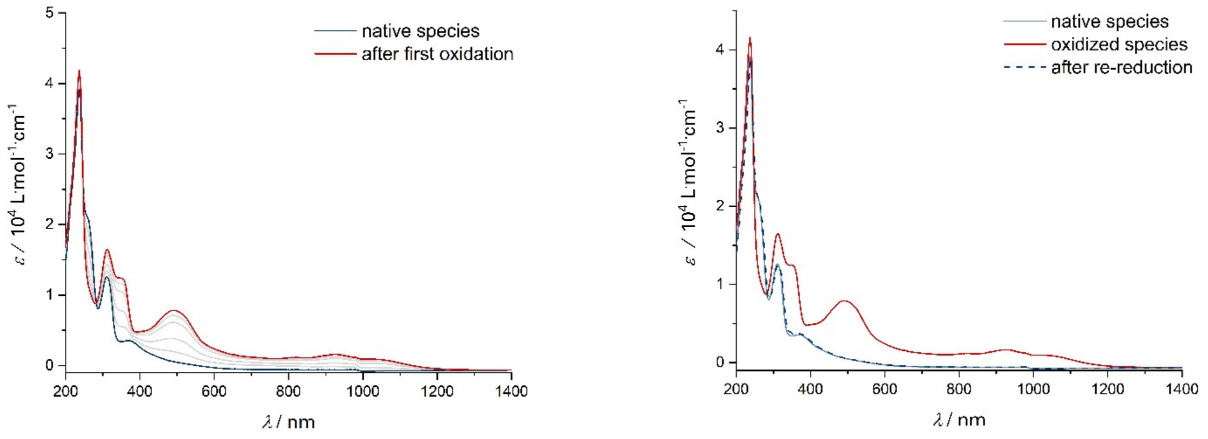

Figure S18: Spectral changes during first oxidation of **[1]** in acetonitrile/NBu_4PF_6, OTTLE cell, Pt WE (left). The overlay of spectra before oxidation, after oxidation and after re-reduction (right) confirms the chemical reversibility of the process.

[3]

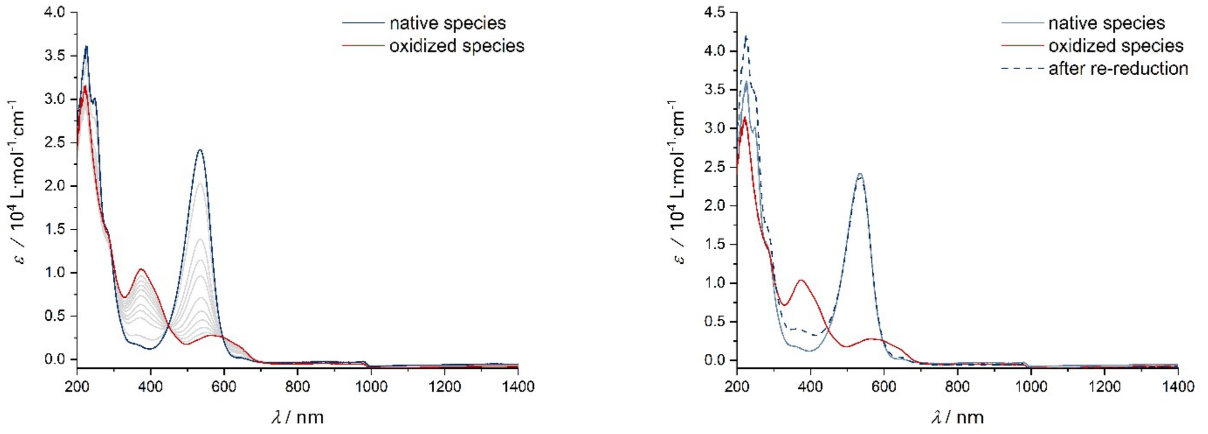

Figure S19: Spectral changes during oxidation of **[3]** in acetonitrile/NBu_4PF_6, OTTLE cell, Pt WE (left). The overlay of spectra before oxidation, after oxidation and after re-reduction (right) confirms the chemical reversibility of the process.

[4]

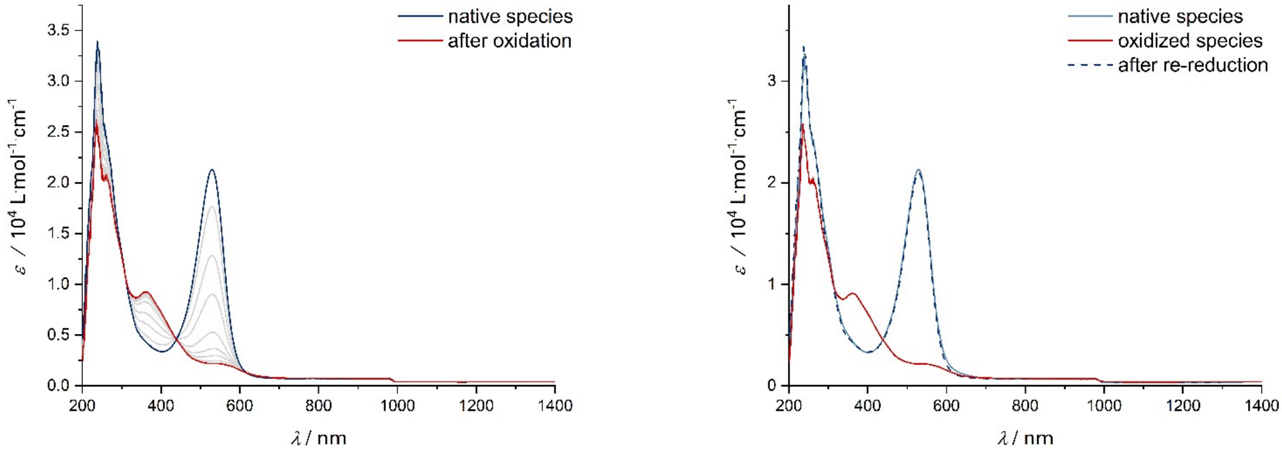

Figure S20: Spectral changes during oxidation of **[4]** in acetonitrile/NBu_4PF_6, OTTLE cell, Pt WE (left). The overlay of spectra before oxidation, after oxidation and after re-reduction (right) confirms the chemical reversibility of the process.

Reductions

[1]

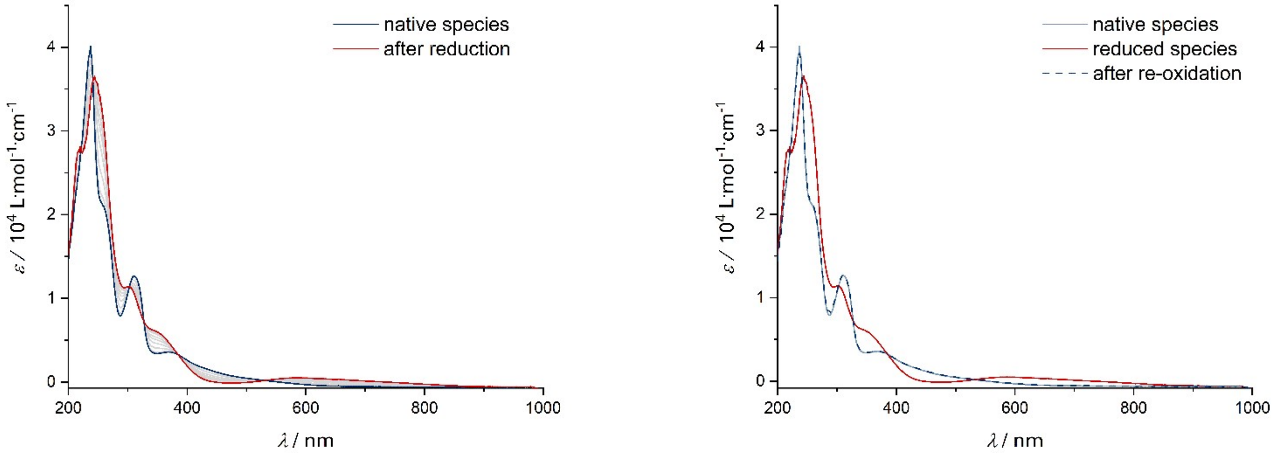

Figure S21: Spectral changes during reduction of **[1]** in acetonitrile/NBu_4PF_6, OTTLE cell, Pt WE (left). The overlay of spectra before reduction, after reduction and after re-oxdiation (right) confirms the chemical reversibility of the process.

[2]

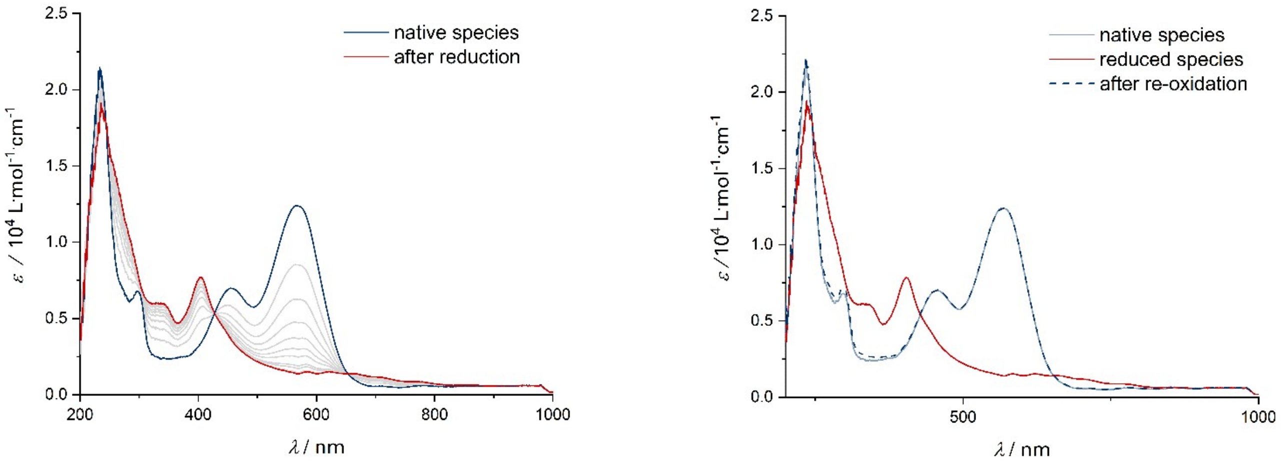

Figure S22: Spectral changes during reduction of **[2]** in acetonitrile/NBu_4PF_6, OTTLE cell, Pt WE (left). The overlay of spectra before reduction, after reduction and after re-oxdiation (right) confirms the chemical reversibility of the process.

[3]

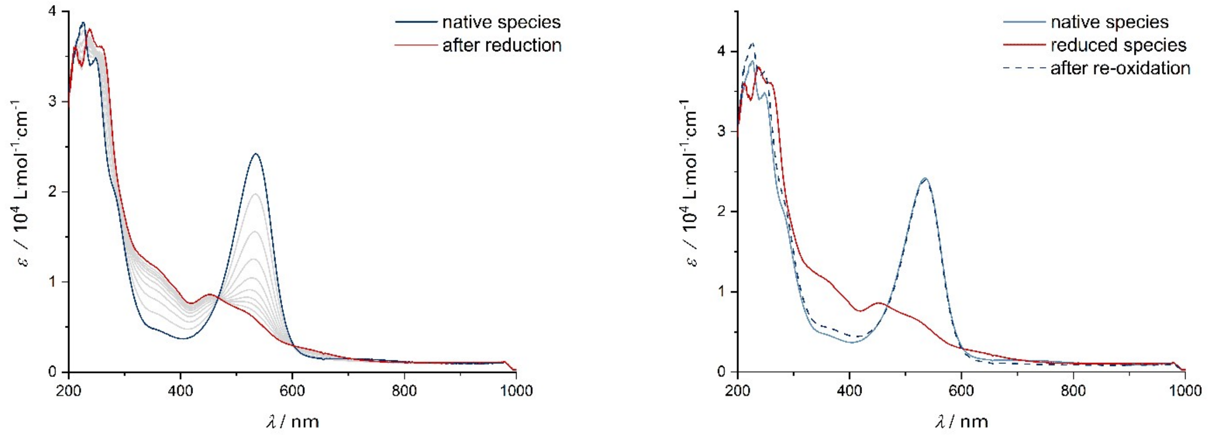

Figure S23: Spectral changes during reduction of **[3]** in acetonitrile/NBu_4PF_6, OTTLE cell, Pt WE (left). The overlay of spectra before reduction, after reduction and after re-oxdiation (right) confirms the chemical reversibility of the process.

[4]

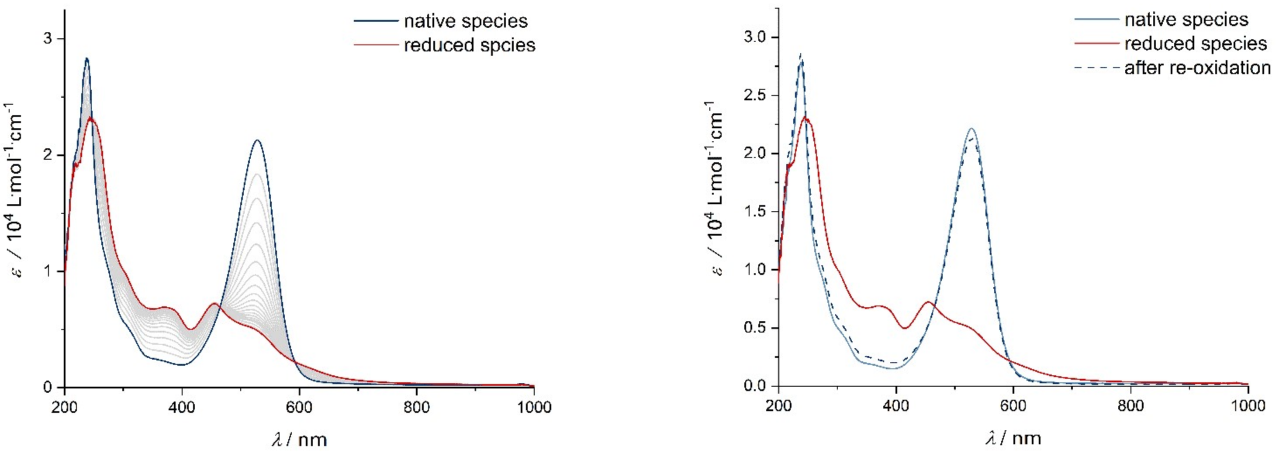

Figure S24: Spectral changes during reduction of **[4]** in acetonitrile/NBu_4PF_6, OTTLE cell, Au WE (left). The overlay of spectra before reduction, after reduction and after re-oxdiation (right) confirms the chemical reversibility of the process.

Spectra of native species in DCM and MeCN

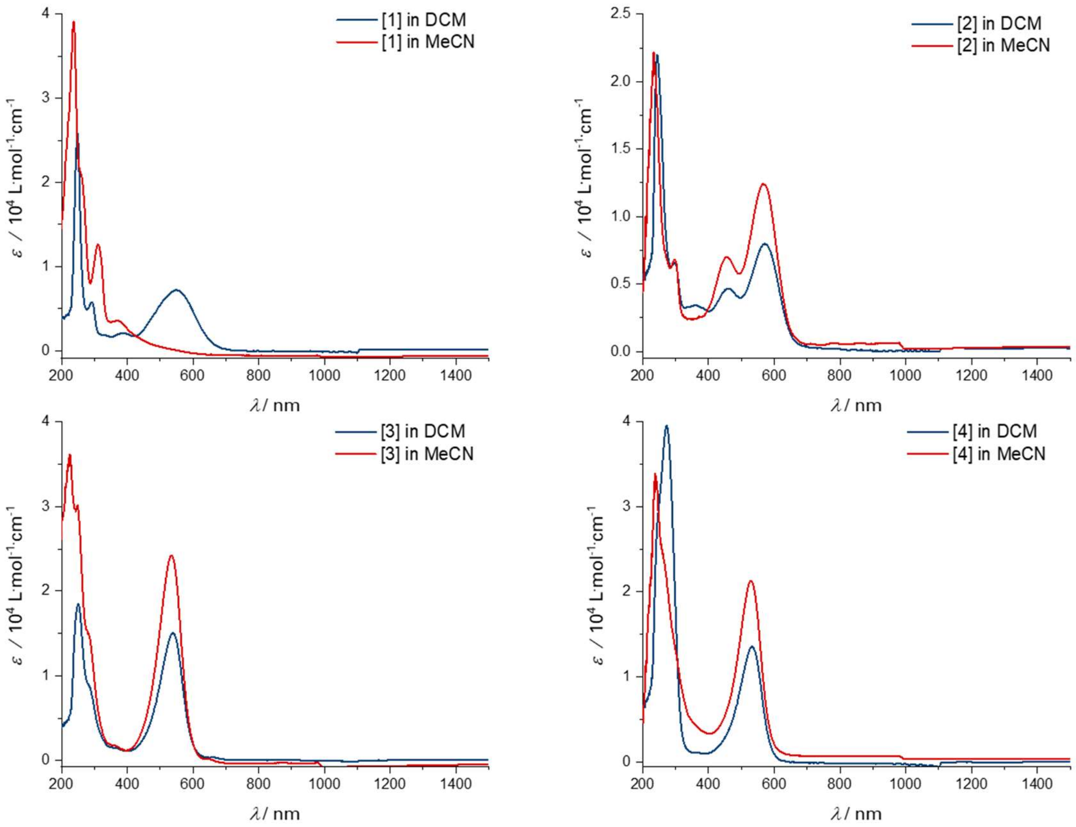

Figure S25: UV/Vis/NIR spectra of native complexes in DCM (blue traces) and acetonitrile (red traces).

Reactivity studies

a) Reaction of [CoCp*$_2$][1] with O$_2$

An EPR tube was charged with solution of **[CoCp*$_2$][1]** in dry and degassed acetonitrile, placed in the EPR spectrometer and treated with O_2 gas at 0 °C. The reaction was monitored by EPR spectroscopy to detect spectral signatures of an O_2 adduct. The shift to a g_{iso} value closer to g_{el} indicates a shift of electron density away from the metal center, which indicates formation of a superoxido complex. The anisotropic g values are in accordance with previously published superoxido complexes of Rhodium as shown in table S6.

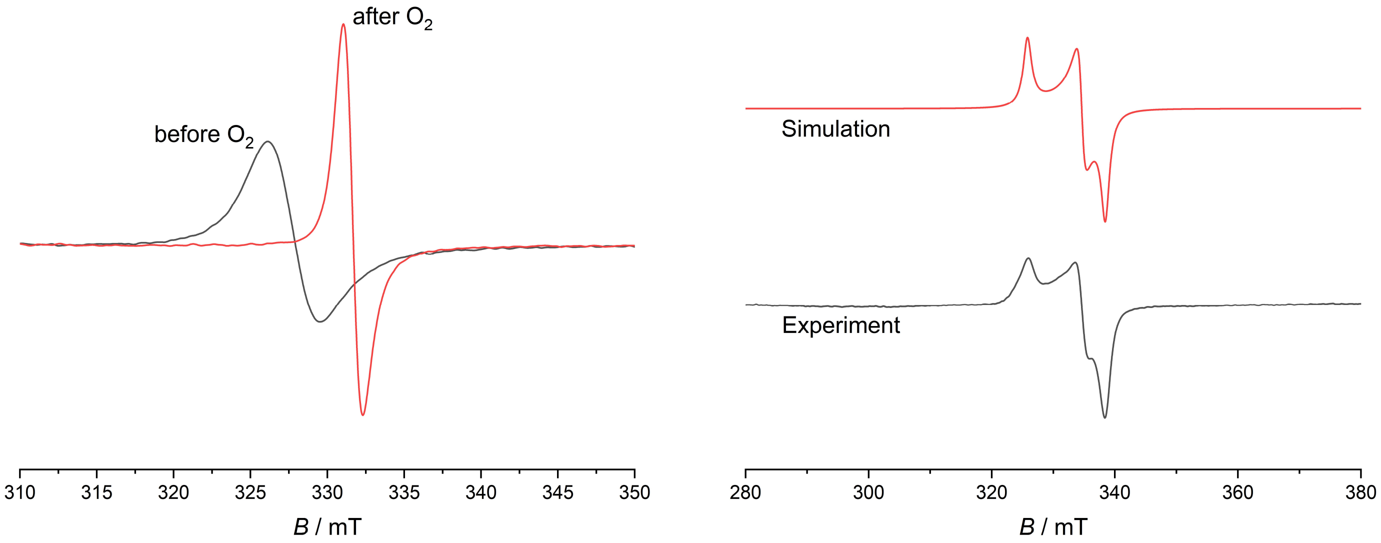

Figure S26: EPR monitoring of the reaction of reduced **[1]**$^-$ with O_2. Left: Shift in g_{iso} upon reaction with O_2. Right: Anisotropic spectrum in frozen acetontrile.

Table S6: Simulated g-values of the O_2 adduct of **[1]**$^-$ in comparison with reported Rh superoxido complexes.

	g_1	g_2	g_3
**[1-O$_2$]$^-$**	**2.085**	**2.019**	**1.995**
**[(TPP)RhO$_2$]**	2.084	2.025	1.993
**[(TPP)RhO$_2$(POEt$_3$)]**[2]	2.086	2.009	2.004
***cis*-[Rh(en)$_2$Cl(O$_2$)]$^+$**[3]	2.0861	2.0229	1.9919

b) Reactivity towards DCM

In order to aid with signal assignment, a separate sample of the Rh^{III} chloride complex **[1-Cl]⁻** was prepared by stirring equimolar amounts of **[1]** and NEt_4Cl in DCM for 20 min and removing the solvent. The ^{1}H-NMR spectrum of **[1-Cl]⁻** shows quantitative conversion and four characteristic resonances at δ = 7.63 (m, 2 H), 6.59 (m, 2 H), 2.58 (s, 6 H) and 1.54 (s, 15 H) ppm.

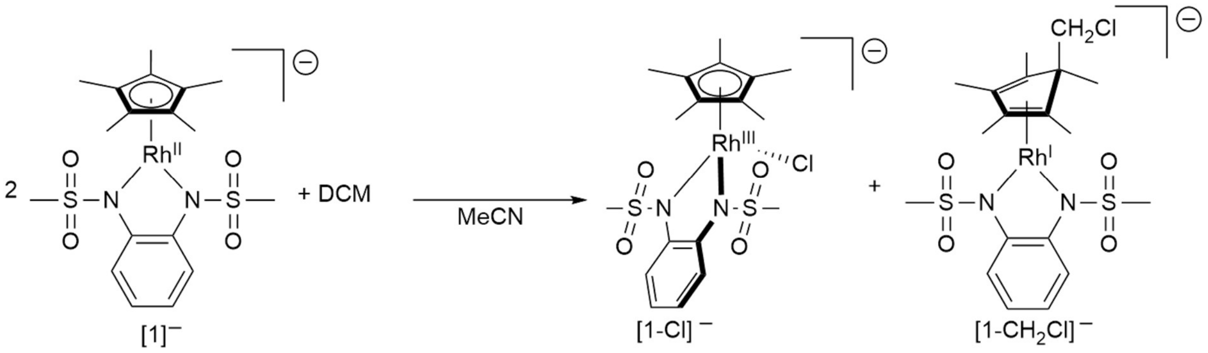

Scheme S2: The reaction of **[1]⁻** with DCM.

[CoCp*₂][1] (20 mg, 0.024 mmol, 1 eq) was dissolved in MeCN and an excess of dichloromethane was added. After 4 h, the green solution had turned yellow. The solvent was removed and the residue washed with diethyl ether to give a sticky red solid. All solids were dissolved in MeCN-*d₃* and investigated by ^{1}H- and ^{13}C-NMR. The $[CoCp*_2]^+$ resonance at δ = 1.70 ppm was used as an internal reference to determine the spectroscopic yield (100 % yield would correspond to a ratio of 1:15 of one aromatic phenylene multiplet to the $[CoCp*_2]^+$ resonance). In the reaction mixture, two separate sets of signals can be discerned. One set of signals can be assigned to **[1-Cl]⁻** by comparison with the authentic sample described above. By comparing integrals with the $[CoCp*_2]^+$ resonance, a yield of approximately 50 % for **[1-Cl]⁻** is determined. The second set of signals comprises three resonances that can be attributed to the bmsab ligand, but shows no Cp* signal. Instead, four singlet signals with an intensity ration of 2:6:3:6 are observed. The chemical shifts and intensities of these (δ = 2.95, 2.01, 1.75 and 1.02 ppm) are comparable to the signals described by Kölle for an exo-functionalized cyclopentadiene bound to Co^I: δ = 2.58 (2 H), 1.75 (6 H), 1.50 (3 H) and 0.75 (6 H) ppm.[23] Additionally, the ^{13}C spectrum shows three doublet signals (δ = 90.4, 67.0, 60.05 ppm) with coupling constants of 11.2 Hz, 11.4 Hz and 4.4 Hz. This indicates an η4-bound cyclopentadiene. The HMBC spectrum shows coupling between these ring carbons and the singlets described above, confirming the presence of a functionalized cyclopentadiene ligand bound to Rh. The constitution is confirmed by the expected signals for **[1-CH₂Cl]⁻** in the mass spectrum. By comparing intensity ratios, a yield of approximately 20 % is determined. Based on the stoichiometry given in scheme S2, one would expect a 1:1 ratio of **[1-Cl]⁻** and **[1-CH₂Cl]⁻**. We tentatively attribute the deviation from the ideal 1:1 ratio to the previously described instability of exo-functionalized cyclopentadienes towards further reactivity.[23] All spectra are given below.

NEt$_4$[1-Cl]:

^{1}H NMR (250 MHz, CD_3CN, 25 °C): δ = 7.63 (m, 2 H), 6.59 (m, 2 H), 3.16 (q, Hz, 8 H, NEt$_4$), 2.58 (s, 6 H), 1.54 (s, 15 H), 1.20 (m, 12 H, NEt$_4$) ppm.

^{13}C{^{1}H}-NMR (176 MHz, CD_3CN, 25 °C): δ = 145.9, 120.9, 120.3, 95.5, 53.1 (NEt$_4^+$), 39.9, 9.4, 7.7 (NEt$_4^+$) ppm.

HR-MS (ESI): *m/z* = 534.9996 [M$^-$], calcd. for [M$^-$] $C_{18}H_{25}N_2O_4S_2RhCl^-$: *m/z* = 534.9994.

[CoCp*$_2$][1-Cl]:

^{1}H NMR (700 MHz, CD_3CN, 25 °C): δ = 7.66 (m, 2 H), 6.57 (m, 2 H), 2.60 (s, 6 H), 1.70 (s, 60 H, [CoCp*$_2$]$^+$), 1.52 (s, 15 H) ppm.

^{13}C{^{1}H}-NMR (176 MHz, CD_3CN, 25 °C): δ = 146.1, 120.7, 120.5, 95.2 ([CoCp*$_2$]$^+$), 94.8, 39.9, 9.4, 8.3 ([CoCp*$_2$]$^+$) ppm.

HR-MS (ESI): *m/z* = 535.0009 [M$^-$], calcd. for [M$^-$] $C_{18}H_{25}N_2O_4S_2RhCl^-$: *m/z* = 534.9994.

[CoCp*$_2$][1-CH$_2$Cl]:

^{1}H NMR (700 MHz, CD_3CN, 25 °C): δ = 7.25 (m, 2 H), 6.52 (m, 2 H), 2.95 (s, 2 H,), 2.65 (s, 6 H), 2.01 (s, 6 H), 1.75 (2, 3 H), 1.70 (s, 155 H, [CoCp*$_2$]$^+$) 1.02 (s, 6 H) ppm.

^{13}C{^{1}H}-NMR (176 MHz, CD_3CN, 25 °C): δ = 145.8, 120.3, 119.6, 95.2 ([CoCp*$_2$]$^+$), 90.4 (d, 11.4 Hz), 67.0 (d, 11.2 Hz), 60.05 (d, 4.4 Hz), 50.6, 40.5, 19.4, 11.7, 11.4, 8.3 ([CoCp*$_2$]$^+$) ppm.

HR-MS (ESI): *m/z* = 549.0163 [M$^-$], calcd. for [M$^-$] $C_{19}H_{27}N_2O_4S_2RhCl^-$: *m/z* = 549.0161.

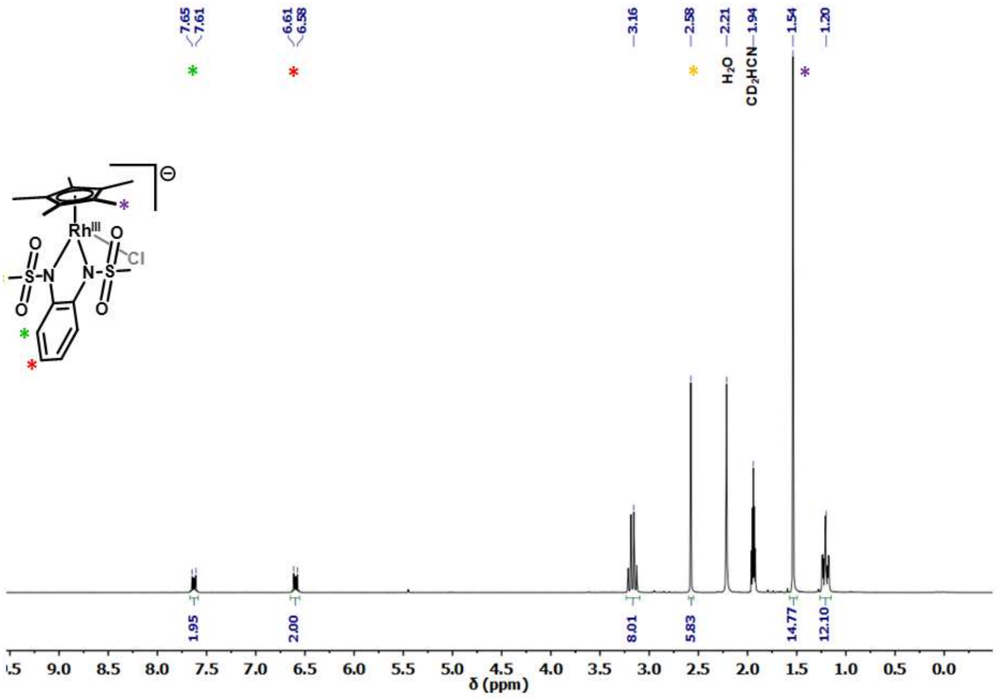

Figure S27: ^{1}H-NMR spectrum of NEt$_4$**[1-Cl]**.

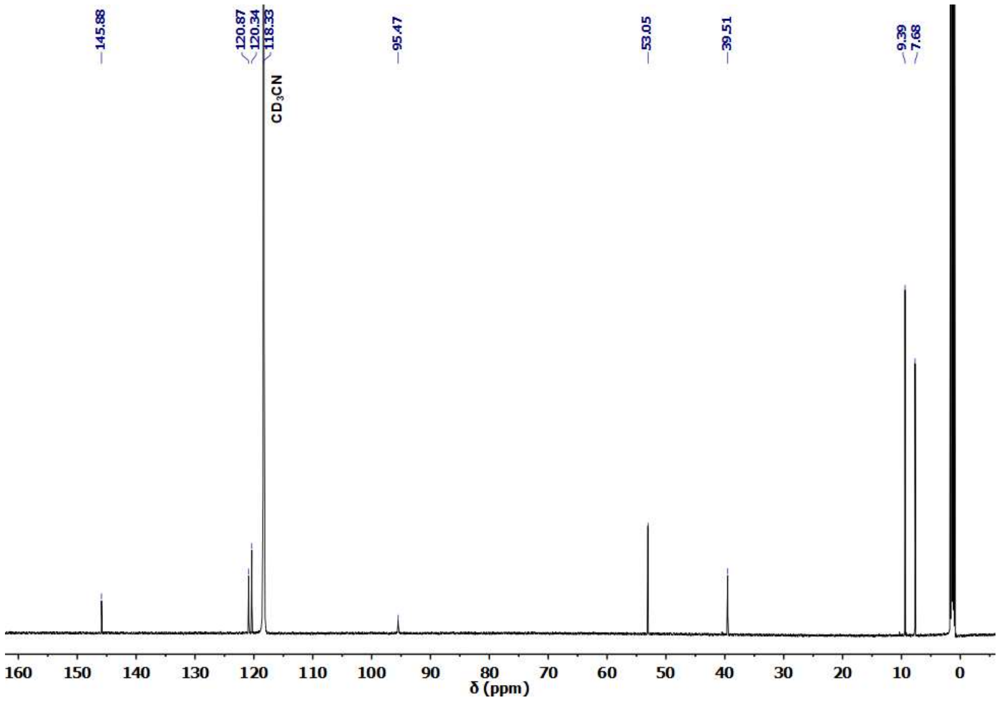

Figure S28: ^{13}C-NMR spectrum of NEt_4**[1-Cl]**.

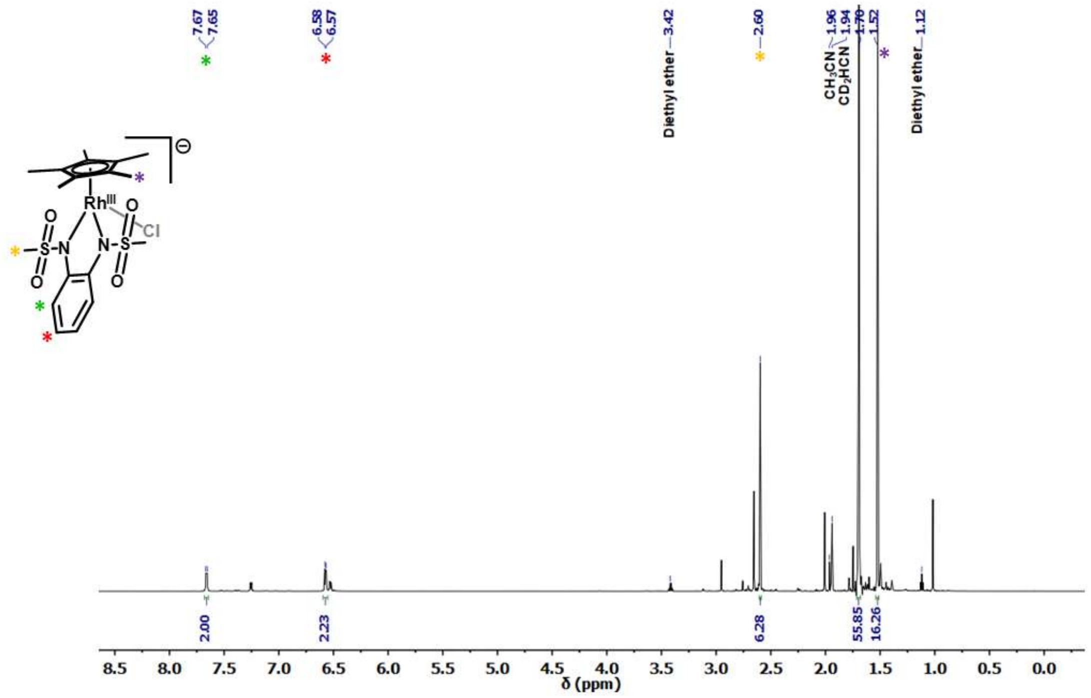

Figure S29: ^{1}H-NMR spectrum of the product mixture after reaction with DCM. Assignments given for **[1-Cl]**$^{-}$.

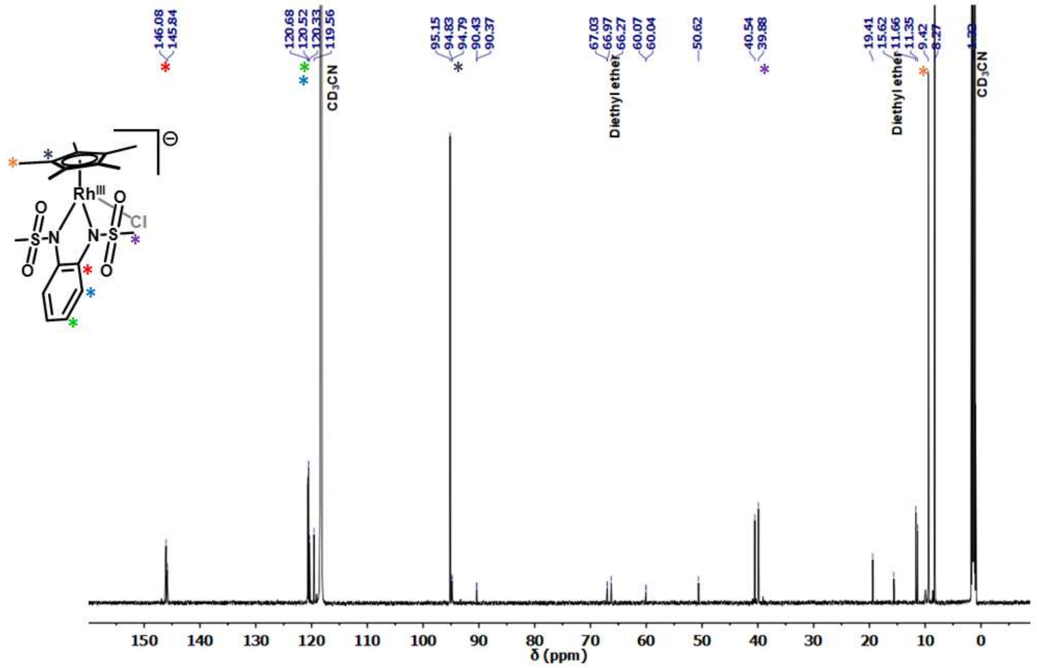

Figure S30: ^{13}C-NMR spectrum of the product mixture after reaction with DCM. Assignments given for **[1-Cl]$^-$**

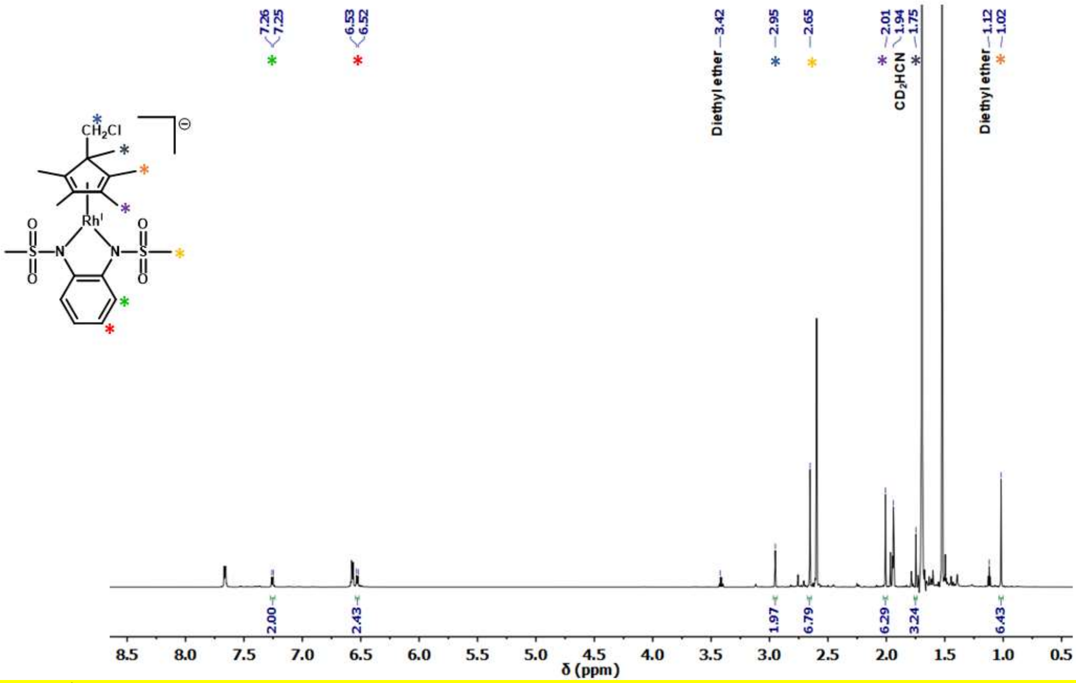

Figure S31: ^{1}H-NMR spectrum of the product mixture after reaction with DCM. Assignments given for **[1-CH$_2$Cl]$^-$.**

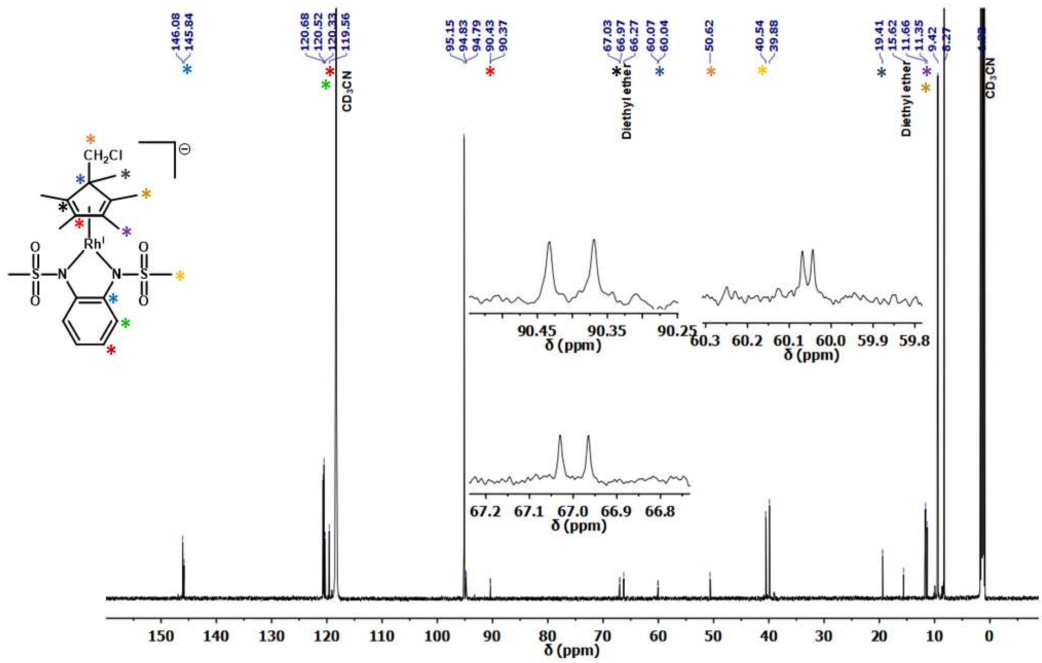

Figure S32: ^{13}C-NMR spectrum of the product mixture after reaction with DCM. Assignments given for **[1-CH$_2$Cl]$^-$.**

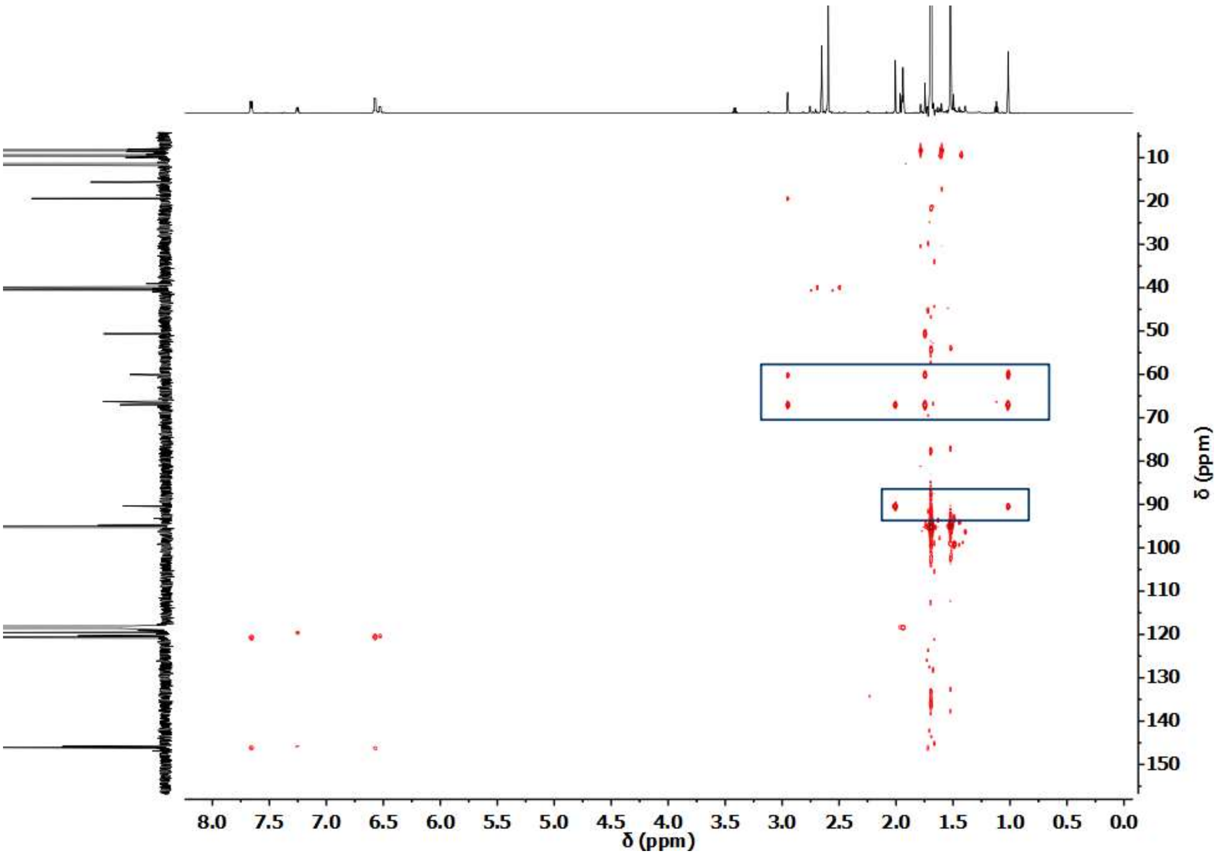

Figure S33: ^{1}H-^{13}C-HMBC of the product mixture after reaction with DCM. The blue rectangles highlight the coupling between the doublet signals of the ring carbons and the proton signals of the substituents on the cyclopentadiene ring.

c) NMR monitoring of the addition of $[CH_3]^+$ to [3]

A Young NMR tube was charged with **[3]**, $[Me_3O][BF_4]$ and DCM-d_2. NMR spectra were recorded approximately every 24 h to detect the intermediate formation of **$[3\text{-}CH_3]$**. The appearance of a doublet at low-field (δ = 0.75 ppm, J = 2 Hz) (see inset in figure S27) is indicative of a Rh-bound methyl group. At the same time, the formation of the binuclear complex $\mathbf{[5]^{2+}}$ is visible. Characteristic signals of the substrate **[3]***, the binuclear reaction product $\mathbf{[5]^{2+}}$* and the putative intermediate $\mathbf{[3\text{-}CH_3]^{+}}$* are marked with asterisks of different color. The intense signal at δ = 3.25 ppm corresponds to the side product dimethyl ether.

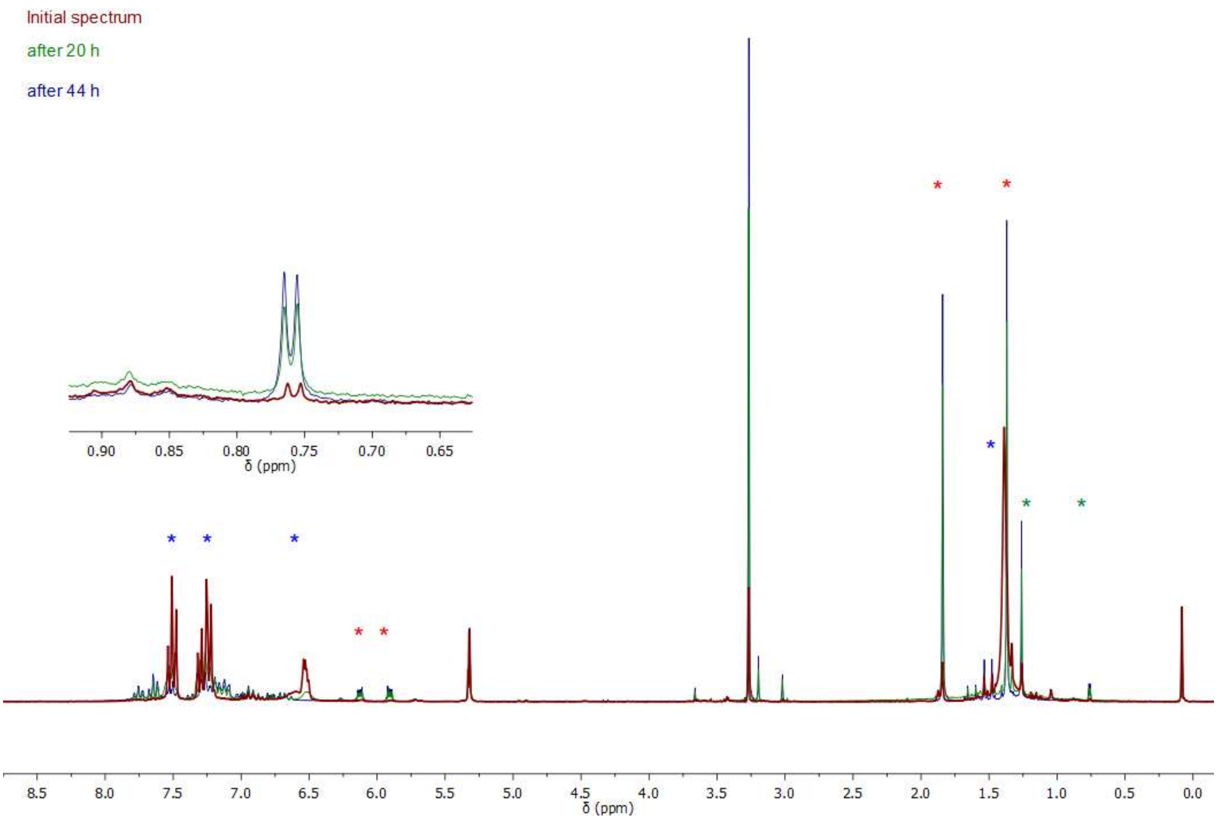

Figure S34: NMR monitoring of the reaction of **[3]** with Meerwein's salt.

DFT calculations

HOMOs and LUMOs of neutral complexes

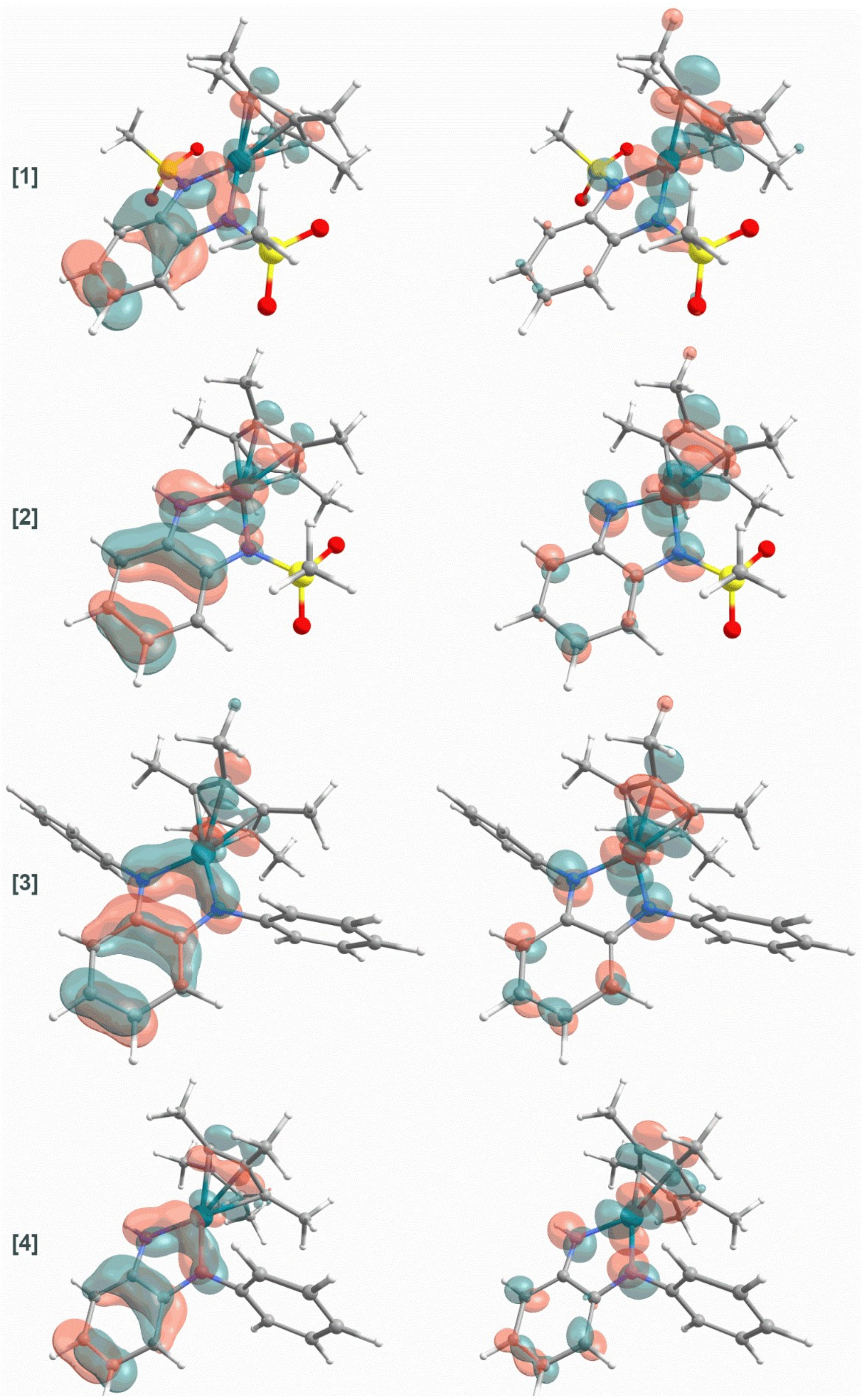

Figure S35: HOMOs (left) and LUMOs (right) of complexes **[1]** - **[4]**, calculated at the UKS-TPSSh/def2-TZVP level of theory. Contour values 0.042.

Spin densities of anionic Rh(II) complexes

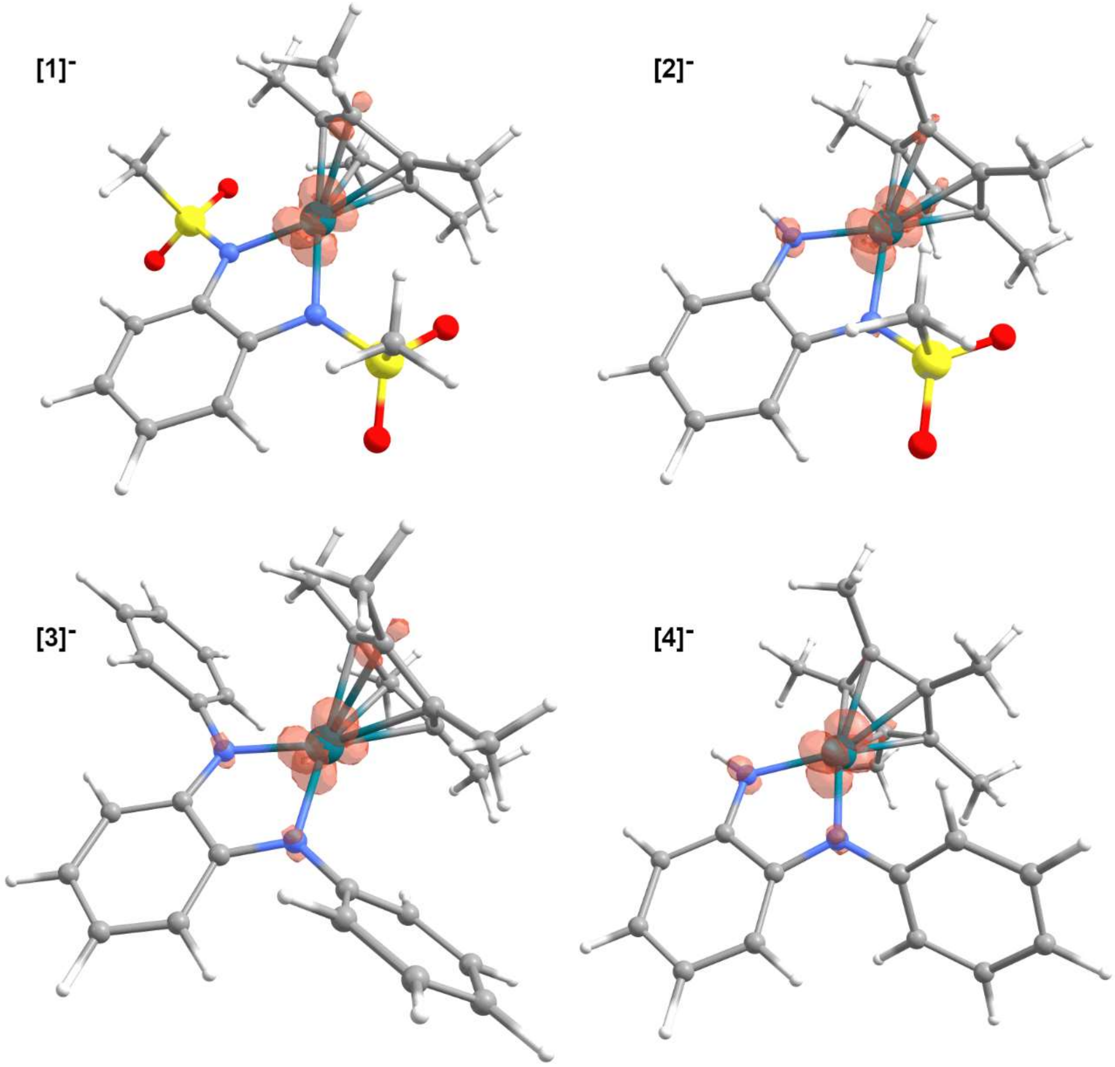

Figure S36: Spin densities of the anionic Rh species [1]- - [4]- at UKS-TPSSh/def2-TZVP level. Contour value 0.015.

Model input for geometry optimization and frequency calculation

!RKS BP86 RI D3 TightSCF Tightopt ZORA ZORA-def2-TZVP SARC/J GRID6 NOFINALGRID

%pal
nprocs 8
end

!Opt
!NumFreq

```
%basis
NewGTO Rh "old-ZORA-TZVP" end
end

%rel
soctype 3
socflags 1,3,3,1
method zora
modelpot 1,1,1,1
ModelDens rhoZORA
onecenter true
end

%scf MaxIter 5000
end

%geom MaxIter 5000
end

*xyzfile 0 1 Rh_NSO2Me_opt_freq.xyz
```

Model input for UKS single-point calculation

```
!UKS TPSSh RIJCOSX D3 TightSCF ZORA ZORA-def2-TZVP SARC/J GRID6 GRIDX6 NOFINALGRID NOFINALGRIDX SmallPrint

%pal
nprocs 12
end

%maxcore 3000

%basis
NewGTO Rh "old-ZORA-TZVP" end
end

%rel
soctype 3
socflags 1,3,3,1
method zora
modelpot 1,1,1,1
ModelDens rhoZORA
onecenter true
```

end

%scf
MaxIter 5000
end

%output
Print [P_Basis]2
Print[P_MOs]1
end

*xyzfile 0 1 Rh_NSO2MeNPh_sp_TPSSh.xyz

Coordinates of minimum structures

[Cp*Rh(bmsab)] ([1]):

52

Rh	7.58800702056010	4.34870237026790	2.77681236410441
C	9.75666540471850	5.05353941478645	4.63866475849224
N	8.68852693624696	5.61950517825485	3.92619337384879
O	7.10905986629609	7.55507884980007	3.51214508796730
S	8.42785955139205	7.27266511118763	4.05200980850421
O	8.73472831571000	7.74809977260786	5.39082122476838
C	10.79975812019701	5.73917673346644	5.29366870681842
H	10.79449736545612	6.82349180813999	5.34539194905561
C	5.63734980155314	3.63691474514484	2.03071928687349
C	6.66293411288422	3.17286944765971	1.14438268263276
C	7.35442402974384	4.33204873908630	0.62842469930867
C	9.63745469678012	7.97009534774828	2.92466739493091
H	9.41442526317729	7.61530411753763	1.91359214330054
H	10.63774437082051	7.64986683743770	3.23759281327816
H	9.54227770094431	9.06074472760539	2.98664971935770
C	11.81759959526909	5.03311419813751	5.92264336352176
H	12.61897581985855	5.58098290770193	6.41944002361850
C	4.61780710375805	2.81288137176114	2.73757390985408
H	3.68580186930513	2.79104347149440	2.14731893135057

H	4.37584249362916	3.23971864937761	3.71938853851159
H	4.96668977199153	1.78858176342469	2.89192505197362
C	6.87591274094796	1.77932132004720	0.65922475843579
H	6.25421452893326	1.61518092830958	-0.23666408440435
H	6.60716346065216	1.03747574245067	1.41553410393906
H	7.91873462258535	1.61077279368470	0.35912289543151
C	8.47151403096228	4.30960582187441	-0.35846036053747
C	9.74492654543852	3.63136404145821	4.64748666584939
N	8.66704766727768	3.07429494565319	3.94266712820280
O	7.05316387059796	1.16109297906512	3.55749813616598
S	8.37763756218258	1.42785255438196	4.09139187519721
O	8.67852252014342	0.96522292529786	5.43602051931346
C	10.77714250695835	2.93680658258744	5.31024252559386
H	10.75459276650161	1.85339541913348	5.37458165902464
C	9.57282183761467	0.69387338907816	2.97189181912548
H	9.35530028829953	1.03969417843775	1.95651750309748
H	10.57919848152279	0.99963650524747	3.27974508371004
H	9.45769975420333	-0.39395977200598	3.04791364376589
C	11.80641002564166	3.63373911793637	5.93074851370144
H	12.59906237205382	3.07891659979083	6.43378390168570
C	5.65406196002668	5.09230582769772	2.01748028658820
C	6.69072135103228	5.51632038895333	1.12397669556655
C	4.65209854356922	5.95112952723986	2.70808495348637
H	3.70942606984283	5.95307167026451	2.13473303939334
H	4.42645191826608	5.56767181061478	3.71153648137888
H	5.00843521976424	6.97884712276376	2.81375590297726
C	6.93843412109030	6.89611600235628	0.61645146219124
H	6.31656369200511	7.06399168785339	-0.27859290360009
H	6.69362062468133	7.65608092304375	1.36301394085238
H	7.98386050637569	7.03145714036634	0.30863650931971
H	9.09393247994877	3.41416962210826	-0.23878309093213
H	8.07580569311409	4.30669383227168	-1.38843654493975
H	9.11800302747464	5.18966980940954	-0.25303785165159

[Cp*Rh(bmsab)]$^{-}$ ([1]$^{-}$):

52

Rh	-0.38418366215506	9.13020516634507	5.81204565243003
S	0.75035498617500	6.12181397116038	6.61555101999253
O	1.88886493352371	12.43675126867366	7.92450172281727
C	-2.48337433198976	9.94712869878169	5.76630678209619
O	-0.68451583884720	5.85590347815100	6.64419523536032
C	-1.74972575053238	10.40185024168190	4.59991312867658
C	-1.63424162135037	11.82379704230947	4.15417705731228
H	-2.57226409727741	12.14121492934808	3.66453384616676
H	-0.82905871970062	11.94949399222188	3.41594924320752
H	-1.44009521439389	12.49359035386601	4.99913943468325
C	1.35591024977343	5.51164077317116	5.02826783825338
H	2.41305436391461	5.78919825328150	4.93597006568700
H	0.76522428429842	5.98116808239239	4.23549778882732
H	1.23724011363972	4.42121251487343	5.01683732893745
O	1.55917326420241	5.46563864090210	7.64340519038130
C	1.69372183399468	12.61696026781229	5.31436739294143
H	1.06432692049120	12.26965833474705	4.48905519427361
H	2.71989772313811	12.24764733323176	5.19725749016693
H	1.67969827109430	13.71122542827040	5.38924584036430
O	-0.37725953257545	12.34114162172590	6.90480919458012
C	-3.28901150530914	7.62854293363178	6.65092823207779
H	-4.29233236624829	7.37971137488727	6.25827310063829
H	-3.42570920400097	8.11307121139697	7.62735319136157
H	-2.73735392371248	6.69543224355128	6.81371683503593
C	2.35838158326058	9.67412467591204	6.81287820086428
C	3.47954724732075	7.50874441935661	6.88134770775200
H	3.43370647139343	6.42332049374074	6.89860192439872
C	3.61075380660123	10.28835624296162	6.99619886806285
H	3.66702680713462	11.36813341911238	7.10308881661996

C	4.78015974183907	9.53136522205332	7.10221937819086
H	5.73611233437122	10.04070636329090	7.24117185708215
C	-0.76056018197435	9.28308161252857	2.45329822382311
H	-1.50265423734946	9.34959939758457	1.63477067371142
H	-0.16484384379028	8.37539811522545	2.28285943684075
H	-0.08295411607915	10.14239235833647	2.35218955300636
C	-1.41239354293375	9.26024390182377	3.79731863294991
C	4.71457167129405	8.14109645598634	7.04462698061059
H	5.61786645245208	7.53444241190089	7.13738978434996
C	-3.13728886431580	10.82714726126749	6.78016534016121
H	-4.11407452340679	11.19514564787906	6.41519420342058
H	-2.50351801499245	11.69113820778884	7.01099923142814
H	-3.31699722984143	10.28247014042013	7.71712236016319
C	2.29120720631093	8.25101002345840	6.75435147948866
N	0.99477513586585	7.73313449884182	6.54373875686317
S	1.02608610359058	11.94367161364115	6.85037118809537
N	1.11732886030831	10.32722749226614	6.64978142196456
C	-1.85938884880765	8.09497111302085	4.50679437478132
C	-1.87737494463166	6.70859589315755	3.94833848935565
H	-2.83994900078465	6.52114361922948	3.43971430838959
H	-1.74938471786167	5.95826399190368	4.73641708605095
H	-1.08583685409295	6.56692851750713	3.19832626297185
C	-2.55117067703322	8.52175273338958	5.70874665233336

[Cp*Rh(bmsab)MeCN]$^+$ ([1·MeCN]$^+$):

58

Rh	2.88764786416090	5.70914798231649	3.70266097339279
N	2.82806947458618	4.16857078891873	5.10572001667388
N	1.93967889322497	4.55464968564547	2.28439413070869
C	4.21339314022264	7.12401376461362	4.62041415227536
N	1.10401235450036	6.03179484518267	4.73318999986474
C	3.46604664323667	7.83973521123360	3.59923478409303

C	3.72444853805263	7.21460843101517	2.33799428608305
C	4.70844350133937	6.15206025251286	2.55450574517655
C	5.04204065216623	6.13796716519435	3.94640972887560
S	3.71840575991325	2.74680829877991	4.92085135997481
O	4.57628421031096	2.55134874214617	6.07750392778645
O	4.29721043039169	2.82279447823710	3.59074904522066
C	2.46601121483395	1.46972100647424	4.90177929318041
C	2.00934136648844	4.39142510290661	6.17816003818987
C	2.01766278205551	3.69034548006691	7.40805304508129
C	1.03304482520945	5.44669157360766	5.96729558629393
S	-0.16247612938406	6.93678512001199	4.08195363682203
O	0.22600240061171	7.21548237007462	2.71019705805735
O	-0.49684529715206	8.02978556746985	4.97946528488515
C	-1.52548536554696	5.77871999808642	4.04108717153044
C	0.11013406976507	5.75296678019469	6.99611646388313
C	0.14095985319529	5.03711289818691	8.17892478375219
H	-0.57917328711350	6.58513550069498	6.86446035536633
C	1.47047483968444	3.96561894477102	1.40519449752806
C	1.09143175136000	4.00920201761205	8.38412941053648
H	-0.55595394920955	5.29216608290536	8.97733993237152
H	1.11676292035818	3.48299658635850	9.33842426655477
H	2.78828393228717	2.94373112291534	7.59154870970450
H	1.81436801626357	1.64089014205897	4.03890702016499
H	1.90179553464539	1.49161645193120	5.84041519545124
H	3.00588814338038	0.52014088557793	4.80207047690780
H	-2.38059709263459	6.33439051324923	3.63723013843682
H	-1.74287948986375	5.42765976659036	5.05564858531747
H	-1.25168558778920	4.94927446474440	3.38111517108327
C	4.25181838710185	7.45633777482115	6.07323087114780
C	2.61719807363840	9.03631003099663	3.83621343211584
C	3.15081331485483	7.62672949197792	1.02682598605597
C	5.31667168684195	5.29108024619424	1.50253510413402
C	6.07975967063918	5.29869666932086	4.59991031639304
H	5.02560235986013	8.21442650646134	6.27327886507426

H	4.48739124351148	6.57226458544880	6.67820382798145
H	3.29165668723151	7.86223819715737	6.41440906697656
H	3.26936528126149	9.92520260752388	3.85839713551590
H	2.09199856823569	8.98817800903337	4.79728109910574
H	1.87384046343673	9.17366315643911	3.04538318167313
H	3.74706849556923	8.44668887061928	0.59590094186831
H	2.11752909780165	7.97385959601648	1.14271690598617
H	3.16065577665204	6.79880240112152	0.30794914655137
H	4.64961006293409	5.18284806509773	0.63897127153125
H	5.54138621192099	4.29235124618494	1.89420946482552
H	6.25446171295720	5.74300168074476	1.14206971214434
H	6.30268102670725	4.39756022303509	4.02104461962418
H	5.78949996983446	4.99400006292490	5.61220889406137
H	7.00387684895792	5.89432864101940	4.68488576448404
C	0.89179598722625	3.23616056611805	0.29639946229012
H	0.42678241653186	3.93657833859447	-0.41128504064748
H	0.12473200885862	2.53878013944206	0.66028169746395
H	1.67206773388438	2.66336487142098	-0.22386599758166

[Cp*Rh(amsab)] ([2]):

46

Rh	9.33560344153890	4.67135014068745	4.72812734647555
H	10.73801787156951	2.46493276423173	5.30771316363196
N	10.30708363073375	5.89566076155439	6.06400212847243
S	9.86169083017183	7.46560239057506	6.42444336219057
C	11.32585196790471	5.29645067997856	6.81206431368144
C	11.48264107186061	3.91211687364871	6.49878627961621
N	10.63309397199511	3.45336131358026	5.53882517019246
C	12.45160895010727	3.13491674998780	7.16687803088668
H	12.55491962270789	2.07792809195914	6.91043744058912
C	13.25547952732114	3.71740326963305	8.13564852089752

C	13.10889053771016	5.08193143585579	8.44168155023445
C	12.16240700844215	5.86922364952337	7.79085800987080
H	12.10066084953028	6.93287199971125	8.00696224473078
C	9.06086875890721	7.29405576993959	8.02212947237342
H	8.18143247568288	6.65417636948673	7.89397898488389
H	8.77153532684905	8.30198325040192	8.34217577840926
H	9.76947023497518	6.85083438144050	8.73118763028199
O	8.83748705091833	7.86662702094606	5.47296482022982
C	8.23927940660152	5.72596854409030	3.10099776206003
O	11.02432804425407	8.32691996653847	6.58846634083132
C	7.29952004652027	5.30896222844599	4.10119245195984
C	7.38795956445322	3.86727274285927	4.22634758080208
C	8.37891043649932	3.39544589448185	3.28815949968790
C	8.91435745669217	4.54657359626445	2.60123119757146
C	8.42371200051328	7.10470498288319	2.56167109109024
H	9.47699808636820	7.30457274945354	2.32552932992955
H	8.10089333191757	7.86145770820102	3.28158897397911
H	7.84277167730101	7.22446237800754	1.63164405874848
C	6.32063157581982	6.17851202475193	4.81570486547353
H	6.75177487497995	7.16314237349501	5.02565892118451
H	6.01097321196250	5.73001285170730	5.76900007283364
H	5.41318523337779	6.31462717355296	4.20399725751475
C	6.53513443880396	3.01521388088295	5.10596928723096
H	5.58487507955998	2.77015762795939	4.60201141195637
H	6.29061202935833	3.52882877019293	6.04434414750628
H	7.03309377741097	2.07067400696678	5.35781076152606
C	8.74970240388783	1.96926326064418	3.03995048278198
H	8.11017832940573	1.52747655907048	2.25915998749448
H	8.63034338784942	1.35937232735456	3.94563769502536
H	9.79116252057716	1.87862779352903	2.70372042486986
C	9.92838970751033	4.53016105335335	1.50715385683681
H	9.43093545138349	4.50107323426978	0.52274602953319
H	10.58475156199466	3.65383806997186	1.57627385290278
H	10.55706244449968	5.42895501871481	1.53479673771174

H	14.00584909981091	3.11859378846605	8.65329240991132
H	13.75392169176028	5.54140248074942	9.19153926339775

[Cp*Rh(amsab)]$^-$ ([2]$^-$):

46

Rh	9.40433989374553	4.73265720562669	4.74410507843980
H	10.78048538160171	2.49164705902620	5.40470046154953
N	10.49663182185179	6.02875947332385	6.01170952503220
S	9.82202396475691	7.40637870068821	6.55434218821456
C	11.48724679942615	5.37345627363896	6.79162039418928
C	11.58169109221212	3.97059967463864	6.51001830794853
N	10.69975615990673	3.48961036376379	5.59347191671441
C	12.54902131564128	3.19431118801328	7.18733140984995
H	12.61051123008351	2.12481044550575	6.96280810311456
C	13.39775247319444	3.77214747436136	8.13155850259349
C	13.30077743825334	5.13975635820078	8.40871905968759
C	12.35659316813761	5.93365884132060	7.73831257406179
H	12.29935656381239	6.99985001417228	7.94160980072179
C	8.70840976199255	6.85108290098898	7.86432439964140
H	7.98442389489961	6.16349380487600	7.41340182095697
H	8.21605815679646	7.73193568174585	8.29443629034440
H	9.31547638883410	6.33454470819960	8.61834411156161
O	8.98214737227486	7.98050168790541	5.50381453707463
C	8.27551974763211	5.74001654105220	3.05261887297982
O	10.77504249642637	8.32275525182184	7.18896925576252
C	7.33073126211831	5.38362437663370	4.09463115166723
C	7.36028928527002	3.96252701989266	4.25929159520980
C	8.37189196857857	3.44547633529884	3.35784477321559
C	8.87528320926662	4.53999365077398	2.55539727584354
C	8.48083488727350	7.11783558834326	2.51016894327493
H	9.43500415688339	7.19148589450530	1.96944431422841
H	8.50433842087199	7.85298843820022	3.32350582860707

H 7.67526706072677 7.39568416031653 1.80573928930286
C 6.39319396535022 6.33282133388763 4.76867515157858
H 6.90605458588614 7.26978922793459 5.02083706251810
H 5.99458591760534 5.90431172220401 5.70022115089723
H 5.52914181467322 6.57208096612828 4.12159412358317
C 6.48580917784581 3.14657954782723 5.15577795094418
H 5.57725159445411 2.78821992544203 4.63455807633336
H 6.15715171032875 3.72887278983402 6.02828056817762
H 7.01681008598549 2.26207796812722 5.53562061218138
C 8.71074998518295 1.99954948563746 3.17163057055816
H 8.05252613635546 1.51559298512028 2.42830349821653
H 8.60702162455480 1.44359891965977 4.11493236599839
H 9.74741541671651 1.87645266784448 2.82601346028313
C 9.84146037287224 4.42706893697703 1.42107586674433
H 9.32878255105256 4.25273065102757 0.45545383658775
H 10.54337586229423 3.59483705498536 1.57451765867321
H 10.43953187625795 5.34312366360208 1.31648063507487
H 14.13708874775967 3.15427374641391 8.64785819229834
H 13.96519320235479 5.60412929451181 9.14038943756293

[Cp*Rh(bphab)] ([3]):

60

Rh 6.62158394588732 15.88776138115761 3.17054327682779
N 8.51804742606887 15.57652347030687 3.69413153033502
N 6.65180723557780 16.63015166796853 5.01923259299754
C 7.82370707310960 16.52637456103205 5.71694342505759
C 3.31217444598857 15.87141856406393 3.43420582833279
H 3.32368157159713 15.16159953903997 4.27085798563026
H 2.39156279876523 15.69344612365972 2.85443911903220
H 3.25109996612838 16.87908679981907 3.86342607328272
C 4.76579944795859 16.37129596376423 6.55612475382046
H 5.13188517375460 15.37456386887297 6.80586270478192

C	8.06283764148494	16.96332843177548	7.03715902171522
H	7.25643761888838	17.42912117343734	7.60380117868846
C	6.24932310581330	14.74001218305568	1.33331601219556
C	5.49636234365502	17.14791550930009	5.64445211179567
C	10.02411193563600	15.62453887573922	1.78085017170963
H	9.79150504427887	16.68371205793154	1.66705422752136
C	9.73707802071199	13.56492195471264	3.02060804262789
H	9.27659958520917	13.03139609890036	3.85316901400261
C	8.88354224192022	15.92829743422186	4.96436343181578
C	5.14465965112270	16.74799256944580	1.78880940018260
C	5.03991164261069	18.43169832411974	5.31477569279783
H	5.62707751646097	19.02650430740343	4.61456221175643
C	9.43889007968643	14.92439252562201	2.84500037989401
C	4.51539687852482	15.71292094099881	2.56516535376290
C	5.20299134617455	14.47172408921349	2.29298178876441
C	6.21894274204515	16.14520791901906	1.02557285568069
C	10.15960707152532	15.78006364566377	5.54806981251451
H	10.96787845889027	15.33448440852187	4.96798234406964
C	3.58699354686679	16.86749066778926	7.11364410091928
H	3.02075254729922	16.25268557170088	7.81530148755923
C	10.88040050875157	14.96980245864054	0.89413088721215
H	11.33010225353399	15.52363294746155	0.06821449935297
C	10.36923100587888	16.20926672216984	6.85258962673726
H	11.35522605758489	16.09432851807603	7.30494287881529
C	9.32474120432814	16.79871340276318	7.59434940078605
H	9.51017483947952	17.13538383115727	8.61527446145864
C	4.84233081987487	13.13266474436782	2.84451076835923
H	5.72044525404019	12.47765236863196	2.90948560009707
H	4.09916136871075	12.63216947053190	2.20016421788530
H	4.40745336535724	13.21857590078049	3.84848796255473
C	11.16449972081069	13.61132541760295	1.06283994930023
H	11.83011813624772	13.09929768590930	0.36701731250216
C	3.85995869783301	18.92421666124075	5.87451561737460
H	3.51271046193719	19.92466646972095	5.61065799384760

C	10.59385292567471	12.91430375293107	2.13218325772474
H	10.81305878731759	11.85436044216513	2.27153859036750
C	3.12611048154672	18.14252229959855	6.77175328480565
H	2.20185089910887	18.52620953486234	7.20526015373663
C	7.14417299535785	13.72059668484628	0.71029825302329
H	8.10865102010028	14.15227660153969	0.41550880317063
H	6.67389945922731	13.29396403704525	-0.19089283333339
H	7.35512216554727	12.89466261101683	1.40112131600973
C	7.07500993717627	16.84205487602643	0.02076347791462
H	7.27293624482241	17.88173247744048	0.31351357231746
H	6.58153675515568	16.86138879361041	-0.96579702921323
H	8.03940313747099	16.33298345949427	-0.09947741526123
C	4.70451455839407	18.17178196302122	1.70396059349998
H	4.19436014403104	18.48480158576593	2.62347521357999
H	4.00064985753591	18.31297929741068	0.86621305902558
H	5.55535083352310	18.84634235591262	1.54001359627652

[Cp*Rh(bphab)]⁻ ([3]⁻):

60

Rh	6.68944173905142	16.04617434318619	3.14139746060589
N	8.60645490061776	15.64882761749899	3.70930135428359
N	6.69951065374896	16.72289306267167	5.06434942405550
C	7.80953632714641	16.38595816607418	5.81175663087776
C	3.37092371152859	15.90717463127552	3.46797720630915
H	3.46598918407085	15.28981857391369	4.37212965308897
H	2.44373356437328	15.60527928563197	2.94919511931017
H	3.24760197567195	16.94351710885051	3.80441564629341
C	4.89098646356497	16.60759430339522	6.73466796048547
H	5.31615811321662	15.68881582454687	7.14058034507086
C	8.01516810018821	16.66762157595784	7.17683618809804
H	7.22357137833982	17.15878341426132	7.74364721901629
C	6.27267559277742	14.78582344583013	1.35674496884603

C	5.55272374311083	17.23818182795521	5.65599883466776
C	9.87949968817767	15.65926006059617	1.65411858575008
H	9.48374638187025	16.66002051465752	1.47702484980514
C	10.01393909782509	13.72171671001711	3.09370395175071
H	9.71963779212791	13.20677138467363	4.00908208274743
C	8.87272110153432	15.78715633823129	5.05624172548253
C	5.11299337491575	16.77272396173123	1.70755260878987
C	4.97712536125725	18.41765516904081	5.13431098905950
H	5.49566045159172	18.90230278964559	4.30623613695507
C	9.49899028919588	15.01620904722207	2.85253419242528
C	4.55905084792243	15.75076460997542	2.57339481434276
C	5.23017335259395	14.50867193440107	2.30609087698001
C	6.17524706889466	16.17472675817350	0.95362262013352
C	10.09146185701555	15.49813776876835	5.70138353008727
H	10.91439380704058	15.07985914375091	5.12089591047064
C	3.70308707979345	17.12616930241753	7.24689247742867
H	3.19982133692825	16.60497603038716	8.06546322827248
C	10.72109399174321	15.03671508364659	0.73574949816958
H	10.99908072234180	15.56230071237735	-0.18145415154899
C	10.26208595776515	15.76209851263268	7.06454952676052
H	11.21321922856617	15.52168885688894	7.54524880368241
C	9.22490975660597	16.34632143171653	7.80159309363973
H	9.35845395942530	16.56648016478281	8.86329848310780
C	4.91076471338487	13.18035132771507	2.91049211739910
H	5.80978628418367	12.55275419539900	2.98866328181975
H	4.16765869083073	12.61882085269705	2.31154287778444
H	4.49723434969262	13.29348947817573	3.92266446016279
C	11.21296651893484	13.74750805283309	0.97938608588273
H	11.86795511868524	13.25632917381021	0.25773169290049
C	3.79355712320547	18.93522581961275	5.65435915078342
H	3.37502250986554	19.85157832637005	5.23019828297405
C	10.85049103777759	13.10059515041632	2.16810184863960
H	11.21489700881023	12.08972382131278	2.36889987272253
C	3.13939613168927	18.29286408380460	6.71401575519970

H	2.20874455484797	18.69441559423572	7.11861353355507
C	7.18306000944300	13.75979184287745	0.76102858083240
H	8.09877119341183	14.21035040841895	0.35981201585093
H	6.68236698785751	13.21770014853080	-0.06080085363629
H	7.49590616664378	13.01909959370667	1.51012861325362
C	6.95276128198594	16.82389957433399	-0.14550918971142
H	7.09674530576734	17.89739881715340	0.04587954858312
H	6.44064898295504	16.72585174738171	-1.12135962103744
H	7.94626684065289	16.36750241310385	-0.25158471833172
C	4.57998265847327	18.15879145044080	1.53729300257915
H	4.10784080452499	18.51871418624148	2.46151749906323
H	3.81597890503535	18.20710609279466	0.73846843700365
H	5.37961086880088	18.86623638184848	1.27294888042573

[Cp*Rh(aphab)] ([4]):

50

Rh	2.63261617787145	0.37941546178963	1.30862922488220
C	0.70877039656531	1.51223494375490	-1.13077727989198
H	1.12459197465606	0.85105604017140	-1.90160270180424
H	0.37866493150166	2.43961068494991	-1.62923094333448
H	-0.18136089456325	1.02273545622258	-0.71470672264848
N	2.42055617009514	-0.79555118236009	2.90739838212613
N	3.12648968388126	-1.39178503528131	0.57602934703976
C	3.89809911100936	1.55584943882478	-1.48097799606041
H	4.88716023648895	1.13020742712581	-1.26574405971963
H	4.05160356838151	2.47553512933940	-2.06887739716931
H	3.35723384742424	0.84456853698724	-2.11942485096213
C	2.66368351133267	-3.12582376494361	3.74373544832868
H	2.35291507987636	-2.86785696240261	4.75642164079559
C	2.64478975313143	2.47549944795972	1.97236384955209
C	1.40494352264377	2.20208205883297	1.29564229249104
C	3.14339071079633	1.84199700924864	-0.22265938675937

C	3.72333942257029	2.25386471856548	1.03610594322469
C	2.72422580439695	-2.12054823610077	2.75548010698690
C	1.71185559493435	1.81098435575519	-0.06540693830581
C	3.11973426191844	-2.45614649865553	1.41993458591405
C	2.08869313397014	-0.27891630815139	4.17886096106130
C	0.03663378755304	2.38808710087242	1.86277407835934
H	-0.67455629408705	1.66406889142121	1.44339807485126
H	-0.34393801623070	3.39878327493289	1.63789411332358
H	0.03976765411897	2.26798164284689	2.95315413728153
C	2.78893693262765	2.96842535517661	3.37451000926455
H	1.97702336267536	2.60344809921939	4.01610293315458
H	2.77440742182035	4.07040544740626	3.40322089268987
H	3.73296397606260	2.63363688953358	3.82277923541445
C	3.39162285742422	-4.76528301399611	2.09618044058514
H	3.64900916651110	-5.79751763421488	1.85492635174785
C	1.43958194828101	0.89181981602241	6.64637432035406
H	1.18878566738843	1.35319563347739	7.60221300054251
C	0.76180581828815	0.06549358980629	4.47197354378623
H	-0.00345226726416	-0.13240288867387	3.72105627938001
C	2.76140830024982	0.53414982337527	6.36309616903898
H	3.54573225189851	0.71699001260002	7.09952431043247
C	0.44101777734581	0.65034980064216	5.69817296380786
H	-0.59487646442167	0.91611002414264	5.91593548570876
C	5.17386838699635	2.48334617949041	1.30351705400320
H	5.42014497319520	2.29097683387824	2.35554206298773
H	5.45135075330411	3.52807631282399	1.08098641701536
H	5.80453484503268	1.83209892064509	0.68514814830652
C	3.08646986237210	-0.04927141522372	5.13833396074956
H	4.11491563857676	-0.32213966952010	4.89834876494646
C	2.99698857566653	-4.43247706859283	3.40988923516537
H	2.95009292187016	-5.21145709274110	4.17202755226170
C	3.45246133190006	-3.79172567251062	1.10803553985358
H	3.75442418729647	-4.04599765023812	0.08919489543126

H	3.42322864466551	-1.59683426423414	-0.37826347619051

[Cp*Rh(aphab)]$^{-}$ ([4]$^{-}$):

50

Rh	2.49935331609977	0.34148191868359	1.31079822495380
C	0.55088776422230	1.67473530316635	-1.10029331455186
H	0.86581100903268	0.91895712266768	-1.83389210737033
H	0.32265129802620	2.60277576583659	-1.66047338070535
H	-0.38854012494170	1.32112862454981	-0.65132125260794
N	2.29177547023615	-0.88767789088539	2.94763897012203
N	3.15402274229696	-1.41023818526276	0.59713893628948
C	3.71491911144446	1.40028857884301	-1.51960439811799
H	4.67864440365469	0.90893407907353	-1.32107873626345
H	3.91569858542270	2.27999797942046	-2.15727000659846
H	3.10172088548409	0.70056734960084	-2.10578793134948
C	2.79899768278860	-3.19833102153045	3.75281311289393
H	2.39204218935057	-3.00589423410346	4.74660645691505
C	2.66998090235679	2.49533486427475	1.92899799071015
C	1.37766436351865	2.30436646685287	1.29597936907667
C	3.02983363221975	1.77831083787782	-0.24340381517636
C	3.69208091136878	2.23398465989649	0.96044593331204
C	2.79380356448790	-2.16799384111646	2.79530994818001
C	1.59986735565046	1.89038857558008	-0.05731997928584
C	3.27466802178364	-2.44696284169644	1.46926518970983
C	1.96509830082921	-0.37844508991560	4.19446813305385
C	0.05648776266102	2.66974944395056	1.89372979809516
H	-0.74856566424663	2.01451035665992	1.52858378154340
H	-0.22436996883623	3.71012072214172	1.64396247309298
H	0.07965133745156	2.59180833655454	2.98845777708087
C	2.88699252543318	2.98176907047463	3.32693304020952
H	2.12475228724883	2.58590567535910	4.01143739596371
H	2.85509439813068	4.08508968195942	3.38610879918929

H	3.86256476289907	2.65428685712887	3.71260819199914
C	3.81679377543554	-4.72709458053588	2.16103720147481
H	4.22499686154726	-5.71208144154525	1.92068501099017
C	1.28210015668868	0.84037274583876	6.67255744776636
H	1.02429649529247	1.31232687287030	7.62227566674711
C	0.75008699740712	0.32576215290388	4.36028158785490
H	0.08624776252951	0.38177147145743	3.49769905428228
C	2.48448331410532	0.13298175999716	6.52807824087244
H	3.17861068893671	0.06592311628828	7.37033248387238
C	0.41719278755534	0.92350054029418	5.57282437746649
H	-0.53186096977522	1.45875465234903	5.66455509049559
C	5.16638044690292	2.38206367451146	1.15421377832391
H	5.44365254938574	2.24369214557531	2.20894691080805
H	5.53031116694958	3.38098424661385	0.84391101735455
H	5.72340793481827	1.63471808241458	0.57077261241628
C	2.82079030146701	-0.47204955964033	5.31969451962593
H	3.76726433163955	-1.00269883340926	5.21032856309138
C	3.31149825834003	-4.46614677335910	3.44203570519608
H	3.31142657297032	-5.24904967839272	4.20392849338982
C	3.79582010773695	-3.72686223123633	1.18312787815741
H	4.17503609812536	-3.92831890155073	0.17607506891240
H	3.55022553586706	-1.57614862748670	-0.32695930946245

Crystallographic Details

Table S7: Crystallographic details of complexes **[1]**, **[1·MeCN]** and **[2]**.

	**[Cp*Rh(bmsab)]**	**[Cp*Rh(bmsab)·MeCN]**	**[Cp*Rh(amsab)]**
Chemical formula	$C_{18}H_{25}N_2O_4RhS_2$	$C_{20}H_{28}N_3O_4RhS_2$	$C_{17}H_{23}N_2O_2RhS$
M_r	500.43	541.48	422.34
Crystal system	Orthorhombic	Monoclinic	Orthorhombic
Space group	Pnma	$P2_1/c$	Pbca
a (Å)	13.613(2)	13.601(3)	13.266(3)
b (Å)	17.390(2)	11.169(2)	15.322(3)
c (Å)	7.941(1)	15.316(2)	16.848(3)
□□(°)	90	90	90
β (°)	90	103.4(1)	90
□ (°)	90	90	90
V ($Å^3$)	1879.8(3)	2262.8(4)	3424(2)
Z	4	4	8
Densitiy (g cm^{-3})	1.768	1.589	1.638
F(000)	1024	1112	1728
Radiation Type	MoK□	MoK□	MoK□
μ (mm^{-1})	1.159	0.971	1.130
Crystal size	0.38 x 0.17 x 0.12	0.26 x 0.26 x 0.15	0.2 x 0.18 x 0.17
Meas. Refl.	55638	54654	55917
Indep. Refl.	2410	9395	3510
Obsvd. [$I > 2\sigma(I)$] refl.	2302	8325	3000
R_{int}	0.0307	0.0384	0.0524
R [$F^2 > 2\sigma(F^2)$], w$R(F^2)$, S	0.0182, 0.0492, 1.079	0.0255, 0.0618, 1.058	0.0218, 0.0516, 1.013
$\Delta\rho_{max}$, $\Delta\rho_{min}$ (e $Å^{-3}$)	0.449, -0.530	0.527, -1.059	0.418, -0.458

Table S8: Crystallographic details of complexes **[3]**, **[4]** and **$[3\cdot MeCN](BF_4)_2$**.

	**[Cp*Rh(aphab)]**	**[Cp*Rh(bphab)]**	**$[Cp^*Rh(bphiq)\cdot MeCN](BF_4)_2\cdot CH_3CN$**
Chemical formula	$C_{22}H_{25}N_2Rh$	$C_{28}H_{29}N_2Rh$	$C_{32}H_{35}B_2F_8N_4Rh$
M_r	420.35	496.44	752.17
Crystal system	Monoclinic	Monoclinic	Monoclinic
Space group	$P2_1/c$	$P2_1/n$	$P2_1/c$
a (Å)	10.494(1)	19.096(3)	13.237(1)
b (Å)	11.475(2)	10.305(1)	12.762(1)
c (Å)	15.747(1)	23.860(1)	19.660(1)
□□(°)	90	90	90
β (°)	90.1(1)	104.5(1)	92.7(1)
□ (°)	90	90	90
V ($Å^3$)	1896.1(2)	4547(2)	3317.5(3)
Z	4	8	4
Densitiy (g cm^{-3})	1.473	1.451	1.506
F(000)	864	2048	1528
Radiation Type	MoK□	MoK□	MoK□
μ (mm^{-1})	0.907	0.769	0.589
Crystal size	0.40 x 0.21 x 0.14	0.56 x 0.431 x 0.104	0.43 x 0.24 x 0.20
Meas. Refl.	41261	40214	89550
Indep. Refl.	5805	11261	12669
Obsvd. [$I > 2\sigma(I)$] refl.	5126	6682	10757
R_{int}	0.0345	0.0632	0.0257
R [$F^2 > 2\sigma(F^2)$], wR(F^2), S	0.0233, 0.0521, 1.061	0.0619, 0.1615, 1.057	0.0258, 0.0709, 1.060
$\Delta\rho_{max}$, $\Delta\rho_{min}$ (e $Å^{-3}$)	0.550, -0.415	1.99,-1.38	0.642, -0.435

Table S9: Crystallographic details of complexes **[CoCp*₂][1]**, **[CoCp*₂][1]** and **[5](BF₄)₂**.

	**[CoCp*$_2$][Cp*Rh(bmsab)] ·$C_4H_{10}O$**	**[CoCp*$_2$][Cp*RhCl(bmsab)] ·CH_3CN**	**[Cp*Rh(bphiq)RhCp*] (BF_4)$_2$**
Chemical formula	$C_{42}H_{65}CoN_2O_5RhS_2$	$C_{40}H_{58}ClCoN_3O_4RhS_2$	$C_{38}H_{44}B_2F_8N_2Rh_2$
M_r	903.92	906.3	908.19
Crystal system	Monoclinic	Monoclinic	Monoclinic
Space group	P2$_1$	P2$_1$/c	P2$_1$/c
a (Å)	11.728 (1)	9.501 (1)	12.976 (1)
b (Å)	16.457 (1)	26.108 (2)	16.873 (2)
c (Å)	12.111 (1)	17.112 (1)	18.288 (2)
□□(°)	90	90	90
β (°)	113.8 (1)	103.0 (1)	99.5 (1)
□ (°)	90	90	90
V (Å^3)	2138.7 (2)	4135.7 (3)	3948.9(4)
Z	2	8	4
Densitiy (g cm^{-3})	1.404	1.456	1.528
F(000)	950	1888	1832
Radiation Type	MoK□	MoK□	MoK□
μ (mm^{-1})	0.915	1.009	0.902
Crystal size	0.66 x 0.32 x 0.25	0.33 x 0.31 x 0.16	0.75 x 0.33 x 0.07
Meas. Refl.	57767	48602	53346
Indep. Refl.	13015	10303	9848
Obsvd. [$I > 2\sigma(I)$] refl.	12193	7661	7158
R_{int}	0.0264	0.0448	0.0584
R [$F^2 > 2\sigma(F^2)$], wR(F^2), S	0.0242, 0.0556, 1.032	0.0439, 0.1073, 1.039	0.0393, 0.0913, 1.014
$\Delta\rho_{max}$, $\Delta\rho_{min}$ (e Å^{-3})	0.492, -0.544	1.809, -1.154	1.156, -0.693

Additional Bond Lengths and Angles

Table S10: Selected bond lengths, bond angles and dihedral angles in neutral complexes **[1]** - **[4]**. Bond lengths given in Å.

	**[Rh1]**	**[Rh1·MeCN]**	**[Rh2]**	**[Rh3]**	**[Rh4]**
C1-C2	1.417(3)	1.413(2)	1.418(3)		1.418(2)
C2-C3	1.398(2)	1.398(3)	1.403(3)		1.403(3)
C3-C4	1.388(2)	1.394(2)	1.386(3)		1.375(3)
C4-C5	1.397(3)	1.380(2)	1.395(3)		1.402(3)
C5-C6	1.388(2)	1.393(2)	1.390(3)		1.379(3)
C6-C1	1.398(2)	1.399(2)	1.401(3)		1.400(2)
C1-N1-R1	113.57(7)°	120.84(9)°	118.27(14)°		118.18(13)°
C1-N1-Rh	116.21(9)°	113.43(8)	114.90(13)°		117.11(10)°
R1-N1-Rh	123.27(7)	124.39(6)	126.28(10)°		124.7(1)°
C2-N2-R2	113.57(7)°	119.43(8)°	112.7(19)°		116.4(14)°
C2-N2-Rh	116.21(9)°	112.79(7)°	120.16(14)°		118.31(11)°
R2-N2-Rh	123.27(7)	123.81(6)°	127.1(19)°		125.3(14)°
N1-Rh-N2	76.81(7)°	77.37(4)°	77.93(7)°		78.32(6)°
C1-N1-R1-Rh	170.8°	167.4°	171.3°		179.3°
C2-N2-R2-Rh	170.8°	157.3°	179.2°		177.5°

Table S11: Selected bond lengths, bond angles and dihedral angles in complex **[CoCp*2][1]**. Bond lengths given in Å.

	**[CoCp*$_2$][Rh1]**
Rh-N1	2.096(3)
Rh-N2	2.102(3)
N1-C1	1.403(4)
N2-C2	1.415(4)
C1-C2	1.412(4)
C2-C3	1.403(4)
C3-C4	1.380(5)
C4-C5	1.370(5)
C5-C6	1.385(4)
C6-C1	1.400(4)
C1-N1-R1	119.2(2)°
C1-N1-Rh	114.35(19)°
R1-N1-Rh	126.32(1)°
C2-N2-R2	119.2(2)°
C2-N2-Rh	114.40(19)°
R2-N2-Rh	125.75(16)°
N1-Rh-N2	76.27(6)°
C1-N1-R1-Rh	175.3°
C2-N2-R2-Rh	174.4°

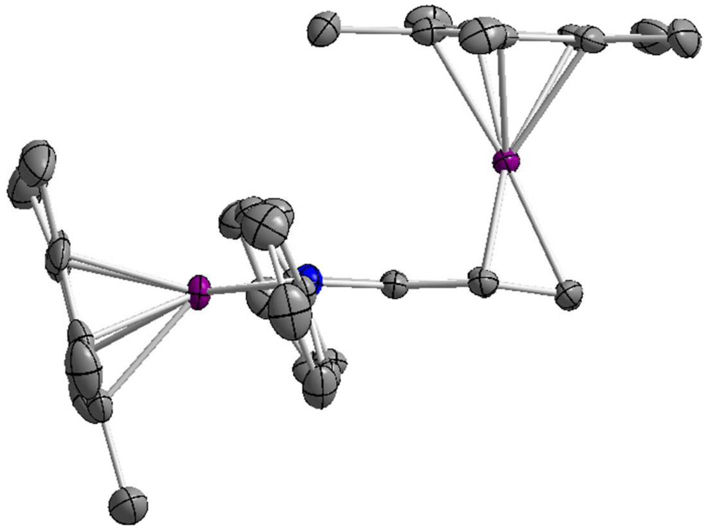

Figure S37: Side view of complex **[5](BF$_4$)$_2$**. Anions and H atoms omitted for clarity. Ellipsoids drawn at 50 % probability.

References

1 G. R. Fulmer, A. J. M. Miller, N. H. Sherden, H. E. Gottlieb, A. Nudelman, B. M. Stoltz, J. E. Bercaw and K. I. Goldberg, NMR Chemical Shifts of Trace Impurities: Common Laboratory Solvents, Organics, and Gases in Deuterated Solvents Relevant to the Organometallic Chemist, *Organometallics*, 2010, **29**, 2176–2179.

2 G. M. Sheldrick, SHELXT - integrated space-group and crystal-structure determination, *Acta crystallographica. Section A, Foundations and advances*, 2015, **71**, 3–8.

3 G. M. Sheldrick, A short history of SHELX, *Acta crystallographica. Section A, Foundations of crystallography*, 2008, **64**, 112–122.

4 G. M. Sheldrick, Crystal structure refinement with SHELXL, *Acta Cryst C*, 2015, **71**, 3–8.

5 J. R. Aranzaes, M.-C. Daniel and D. Astruc, Metallocenes as references for the determination of redox potentials by cyclic voltammetry   Permethylated iron and cobalt sandwich complexes, inhibition by polyamine dendrimers, and the role of hydroxy-containing ferrocenes, *Can. J. Chem.*, 2006, **84**, 288–299.

6 M. Krejčik, M. Daněk and F. Hartl, Simple construction of an infrared optically transparent thin-layer electrochemical cell, *Journal of Electroanalytical Chemistry and Interfacial Electrochemistry*, 1991, **317**, 179–187.

7 S. Stoll and A. Schweiger, EasySpin, a comprehensive software package for spectral simulation and analysis in EPR, *Journal of magnetic resonance (San Diego, Calif. : 1997)*, 2006, **178**, 42–55.

8 F. Neese, Software update: the ORCA program system, version 4.0, *WIREs Comput Mol Sci*, 2018, **8**. DOI: 10.1002/wcms.1327.

9 F. Neese, The ORCA program system, *WIREs Comput Mol Sci*, 2012, **2**, 73–78.

10 F. Weigend and R. Ahlrichs, Balanced basis sets of split valence, triple zeta valence and quadruple zeta valence quality for H to Rn: Design and assessment of accuracy, *Physical chemistry chemical physics : PCCP*, 2005, **7**, 3297–3305.

11 V. N. Staroverov, G. E. Scuseria, J. Tao and J. P. Perdew, Comparative assessment of a new nonempirical density functional: Molecules and hydrogen-bonded complexes, *The Journal of Chemical Physics*, 2003, **119**, 12129–12137.

12 S. Grimme, J. Antony, S. Ehrlich and H. Krieg, A consistent and accurate ab initio parametrization of density functional dispersion correction (DFT-D) for the 94 elements H-Pu, *The Journal of Chemical Physics*, 2010, **132**, 154104.

13 F. Neese, F. Wennmohs, A. Hansen and U. Becker, Efficient, approximate and parallel Hartree–Fock and hybrid DFT calculations. A 'chain-of-spheres' algorithm for the Hartree–Fock exchange, *Chemical Physics*, 2009, **356**, 98–109.

14 O. Vahtras, J. Almlöf and M. W. Feyereisen, Integral approximations for LCAO-SCF calculations, *Chemical Physics Letters*, 1993, **213**, 514–518.

15 F. Neese and G. Olbrich, Efficient use of the resolution of the identity approximation in time-dependent density functional calculations with hybrid density functionals, *Chemical Physics Letters*, 2002, **362**, 170–178.

16 R. Izsák and F. Neese, An overlap fitted chain of spheres exchange method, *The Journal of Chemical Physics*, 2011, **135**, 144105.

17 K. Eichkorn, O. Treutler, H. Öhm, M. Häser and R. Ahlrichs, Auxiliary basis sets to approximate Coulomb potentials, *Chemical Physics Letters*, 1995, **240**, 283–290.

18 K. Eichkorn, F. Weigend, O. Treutler and R. Ahlrichs, Auxiliary basis sets for main row atoms and transition metals and their use to approximate Coulomb potentials, *Theor Chem Acta*, 1997, **97**, 119–124.
19 F. Weigend, Accurate Coulomb-fitting basis sets for H to Rn, *Physical chemistry chemical physics : PCCP*, 2006, **8**, 1057–1065.
20 H. Stetter, Ein neues Prinzip zur Darstellung höhergliedriger Ringsysteme, I. Mitteil.: Ringschluß-Reaktionen bei Sulfonamiden des o -Phenylendiamins, *Chem. Ber.*, 1953, **86**, 197–205.
21 S. Fu, H. Jiang, Y. Deng and W. Zeng, Palladium-Catalyzed Intramolecular Sulfonamidation/Oxidation of Imines: Access to Multifunctional Benzimidazoles, *Adv. Synth. Catal.*, 2011, **353**, 2795–2804.
22 T. Wenderski, K. M. Light, D. Ogrin, S. G. Bott and C. J. Harlan, Pd catalyzed coupling of 1,2-dibromoarenes and anilines: formation of N,N-diaryl-o-phenylenediamines, *Tetrahedron Letters*, 2004, **45**, 6851–6853.
23 U. Kölle and F. Khouzami, Permethylmetallocene, II. Decamethylcobaltocen: Synthese und Umwandlung in methylierte (Aren)(cyclopentadienyl)cobalt-Kationen, *Chem. Ber.*, 1981, **114**, 2929–2937.
